# Highly Sensitive In Vivo Imaging of Bacterial Infections with a Hydrophilicity‐Switching, Self‐Immobilizing, Near‐Infrared Fluorogenic β‐Lactamase Probe Enriched within Bacteria

**DOI:** 10.1002/advs.202408559

**Published:** 2024-12-12

**Authors:** Fangfang Chen, Yuyao Li, Yan Peng, Yifan Zhu, Gao He, Zhengwei Zhang, Hexin Xie

**Affiliations:** ^1^ State Key Laboratory of Bioreactor Engineering Shanghai Key Laboratory of New Drug Design Frontiers Science Center for Materiobiology and Dynamic Chemistry Shanghai Frontier Science Research Base of Optogenetic Techniques for Cell Metabolism School of Pharmacy East China University of Science and Technology Shanghai 200237 P.R. China; ^2^ Department of Nuclear Medicine & PET Center Huashan Hospital, Fudan University Shanghai 200235 China

**Keywords:** β‐lactamases, antibiotic resistance, bacterial infections, fluorescence probes, in vivo imaging

## Abstract

The emergence of antibiotic resistance, particularly bacterial resistance to β‐lactam antibiotics, the most widely prescribed therapeutic agents for infectious diseases, poses a significant threat to public health worldwide. The discovery of effective therapies against antibiotic‐resistant pathogens has become an urgent need, necessitating innovative approaches to accelerate the identification and development of novel antibacterial agents. On the other hand, the expression of the β‐lactam‐hydrolyzing enzyme (β‐lactamase), the major cause of bacterial resistance to β‐lactam antibiotics, provides a distinctive opportunity to visualize bacterial infection, evaluate the efficacy of existing antibiotics, screen for novel antibacterial agents, and optimize drug dosing regimens in live animals. Herein, a hydrophilicity‐switching, self‐immobilizing, near‐Infrared fluorogenic β‐lactamase probe for the highly sensitive imaging of bacterial infection in live mice is reported. This probe, in addition to a significant increase in fluorescence upon selective hydrolysis by β‐lactamases as conventional β‐lactamase probes, also massively enriches within β‐lactamase‐expressing bacteria (over 1500‐folds compared to the incubation medium), which renders excellent sensitivity in the imaging of bacterial infections in living animals. This agent has proven to enable the assessment of antibiotic therapeutic efficacy and potency of β‐lactamase inhibitors in living animals in a non‐invasive and much more convenient manner.

## Graphical abstract

Reported herein is a novel hydrophilicity‐switching, self‐immobilizing, near‐infrared fluorogenic β‐lactamase probe, which, upon selective hydrolyzed by bacterial β‐lactamase, turns on fluorescence at 710 nm and effectively enriches within bacteria (1500‐folds higher than incubation medium), allowing highly sensitive imaging of infection by resistant bacteria in living animals with long‐lasting signal.

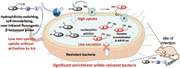

## 1 Introduction

The emergence of antibiotic resistance has posed a formidable health challenge globally.^[^ ^1^ ^]^ Particularly, the expression of β‐lactamase (bla) in pathogenic bacteria has caused severe bacterial resistance to β‐lactam antibiotics, significantly undermining the effectiveness of the most widely prescribed class of therapeutic agents for the treatment of infectious diseases.^[^ ^2^ ^]^ Furthermore, many β‐lactamase genes are encoded in plasmids. These circular DNA molecules can be transferred between bacteria easily, allowing the rapid dissemination of antibiotic resistance across different species of bacterial pathogens.

To compact antibiotic resistance caused by β‐lactamases, a number of strategies need to be implemented at multiple levels, including the development of novel antibacterial agents or new β‐lactamase inhibitors to reverse resistance to β‐lactam antibiotics, as well as approaches for point‐of‐care diagnostics at early‐stage bacterial infections. A significant challenge for these is the lack of a sensitive and accurate modality to visualize bacterial infections in vivo in a non‐invasive manner, which provides critical information on the kinetics of infection, host immune responses, and the effectiveness of antimicrobial treatments.^[^ ^3^ ^]^

Conventionally, the detection of bacteria relies on culture and colony‐counting‐based methods, which are not only labor‐intensive and time‐consuming but also incompatible with real‐time and continuous detection. A number of fluorescence‐based reagents have been developed as non‐invasive tools for in vivo imaging of bacterial infections,^[^ ^4^ ^]^ which utilize antibiotics (e.g., vancomycin,^[^ ^5^ ^]^ polymyxin,^[^ ^6^ ^]^ and teicoplanin^[^ ^7^ ^]^), sugar molecules (e.g., maltotriose,^[^ ^8^ ^]^ maltodextrin,^[^ ^3a^ ^]^ and trehalose^[^ ^9^ ^]^), boronic acid,^[^ ^10^ ^]^ or antibodies^[^ ^11^ ^]^ as bacteria‐recognition moieties. Most of these molecules, however, lack the ability to amplify fluorescence signals upon interaction with target bacteria and thus limit the detection sensitivity.

Given the exceptionally high catalytic efficiency on the hydrolysis of β‐lactam antibiotics, the expression of β‐lactamase in bacteria, while contributing to antibiotic resistance, also presents a distinctive opportunity to report or even selectively eradicate resistant bacteria in living organisms.^[^ ^12^ ^]^ To date, a large number of β‐lactamases fluorescent substrates have been developed^[^ ^13^ ^]^ since the pioneer work by Tsien's group in 1998.^[^ ^14^ ^]^ Most of these reagents proved to be highly sensitive and selective in the detection of β‐lactamase activities in solution‐based tests. Nevertheless, the use of fluorescent probes to monitor bacterial infections in living animals still remains challenging, which is largely attributed to the fact that β‐lactamases are a type of enzyme mainly located in the periplasmic space of bacteria or excreted^[^ ^2b^ ^]^ and the intricate and dynamic nature of living animals.

The imaging of β‐lactamase activity in living animals using fluorescence probes, particularly by intravenous administration, not only relies on the enhancement of fluorescence intensity upon activation by target enzyme as in in vitro tests but also hinges on the effective retention, ideally enrichment, of the activated fluorophore at the site of interest.^[^ ^15^ ^]^ Furthermore, these imaging reagents must possess sufficient hydrophilicity to facilitate delivery to the detection site through intravenous administration. Additionally, the presence of excitation/emission in the near‐infrared (NIR) region is also crucial for the reagents used for in vivo imaging, which helps to minimize interference from auto‐fluorescence and enables deeper tissue penetration.^[^ ^15^, ^16^ ^]^

The CNIR series of probes are among the first type of probes that have been shown to be compatible with the imaging of β‐lactamase activity in living animals.^[^ ^17^ ^]^ These types of β‐lactamase substrates used highly hydrophilic NIR fluorophores and quenchers to report the activity of the enzyme and fully acetylated *D‐*glucosamine to enhance the retention of the activated fluorophore at the sites of detection. However, further application of these structurally complex probes in biological studies has been largely limited by the synthetic challenges. Recently, a structurally concise β‐lactamase NIR fluorescent probe CySG‐2 was reported by Li's group, but this reagent appeared to be compatible only with local administration in living mice, probably related to the extremely low hydrophilicity of the probe.^[^ ^18^ ^]^

In this study, we integrate the design of conventional β‐lactamase–activatable fluorescence probe with quinone methide (QM)‐based self‐immobilization strategy and hydrophilicity‐switching design^[^ ^15c^ ^]^ to report a novel β‐lactamase NIR fluorogenic probe for the imaging of bacterial infections in living mice. This reagent, in addition to enhancement in fluorescence emission after selective hydrolysis by β‐lactamase as conventional fluorogenic probes, also results in effective enrichment in β‐lactamase‐expressed bacteria due to the switching of hydrophilicity and formation of covalent bond with macromolecules in bacteria. These features make it particularly appealing in the in vivo imaging. We have demonstrated that this hydrophilicity‐switchable, self‐immobilizing, NIR fluorogenic probe is highly sensitive in the in vivo imaging of bacterial infection with long‐lasting signal at the detection sites, allowing rapid assessment of antibiotic therapeutic efficacy and potency of β‐lactamase inhibitor in living animals.

## 2 Results and Discussion

### 2.1 Design and Synthesis

**Figure 1:**
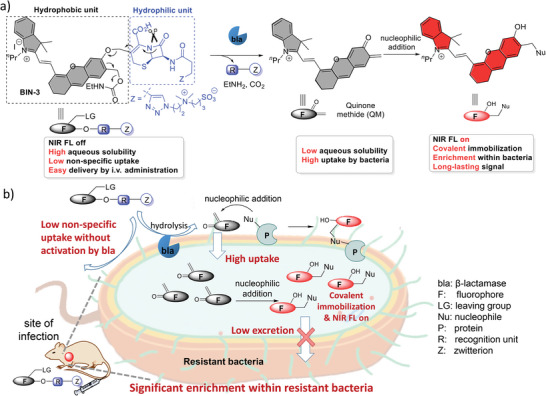
Chemical structure of probe **BIN‐3** and schematic illustration of the activation and enrichment of NIR probe within bla‐expressing resistant bacteria through a hydrophilicity‐switching and self‐immobilizing design.

Unlike in vitro imaging, the efficacy of fluorescent probes designed for in vivo imaging in living animals, especially via intravenous administration, is closely related to their hydrophilicity; low hydrophilicity likely impedes delivery to the site of interest, while high hydrophilicity often results in poor retention at the site of detection. In this study, we designed a hydrophilicity‐switchable, self‐immobilizing, NIR fluorogenic probe, **BIN‐3**, for the in vivo detection of bacterial infection. As most of the reported β‐lactamase fluorescent probes, **BIN‐3** consists of a cephalosporin as β‐lactamase substrate and an HD dye^[^ ^19^ ^]^ as a fluorescent reporter (**Figure** **1**). Moreover, a hydrophilic zwitterion is conjugated on the cephalosporin to render high aqueous solubility and low non‐specific uptake of the probe, and a carbamate group as the leaving group is installed on the hydrophobic HD dye.^[^ ^15^ ^]^ We envisaged the selective hydrolysis by β‐lactamase should lead to the releasing of all hydrophilic modules (hydrophilic zwitterion and cephalosporin) and the carbamate group, forming highly hydrophobic quinone methide^[^ ^20^ ^]^ (QM) intermediate. The switching from hydrophilic to hydrophobic effectively restricts the rapid diffusion of these reactive QM intermediates from the site of activation and increases uptake by bacteria, and thus allows them to react with nucleophilic residues, mainly thiol group, from β‐lactamase or nearby proteins, or intracellular nucleophiles after internalization by bacteria. The formation of covalent bonds between activated fluorophores and bacteria effectively blocks the excretion of these molecules from bacteria. This results in significant enrichment within resistant bacteria and switching‐on of NIR fluorescence emission, and thus long‐lasting fluorescence signal at detection sites.

The synthesis of **BIN‐3** began with the nucleophilic addition of cephalosporin **2** by HD dye **1** ^[^ ^15c^ ^]^ (Scheme S1, Supporting Information). However, the use of potassium carbonate as a base, as previously reported,^[^ ^13e^ ^]^ resulted in the formation of a mixture of Δ_2 _− Δ_3_ isomers, which were difficult to separate using regular silica gel chromatography. The use of a weaker base, potassium bicarbonate, led to extremely sluggish conversion. Fortunately, the addition of 18‐crown‐6 to this reaction facilitated the formation of compound **3** without the formation of the Δ_3_ isomer. To prevent the formation of the Δ_3_ isomer, the subsequent reaction of **3** and ethyl isocyanate was performed in the presence of dibutyltin dilaurate (DBTDL) instead of organic bases. After a TFA‐mediated deprotection and a CuAAC click reaction,^[^ ^21^ ^]^ probe **BIN‐3** was obtained. As controls, leaving group‐free probes **BIN‐1**, **BIN‐2** and probe **1^[^** ^13e^ ^]^ were prepared similarly (Schemes S2,S3, Supporting Information).

### 2.2 In Vitro Characterization

**Figure 2:**
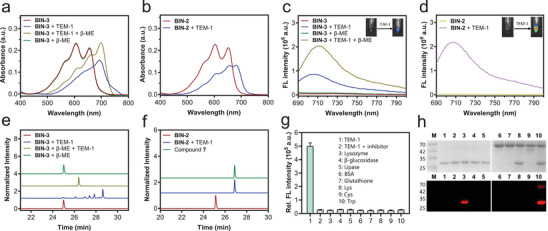
In vitro characterization of β‐lactamase probes **BIN‐3** and **BIN‐2**. a,b) UV–vis spectra of **BIN‐3** (a) and **BIN‐2** (b) before and after incubation with TEM‐1 bla. c,d) Fluorescence spectra of **BIN‐3** (c) and **BIN‐2** (d) before and after incubation with TEM‐1 bla. The probe (5 µm in PBS, pH 7.4) was incubated with TEM‐1 bla (100 nm) in the presence or absence of β‐mercaptoethanol (10 mm) at 37 °C for 15 min. The inset shows the pictures with *λ*_ex/em _= 687/714 nm. e,f) HPLC traces at 660 nm of **BIN‐3** (e) or **BIN‐2** (f) before and after incubation with TEM‐1 (100 nm) or β‐mercaptoethanol (10 mm) at 37 °C for 30 min. g) Fluorescence response of **BIN‐3** (10 µm) to various analytes in PBS. 1: TEM‐1 (10 nm); 2: TEM‐1 (10 nm) + inhibitor (100 µm); 3: lysozyme (2 U mL^−1^); 4: β‐glucosidase (5 µm); 5: Lipase (2 U mL^−1^); 6: BSA (10 µm); 7: glutathione (2 µm); 8: Lys (1 mm); 9: Cys (1 mm); 10: Trp (1 mm). *λ*_ex_/*λ*_em _= 687/714 nm; data represent mean ± SD (*n* = 3). h) Coomassie blue staining (top) and fluorescence imaging (bottom) of SDS‐PAGE gel. The indicated protein was incubated with or without probes in PBS at 37 °C for 2 h. M: protein marker; 1: TEM‐1; 2: TEM‐1 + **BIN‐2**; 3: TEM‐1 + **BIN‐3**; 4: TEM‐1 + **BIN‐2** + inhibitor; 5: TEM‐1 + **BIN‐3 **+ inhibitor; 6: BSA; 7: BSA + **BIN‐2**; 8: BSA + **BIN‐2** + TEM‐1; 9: BSA + **BIN‐3**; 10: BSA + **BIN‐3** + TEM‐1. *λ*_ex/em _= 685/720 nm; inhibitor = Avibactam.

With these reagents in hand, we first investigated the response of **BIN‐1**, **BIN‐2**, and **BIN‐3** to β‐lactamase by recording their absorption spectra before and after incubation with β‐lactamase. **BIN‐1** and probe **1** appeared to have much lower solubility in PBS (pH 7.4) compared to the zwitterion‐containing probe **BIN‐2** (Figure S1, Supporting Information), which makes it less suitable for the detection of β‐lactamase, especially in in vivo study. The testing of **BIN‐1** with β‐lactamase was performed in PBS containing 10% DMSO. **Figures** **2** and S1 (Supporting Information) show that all of the three reagents, **BIN‐1**, **BIN‐2,** and **BIN‐3** experienced a significant red‐shift in absorption after incubation with TEM‐1 bla, a type of class A β‐lactamase commonly found in Gram‐negative bacteria.^[^ ^22^ ^]^ However, the absorption of **BIN‐3** treated with β‐lactamase occurred at an even longer wavelength. This may be due to the formation of a QM intermediate after hydrolysis by TEM‐1 bla. The addition of free thiol, β‐mercaptoethanol (β‐ME), to this mixture, resulted in an increase in absorption at ≈700 nm, suggesting that the presence of a strong nucleophile facilitates the transformation of the QM intermediate into free HD dye. Notably, the incubation of **BIN‐3** with β‐ME in the absence of TEM‐1 bla did not result in any detectable changes, indicating that this probe is stable to free thiol before enzymatic activation.

The fluorescence spectra of the probes were also recorded. As shown in Figure 2 and Figures S2 and S3 (Supporting Information), upon incubation with TEM‐1 bla alone, fluorescently silent **BIN‐3** turned on fluorescence emission at ≈710 nm but with lower intensity compared to that of TEM‐1 bla‐treated **BIN‐2**. Furthermore, the addition of β‐ME to the TEM‐1‐incubated **BIN‐3** further enhanced the fluorescence intensity to a degree similar to that of TEM‐1‐treated **BIN‐2**. The addition of β‐ME alone to **BIN‐3** did not activate fluorescence emission.

To investigate the enzyme‐mediated hydrolysis process of **BIN‐3**, we analyzed the reaction mixture using HPLC and mass spectrometry. In contrast to the TEM‐1‐incubated **BIN‐2**, which produced only free HD dye (Figure 2f), **BIN‐3** gave rise a mixture of compounds with the characteristic absorption of HD dye upon incubation with TEM‐1 (Figure 2e). This is likely due to the nucleophilic addition of various nucleophiles, including water, to the **BIN‐3**‐releasing QM intermediate after hydrolysis by TEM‐1. To mimic bio‐thiols, β‐ME was added to the TEM‐1‐treated **BIN‐3**, resulting a single component as the predominant product. Further analysis of this compound with HR‐MS confirmed the structure of this compound to the β‐ME adduct **1S** (Figure S4, Supporting Information). These results demonstrate the high nucleophilicity of free thiol to react with QM. As a result, free bio‐thiols from resistant bacteria may act as the primary nucleophiles to react with **BIN‐3**‐originated QM. The incubation of **BIN‐3** with β‐ME without β‐lactamase did not produce any alterations in HPLC analysis, supporting the high stability of this molecule to free bio‐thiols before enzymatic activation.

As shown in Figure 2g, the specificity of **BIN‐3** was further investigated by testing it with a variety of biologically important analytes, including catalytic proteins such as lysozyme, β‐glucosidase, and lipase, a non‐catalytic protein like bovine serum albumin (BSA), and small molecules such as glutathione, lysine, cysteine, and tryptophan. None of these led to an obvious increase in fluorescence intensity, demonstrating the high specificity of the probe. The addition of avibactam,^[^ ^23^ ^]^ a highly efficient inhibitor for TEM‐1 bla, to the TEM‐1‐treated **BIN‐3** effectively blocked the enhancement of fluorescence signal, supporting the activity of TEM‐1 as the sole cause for the turn‐on of fluorescence emission. Similar results were obtained when probe **BIN‐2** was tested (Figure S3, Supporting Information).

The covalent labeling ability of **BIN‐3** with enzyme was next studied using in‐gel fluorescence imaging. Figure 2h shows that, upon treatment with the self‐immobilizing probe **BIN‐3**, intense NIR fluorescence was emitted from the band of TEM‐1, while pre‐incubation of TEM‐1 with inhibitor (avibactam) before incubation with **BIN‐3** effectively suppressed the emission of fluorescence signal from TEM‐1. The co‐incubation of **BIN‐3** with TEM‐1 and non‐catalytic BSA resulted in fluorescence emission in both proteins. However, the signal on TEM‐1 bla was more intense compared to BSA. In contrast, the incubation of **BIN‐3** with BSA alone did not yield any fluorescence on the protein. These results confirm the covalent labeling ability of **BIN‐3** to protein and demonstrate its high specificity to β‐lactamase. The TEM‐1 bla incubated with probe **BIN‐2** remained non‐fluorescent, although this enzyme could effectively hydrolyze **BIN‐2** to release a free hydrophobic fluorophore.

To further confirm the selectivity of the probe toward β‐lactamase, we incubated probes with lysate of bla‐expressing *Enterobacter cloacae* (*E. cloacae*) and bla‐negative *Escherichia coli* (*E. coli*), respectively. The in‐gel fluorescence image showed that strong NIR fluorescence was only detected from the **BIN‐3**‐incubated lysate of bla‐positive bacteria, whereas incubation of bla‐negative bacteria with the same probe resulted in no fluorescence emission (Figure S5, Supporting Information). Moreover, the addition of the bla inhibitor avibactam to the lysate of bla‐positive bacteria incubated with **BIN‐3** dramatically reduced the intensity of fluorescence emission. These results clearly demonstrate that the covalent labeling of bacteria by **BIN‐3** is a highly β‐lactamase‐selective process. On the other hand, the **BIN‐2**‐treated sample remained fluorescently silent regardless of the effective activation of this probe by β‐lactamase.

Having validated the fluorescence‐activation and covalent binding ability of **BIN‐3** by β‐lactamase, the major cause of bacterial resistance, we thus moved to adopt this probe for the imaging of resistant bacteria. We first assessed the biocompatibility of probes **BIN‐2** and **BIN‐3** by investigating the impact of these molecules on the growth of bacteria. As can be seen from Figure S6 (Supporting Information), the addition of **BIN‐2** or **BIN‐3** did not seem to interfere the growth of bla‐negative *E. coli* and bla‐positive *E. cloacae*, both of which belong to Gram‐negative bacteria. Additionally, the cytotoxicity of **BIN‐2** and **BIN‐3** against mammalian cells was also tested using a standard CCK‐8 assay and it resulted in no detectable toxicity to HEK293 cells, a type of human embryonic kidney cells, at the concentration of up to 100 µm (Figure S7, Supporting Information). Furthermore, the hemolysis to mouse red blood cells was also investigated and both of these fluorogenic probes, **BIN‐2** and **BIN‐3,** showed no hemolysis even at the concentration of 100 µm (Figure S8, Supporting Information), manifesting the high biocompatibility of these imaging reagents.

Before imaging resistant bacteria with **BIN‐2** and **BIN‐3**, we monitored the fluorescence response of these probes to bla‐expressing bacteria using a fluorimeter. As expected, both NIR probes showed a significant increase in fluorescence intensity in the presence of bla‐positive *E. coli* or *E. cloacae*. In contrast, incubation with antibiotic‐susceptible *E. coli* did not trigger the emission of probes (Figure S9, Supporting Information).

### 2.3 Imaging of β‐Lactamase‐Expressing Resistant Bacteria

**Figure 3:**
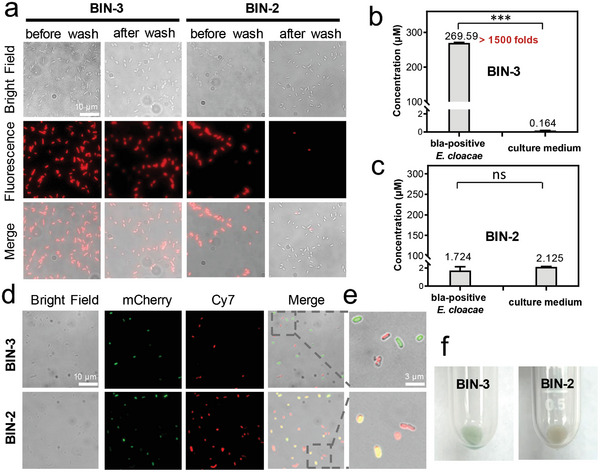
Fluorescence imaging of live bla‐expressing bacteria with **BIN‐3**. a) Fluorescence imaging of *E. cloacae* with **BIN‐3** or **BIN‐2** before and after washing. b,c) Concentration of **BIN‐3** (b) and **BIN‐2** (c) or their derivatives in bla‐expressing *E. cloacae* and incubation medium after incubation with these probes (2.5 µm). d) Fluorescence imaging of a mixture of bla‐expressing *E. cloacae* and bla‐negative *E. coli* (encoded with mCherry, labeled as green fluorescence) with **BIN‐3** or **BIN‐2** (20 µm). e) Amplified images. f) Photograph of a pellet of bla‐expressing *E. cloacae* upon incubation with **BIN‐3** or **BIN‐2**. Data represent mean ± SD (*n *= 3); ns: not significant; * *P* < 0.05; ** *P*  < 0.01; *** *P* < 0.001.

Microscopic imaging of bacteria was performed next. As depicted **Figures** **3** and S10 (Supporting Information), incubation of ampicillin‐resistant *E. cloacae* with **BIN‐2** or **BIN‐3** without washing away free fluorophores resulted in obvious NIR fluorescence emission on bacteria. The observed fluorescence was effectively blocked by pre‐treatment with avibactam, a potent β‐lactamase inhibitor. Similar results were observed when bla‐expressing *E. coli* was used (Figure S11, Supporting Information). As a control, bla‐negative *E. coli*, upon treatment with **BIN‐2** or **BIN‐3** under identical conditions, remained non‐fluorescent.

However, upon washing with PBS, the fluorescence signal from the **BIN‐2**‐treated *E. cloacae* decreased significantly while the fluorescence from those treated with **BIN‐3** remained largely unchanged. These results evidently demonstrate **BIN‐3** can effectively trap in resistant bacteria, likely in a covalent manner, after enzymatic activation, and **BIN‐2**, in contrast, is prone to diffuse out under identical conditions regardless the high hydrophobicity of the fluorophore (**7**) resulted from the hydrolysis of **BIN‐2**.

The high retention efficiency of **BIN‐3** in detection targets should not only increase the detection sensitivity in dynamic in vivo environments but also allow selectively label individual β‐lactamase‐expressing bacteria in the presence of negative bacteria. To test this possibility, we incubated this probe with a mixture of bacteria (bla‐positive *E. cloacae* and bla‐negative *E. coli*). Since these bacteria are morphologically indistinguishable, we used mCherry‐encoded bla‐negative *E. coli*, which emit fluorescence at 610 nm (labeled as green), in this study to differentiate between these two types of bacteria. Upon incubating **BIN‐3** with a mixture of these bacteria, the wash‐free images (Figure 3e) showed the bla‐positive *E. cloacae* was selectively labeled with NIR fluorescence (shown in red), whereas the mCherry fluorescence‐emitting bla‐negative *E. coli* (shown in green) remained silent at this NIR channel. And we observed no cross‐labeling at all. These results clearly demonstrate the high specificity of **BIN‐3** on labeling of individual bla‐expressing resistant bacteria, even in the presence of other negative bacteria. The high labeling specificity of **BIN‐3** in bla‐positive bacteria after enzymatic activation is obviously attributed to the forming covalent bonds with resistant bacteria, effectively limiting the diffusion of the activated fluorophore to other bla‐negative bacteria. In contrast, under identical conditions, **BIN‐2** led to obvious cross‐labeling in fluorescence; obvious NIR fluorescence emission could be detected from both bla‐negative *E. coli* (shown in yellow) and bla‐positive *E. cloacae*. Since **BIN‐2** proved to be non‐fluorescent upon treatment with bla‐negative *E. coli* alone (Figures S10,S11, Supporting Information), the NIR fluorescence observed in bla‐negative *E. coli* is likely resulted from the diffusion of β‐lactamase‐activated fluorophores from bla‐positive *E. cloacae* to negative bacteria, even though these activated fluorophores are much more hydrophobic compared to intact probe **BIN‐2**.

To investigate the labeling efficiency of **BIN‐3** on resistant bacteria, we isolated the pellet of **BIN‐3**‐incubated bla‐positive *E. cloacae* from the incubation medium by centrifugation, followed by washing with PBS. Upon analysis by fluorimeter, the incubation medium appeared to be basically non‐fluorescent until the addition of extra TEM‐1 and BSA (Figure S12, Supporting Information), suggesting the HD dye found in the medium was mainly in an unactivated form. On the basis of fluorescence intensity at 714 nm after enzymatic hydrolysis, we estimated the concentration of HD dye in the incubation medium to be ≈0.164 µm, significantly lower than its initial incubation concentration (2.5 µm) (Figure 3b).

The bacterial pellet looked blue‐green, the characteristic color of HD dye, implying the enrichment of HD dye on or with the bacteria (Figure 3f). The fluorescence spectrum of the bacterial pellet after lysis was consistent with the enzyme‐based spectrum (Figure S13, Supporting Information). The addition of extra TEM‐1 to the bacteria lysate did not result in any further enhancement in fluorescence intensity, confirming that the fluorophore in the pellet was already in an activated form. Based on the fluorescence intensity at 714 nm, each colony‐forming unit (CFU) of *E. cloacae* was estimated to be labeled by ≈6.39 × 10^4^ HD fluorophores. The concentration of HD fluorophores in the *E. cloacae* was calculated to be ≈0.270 mm (the volume of each CFU of *E. cloacae* was estimated to be ≈0.4 fL^[^ ^24^ ^]^), over 1500‐folds higher than the incubation medium (269.59 µm vs 0.164 µm). The incubation of **BIN‐3** with bla‐negative *E. coli*, in contrast, resulted in this reagent predominantly remaining in the incubation medium in an unactivated form (Figure S14, Supporting Information). These data confirm that the selective hydrolysis of **BIN‐3** by β‐lactamase in *E. cloacae* not only turned on the NIR fluorescence emission but also resulted in significant enrichment of activated fluorophores in resistant bacteria. This feature is particularly useful in the detection of bla‐expressing bacteria in highly dynamic environments (i.e., living animals).

As a control, incubating *E. cloacae* with fluorogenic probe **BIN‐2** resulted in only ≈415 HD fluorophores being retained in each CFU of bacteria, or a relative concentration of HD fluorophores in/on bacteria of ≈1.724 µm (Figure 3c). This is 150 times lower than that of **BIN‐3**, despite their high structural similarity. Additionally, analysis of the incubation medium revealed that the majority of probe **BIN‐2** remained in the incubation medium, instead of being trapped in resistant bacteria like **BIN‐3** (Figure S11, Supporting Information).

Encouraged by the results in the in vitro tests, we turned out attention to the application of this reagent in in vivo imaging. Prior to the test on the infected mouse, we investigated the biosafety of these probes on healthy mice. Upon intravenous administration of **BIN‐3** or **BIN‐2** for 24 h, we examined a range of blood biochemical indexes of these mice, including white blood cells, red blood cells, hemoglobin, hematocrit, mean corpuscular volume, mean corpuscular hemoglobin, mean corpuscular hemoglobin concentration, platelet count, procalcitonin, total protein, albumin, and urea (Figure S15, Supporting Information), as well as the main organs by hematoxylin‐eosin (H&E) staining (Figure S16, Supporting Information). We observed no discernible difference between mice treated with **BIN‐2**, **BIN‐3**, and saline, demonstrating the high in vivo biocompatibility of these reagents.

### 2.4 In Vivo Imaging of β‐Lactamase Activity

**Figure 4:**
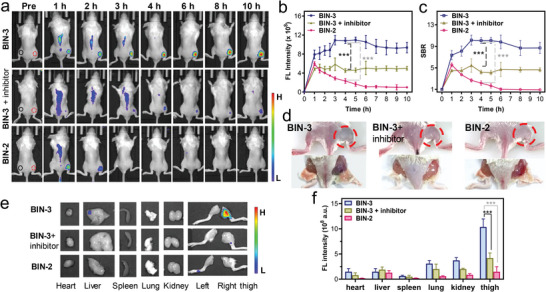
In vivo imaging of infection by bla‐expressing *E. coli* using **BIN‐3** or **BIN‐2**. a) Representative whole‐body fluorescence images mice with infection bla‐positive *E. coli* at the muscle of right rear thigh after *i.v*. administration of probes. For the imaging with inhibitor, bacteria was pretreated with Avibactam (a bla inhibitor) before being injected to the mouse. b) Time‐course fluorescence intensity at right thigh. c) Calculated signal‐to‐background ratio (SBR) of the site of infection at 710 nm. d) Photographs of infected mice at 10 h post‐injection of the probe. Top: with intact skin; bottom: without skin. e) Fluorescence images of major organs and infected right thigh at 10 h post injection. f) Quantified fluorescence intensity of major organs and infected right thigh showed in (e). *λ*_ex/em _= 660/710 nm. The black circle in each mouse indicates the position chosen as the background for the calculation of SBR. Data represent mean ± SD (*n* = 5); ns: not significant; * *P* < 0.05; ** *P* < 0.01; *** *P *< 0.001.

A murine infection model was constructed by injecting bla‐encoded *E. coli* (5 × 10^8^ CFU) into the muscle of the right rear thigh of the mouse as previously reported.^[^ ^3a^ ^]^ **BIN‐3** was then administered one hour later. Notably, owing to its favorable aqueous solubility, we could readily administer this probe to mice through the tail vein. As shown in **Figures** **4** and S17 (Supporting Information), a distinct fluorescence signal was observed at the site of bacterial infection one hour after administering **BIN‐3**. The fluorescence intensity increased constantly over the next few hours, reaching a signal‐to‐background ratio (SBR) of over 10 at 3 h post‐administration of the probe. Impressively, a comparable fluorescence signal was still detectable at the site of infection at 10 h post‐administration with an SBR of ≈9. The efficient retention of activated fluorophore at the detection site expands the window of observation and enhances both the signal‐to‐background ratio and detection sensitivity in imaging. Additionally, the efficient blocking of fluorescent emission by pre‐treating bacteria with avibactam, a bla inhibitor, supports the observed fluorescence resulting from the bla‐mediated hydrolysis of **BIN‐3**. As a control, we also tested **BIN‐2**, a fluorogenic and hydrophilicity‐switchable bla probe, which resulted in observable fluorescence at the site of bacterial infection 1 h after administration, although with lower intensity and SBR compared to **BIN‐3**. However, the fluorescence induced by **BIN‐2** decreased rapidly over time, and the SBR dropped to ≈1 at 5 h post‐administration. The significantly stronger fluorescence and SBR produced by **BIN‐3** compared to its analog **BIN‐2** suggests that the establishment of a covalent bond with bacteria or nearby proteins, in addition to the alteration in hydrophilicity, plays a crucial role in retaining the activated fluorophore at the detection site during in vivo imaging.

The ex vivo images of these mice at 10 hours post‐administration confirmed these results. Self‐immobilizing **BIN‐3** generated a much stronger fluorescence signal at the right thigh compared to **BIN‐2**. Additionally, the fluorescence emission from other major organs, such as the heart, liver, spleen, lung, and kidney, remained at a very low level, indicating the high specificity of these probes (Figure 4e; Figure S18, Supporting Information). When the thigh skin of the **BIN‐3**‐treated mice was removed, a distinctive blue‐greenish hue, indicative of the HD dye, was clearly discernible at the site of infection by naked eyes, which was invisible in the **BIN‐2**‐treated or the avibactam‐pretreated mice (Figure 4d; Figure S19, Supporting Information). The findings illustrate the remarkable retention efficiency of **BIN‐3** at activation sites in highly dynamic animals, which could be attributable to the novel QM‐based hydrophilicity‐switchable immobilization design.

Meanwhile, to gain further information on the circulation and metabolism of probes, we collected the urine of the infected mice at different time points after *i.v*. injection of **BIN‐3** or **BIN‐2**. We immediately noticed that the urine of **BIN‐2**‐treated mice was much bluer, the characteristic color of the HD fluorophore, than that of **BIN‐3**‐treated mice. Further analysis of these urine samples using IVIS fluorescence imaging (after activation by the addition of extra β‐lactamase and nucleophile) (Figure S20, Supporting Information) and HPLC (Figure S21, Supporting Information) confirmed there were much more NIR fluorophores in the urine from **BIN‐2**‐treated mice than that from **BIN‐3**‐treated mice. Furthermore, we did not detect intact probes in the HPLC traces of the urine, but rather the free NIR fluorophore or its derivatives, indicating that both **BIN‐2** and **BIN‐3** had been hydrolyzed by β‐lactamase. By 6 h after injection, we could barely detect any NIR fluorophores in the urine of both **BIN‐2**‐ and **BIN‐3**‐treated mice, suggesting most of the unbounded fluorophores had been cleared from the mice.

**Figure 5:**
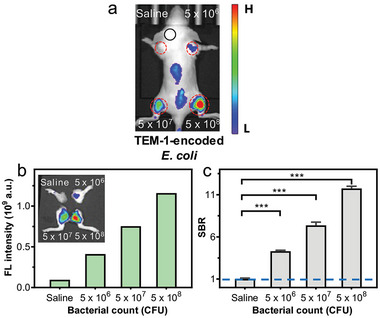
In vivo imaging of live mouse infected by various numbers of bla‐encoded *E. coli* at four thighs using **BIN‐3**. a) Whole‐body fluorescence image of the mouse at 5 h post administration of **BIN‐3**. b) Quantified fluorescence intensity of thighs at 5 h post‐administration. The inset shows the fluorescence images of the four thighs. c) Calculated SBR of the site of infection. *λ*_ex/em _= 660/710 nm. The black circle in each mouse indicates the position chosen as the background for the calculation of SBR. Data represent mean ± SD (*n* = 3); ns: not significant; * *P* < 0.05; ** *P* < 0.01; *** *P* < 0.001.

The sensitivity of **BIN‐3** in in vivo imaging of bacterial infection was further studied using mice infected by TEM‐1‐encoded *E. coli* at varying numbers (0, 5 × 10^6^, 5 × 10^7^ and 5 × 10^8^, or 0, 2 × 10^6^, 5 × 10^6^ and 2 × 10^7 ^CFUs) at four thighs, respectively. Upon *i.v*. administration of **BIN‐3**, the whole‐body fluorescence images show a strong correlation between the fluorescence intensity in these regions and the number of bacteria present (**Figure** **5**; Figures S22,S23, Supporting Information). Specifically, a higher number of bla‐expressing bacteria resulted in stronger fluorescence emission. Moreover, when the number of bacteria was decreased to 2 × 10^6^ CFUs, we were still able to detect a distinguishable fluorescence signal with an SBR of 3.0. These results support **BIN‐3** as a highly sensitive reagent in in vivo imaging of bacterial infection.

**Figure 6:**
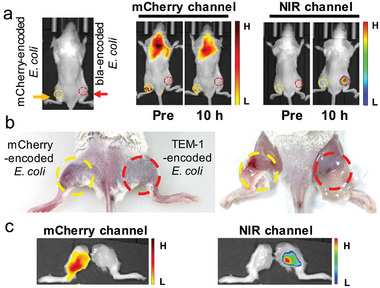
Selective imaging of bla‐positive *E. coli* in living mice with the left rear thigh infected by mCherry‐encoded *E. coli* (bla‐negative) and the right rear thigh infected by bla‐encoded *E. coli* after *i.v*. administration of **BIN‐3**. a) Whole‐body fluorescence images at 10 h post‐administration of the probe using the mCherry channel (*λ*_ex/em _= 580/620 nm) and NIR channel (*λ*_ex/em _= 660/710 nm), respectively. b) Photographs of infected thighs with or without skin at 10 h post‐administration. c) Fluorescence images of rear thighs using the mCherry and the NIR channel at 10 h post‐administration.

To investigate whether probe **BIN‐3** can differentiate bla‐positive bacteria from other susceptible bacteria in living mice, we tested this probe with mice infected by bla‐negative *E. coli* (encoded with mCherry) in the left rear thigh and bla‐positive *E. coli* (encoded with TEM‐1) in the right rear thigh. The subsequent intravenous administration of **BIN‐3** led to intense fluorescence emission at the NIR channel (710 nm) selectively from the right rear thigh of the mouse, the site of infection by bla‐encoded *E. coli*, with the left rear thigh (site of infection by mCherry‐encoded *E. coli*) remained fluorescently silent at the same channel (**Figure** **6**; Figure S24, Supporting Information). At the mCherry channel (620 nm), fluorescence could be detected from the left thigh, while the right thigh was non‐fluorescent. Notably, the imaging of mCherry‐expressing bacteria using this channel was significantly interfered by the background signal of mice, which might be associated with the relatively short emission wavelength. The ex vivo images of these mice were consistent with the in vivo images: **BIN‐3** selectively labeled bla‐expressing bacteria on the right thigh, while the left thigh emitted fluorescence only at the mCherry channel. Upon excision of the thigh skin of the mice, the characteristic blue‐greenish hue of HD dye was clearly visible to naked eyes on the right side (infected by bla‐expressing *E. coli*), while it is invisible on the left side (infected by mCherry‐expressing *E. coli*) (Figure 6; Figure S25, Supporting Information).

**Figure 7:**
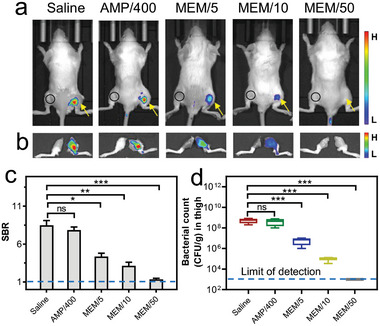
Investigating the therapeutic efficacy of antibiotics in treating infection caused by bla‐expressing resistant bacteria in living mice using **BIN‐3**. Ampicillin (AMP, 400 mg kg^−1^) and meropenem (MEM, 5, 10, and 50 mg kg^−1^), as well as saline (as control), were *i.p*. administered to mice infected by TEM‐1‐encoded *E. coli* 48 h before the *i.v*. administration of **BIN‐3**. a) Representative whole‐body fluorescence images of mice at 5 h post‐administration of **BIN‐3**. b) Fluorescence images of rear thighs at 5 h post‐administration. c) Calculated SBR of infected thighs at 5 h post‐administration. d) Quantified number of bacteria in infected thighs based on a culture‐based assay. *λ*_ex/em_ = 660/710 nm. The black circle in each mouse indicates the position chosen as the background for the calculation of SBR. Data represent mean ± SD (*n* = 3); ns: not significant; * *P* < 0.05; ** *P* < 0.01; *** *P* < 0.001.

In addition to testing mice with acute infection by resistant bacteria, we also examined **BIN‐3** in mice 48 hours post‐bacterial infection in muscle. As shown in **Figures** **7** and S26a (Supporting Information), the *i.v*. administration of **BIN‐3** resulted in a significant fluorescence emission specifically from the infected region (rear right thigh). Moreover, the high sensitivity of **BIN‐3** in the in vivo imaging of bacterial infection was further confirmed by a murine subcutaneous abscesses model^25^ at 48 h post‐infection (Figure S26b, Supporting Information).

### 2.5 Evaluation of Therapeutic Efficacy with **BIN‐3**

As **BIN‐3** proved to be able to detect bla‐expressing bacteria in live mice with high sensitivity in a non‐invasive manner, we wondered whether this reagent is compatible with a rapid assessment of the therapeutic efficacy of antibiotics in living animals with β‐lactamase as the intrinsic reporter resistant bacteria. Obviously, compared to conventional culture‐based approaches, this non‐invasive imaging‐based approach is much more time‐ and cost‐efficient, especially for bacterial pathogens with long passage times, which should facilitate the development of therapeutic agents to combat antibiotic resistance. To investigate this possibility, we used the TEM‐1‐encoded *E. coli* as model‐resistant bacteria. This bacterial strain is highly resistant to narrow‐spectrum antibiotic ampicillin (AMP) with a MIC > 128 µg mL^−1^ but it is susceptible to broad‐spectrum antibiotic meropenem (MEM) with a MIC < 0.03 µg mL^−1^ (Table S3, Supporting Information). We treated infected mice with ampicillin (400 mg kg^−1^), meropenem at various doses (5, 10, and 50 mg kg^−1^), as well as saline (as a control), intraperitoneally one hour after infection.^[^ ^25^ ^]^ The therapeutic efficacy of these antibiotics was then evaluated by *i.v*. administrating **BIN‐3** two days later. The whole‐body images (Figure 7; Figure S27, Supporting Information) show that the mice treated with ampicillin or saline emitted strong fluorescence at the region of infection, indicating that the treatment with ampicillin was largely ineffective against the infection by bla‐positive bacteria in the mice. However, the treatment with meropenem significantly reduced the fluorescence in the thighs of mice, with the degree of reduction being directly proportional to the dose administered. The highest dose of 50 mg kg^−1^ efficiently suppressed the fluorescence emission with an SBR close to 1, indicating the complete purge of bacteria from the infected sites of mice. These results were further confirmed by ex vivo imaging.

To ascertain the accuracy of this imaging‐based approach on the assessment of in vivo therapeutic efficacy, we also used the conventional bacteria culture‐based method and the results were in agreement with those obtained from the imaging‐based assay. In particular, when meropenem was administered at a dosage of 50 mg kg^−1^ to infected mice, all resistant bacteria were eradicated. These results evidently demonstrate that **BIN‐3** provides an accurate assessment of therapeutic efficacy against bacterial infections in vivo. In contrast to traditional bacteria culture‐based methods, which necessitate sacrificing model animals for results, this **BIN‐3**‐based assay is non‐invasive; it reveals therapeutic efficacy without the need for sacrificing model animals and, importantly, allows for continuous monitoring of antibiotic‐treated animals—a crucial aspect for the in vivo evaluation of novel antibiotics.

The combination of a bla inhibitor with β‐lactam antibiotics has proven to be a promising strategy to reverse antibiotic resistance caused by the expression of bla.^[^ ^26^ ^]^ However, typically, a specific inhibitor is effective for reducing a particular type of β‐lactamases. For instance, avibactam is a clinically available bla inhibitor with high inhibition potency against TEM‐1 bla (IC_50 _= 1.136 nm, Figure S28, Supporting Information) but with poor activity against New Delhi metallo β lactamase‐1 (NDM‐1 bla), a type of class B1 β lactamase.^[^ ^26^ ^]^ The combination of avibactam with ampicillin could effectively restore the susceptibility of TEM‐1‐encoded bacteria to ampicillin in the in vitro test. Ebselen, on the other side, is an inhibitor of NDM‐1^[^ ^27^ ^]^ with moderate potency. (IC_50_ = 0.508 µm,^[^ ^28^ ^]^ Figure S29, Supporting Information) but with minimal potency on TEM‐1 bla in the in vitro investigation. Moreover, in contrast to the microplate‐based assay, the assessment of bla inhibitor efficacy in living animals is much more time‐ and cost‐consuming. Taking advantage of the high sensitivity of **BIN‐3** in detecting bla activity in vivo, we wondered whether this compound is useful to investigate the potency of bla inhibitors in living animals in a non‐invasive and continuous manner. Particularly, **BIN‐3** might even allow investigating the efficacy of inhibitors against different types of bacterial β‐lactamases in a single animal.

**Figure 8:**
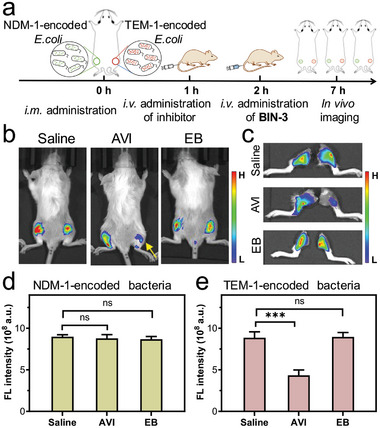
In vivo assessment of β‐lactamase inhibitor efficacy using **BIN‐3**. Avibactam (AVI, an inhibitor for TEM‐1) and ebselen (EB, an inhibitor for NDM‐1), as well as saline (as control), were administered by tail vein to mice with the left rear thigh infected by NDM‐1‐encoded *E. coli* and the right rear thigh infected by TEM‐1‐encoded *E. coli* 1 h before the *i.v*. administration of **BIN‐3**. a) Schematic illustration of experimental schedule. b) Representative whole‐body fluorescence images at 5 h post‐administration of **BIN‐3**. c) Fluorescence images of rear thighs at 5 h post‐administration. d,e) Quantified fluorescence intensity of left rear thigh (infected by NDM‐1‐encoded *E. coli*) and right rear thigh (infected by TEM‐1‐encoded *E. coli*) at 5 h post‐administration. *λ*_ex/em_ = 660/710 nm; data represent mean ± SD (*n* = 3); ns: not significant; * *P* < 0.05; ** *P* < 0.01; *** *P* < 0.001.

To test this possibility, as showed in **Figure** **8**, we infected mice with two types of bla‐expressing *E. coli* in the muscle of the rear thighs (left: NDM‐1‐encoded *E. coli*; right: TEM‐1‐expressing *E. coli*). Both strains of bacteria have proven to be resistant against widely used β‐lactam antibiotic ampicillin with MICs higher than 128 µg mL^−1^ (Table S3, Supporting Information). As a control experiment, the *i.v*. administration of saline followed by **BIN‐3** resulted in massive fluorescent emission from both rear thighs of the mice, indicating that this reagent could be efficiently hydrolyzed by both strains of *E. coli*. To investigate the in vivo inhibition efficacy of avibactam, this compound was intravenously administered followed by the administration of **BIN‐3** 5 h later. The whole body images of mice in Figure 8 and Figure S30 (Supporting Information) showed that the fluorescence emission from the right thighs (infected by TEM‐1‐expressing *E. coli*) was selectively suppressed while the emission from left thighs (infected by NDM‐1‐expressing *E. coli*) were unaffected. The testing of ebselen in living mice as aforementioned resulted in strong fluorescence emission on both thighs, indicating this compound has poor in vivo efficiency to reduce the activity of NDM‐1 bla and TEM‐1 bla. This finding is in line with a previous study, which showed that ebselen, despite its inhibition potency against NDM‐1 bla in the in vitro tests, was less effective in reducing bla activity in living animals,^[^ ^29^ ^]^ likely associated with its poor water solubility. These results support **BIN‐3** as a reliable and convenient tool for evaluating the activity of bla inhibitors in vivo.

## 3 Conclusion

In summary, we have developed a novel hydrophilicity‐switching, self‐immobilizing, near‐infrared fluorogenic probe, **BIN‐3**, for the highly selective and sensitive imaging of infections by β‐lactamase‐expressing resistant bacteria in live mice. This imaging reagent, upon selective hydrolysis by β‐lactamases, could significantly enrich in β‐lactamase‐expressing bacteria in addition to the enhancement in fluorescence intensity as most conventional β‐lactamase probes, taking advantage of the distinctive hydrophilicity‐switching and self‐immobilizing design. Given these features, **BIN‐3** proved to fluorescently label β‐lactamase‐positive bacteria with high selectivity even in the presence of other susceptible bacteria and no cross‐labeling was observed, allowing easy visualization of resistant bacteria in a wash‐free and real‐time manner. More importantly, in the in vivo investigations, the intravenous administration of **BIN‐3** to infected mice led to selective accumulation of activated fluorophore selectively at the site of infection, enabling sensitive and non‐invasive visualization of infections by as low as 2 × 10^6^ CFU of β‐lactamase‐expressing bacteria with a signal‐to‐background ratio of 3.0. Moreover, the potential of this NIR fluorogenic and enriching probe has been further demonstrated in the rapid assessment of the therapeutic efficacy of existing antibiotics at a variety of dosages and the inhibitory potency of β‐lactamase inhibitors in living animals. Compared to conventional culture‐based assays, this non‐invasive approach proved to be more time‐ and cost‐effective and even allows for real‐time monitoring of bacterial dynamics in vivo. This β‐lactamase probe may find great value in the development of novel therapeutic agents fighting against antibiotic resistance or optimization of drug dosing regimens.

## 4 Experimental Section

### Fluorescence Imaging of Bla‐Positive Bacteria

*E. coli* (ATCC 25922), *E. cloacae* (ATCC BAA 1143), TEM‐1‐encoded *E. coli*, mCherry‐encoded *E. coli*, or *E. coli* DH5α were cultured in Luria–Bertani (LB) medium at 37 °C with shaking at 200 rpm. The bacteria were harvested by centrifugation (4000 rpm for 5 min) and washed three times with PBS and were re‐suspended in PBS to a cell density of 1 × 10^10^ CFU mL^−1^ as working suspension. Then 100 µL of the bacterial suspension was incubated with **BIN‐3**/**BIN‐2** for 1 h at 37 °C. For the inhibition experiments, bacteria were pretreated with β‐lactamase inhibitor avibactam (100 µm) for 30 min before incubation with **BIN‐3**/**BIN‐2** for 1 h. The bacteria were spotted on coverslips (Fisherbrand, 24 × 24 mm) and covered by smaller coverslips (20 × 20 mm). The bacteria were imaged using a fluorescence microscope (Leica DMI3000B, Germany) with the Cy7 channel (excitation at 680 nm and emission at 720 nm) without a washing step. For the imaging involving a washing step, the probe‐incubated bacteria were harvested by centrifugation (4000 rpm for 5 min) and washed three times with PBS. The bacteria were re‐suspended in PBS before imaging with a microscope as described above. For the imaging of a mixture of bacteria, a mixture of bla‐negative mCherry‐encoded *E. coli* (5 × 10^8^ CFU) and bla‐positive *E. cloacae* (ATCC BAA 1143, 5 × 10^8^ CFU) in PBS (100 µL) were incubated with **BIN‐3** or **BIN‐2** (20 µm) for 1 h at 37 °C before imaging with a microscope using the mCherry channel (excitation at 580 nm and emission at 620 nm) and the Cy7 channel (excitation at 680 nm and emission at 720 nm).

### Ethics Statement

All animal studies were performed in agreement with the guidelines set by the Institutional Animal Care Use Committee of East China University of Science and Technology (Shanghai, China). All procedures were approved by the Animal Research Bioethics Committee, East China University of Science and Technology (ECUST‐2022‐027).

### Murine Myositis Infection Model

The murine myositis infection model was constructed as a previous report.^[^ ^3a^ ^]^ In brief, BALB/c female mice at 4–6 weeks old were obtained from Shanghai Laboratory Animal Research Center. Mice were housed at 25 °C with free access to food and water. The mice underwent fur removal on their backs through shaving and the application of chemical depilatories before the injection of bacteria. Prior to injection, the bacteria were washed twice with saline and resuspended to the final concentration depending on the strain used. For the thigh myositis infection model, 0.1 mL of a bacterial suspension (5 × 10^8^ CFU per thigh) was injected into the right rear thigh muscle of each mouse. For mice with myositis infection on both rear thigh muscles, bla‐negative and mCherry‐encoded *E. coli* (5 × 10^8^ CFU in 0.1 mL saline) and bla‐encoded *E. coli* (5 × 10^8^ CFU in 0.1 mL saline) were injected to the left and right rear thigh muscle of mouse, respectively.

### Murine Subcutaneous Abscesses Model

The murine subcutaneous abscesses model was constructed as described in the previous report.^[^ ^30^ ^]^ In brief, the mice underwent fur removal on their backs through shaving and the application of chemical depilatories before the injection of bacteria. Bacterial cells were washed twice with sterile saline and resuspended to a final concentration, depending on the strain used. Bla‐encoded *E. coli* (5 × 10^8^ CFU in 50 µL saline) were injected into the dorsum underneath the thin skeletal muscle (*n* = 3).

### Fluorescence Imaging of Infection by Bla‐Positive *E. coli* in Mice

**BIN‐3** or **BIN‐2** (1 µmol kg^−1^) in saline (200 µL) was administered through a tail vein to mice 1 or 48 h after infection in the muscle of the rear right thigh. For inhibition study, bacteria were incubated with avibactam (100 mm) in saline (100 µL) at 37 °C for 2 h before being injection to the muscle of the rear right thigh. The whole‐body fluorescence images were acquired at 1, 2, 3, 4, 6, 8, and 10 h post‐administration using an IVIS Lumina XRMS Series III imaging system. The fluorescence intensities were quantified by the ROI measurement using Living Image Software (PerkinElmer, U.S.A). Each experiment was conducted in five mice.

### In Vivo Assessment of β‐Lactamase Inhibitor Efficacy

To investigate the in vivo efficacy of β‐lactamase inhibitors, 1 × 10^8^ CFU (50 µL) of NDM‐1‐encoded *E. coli* (1 × 10^8^ CFU in 50 µL saline) and TEM‐1‐encoded *E. coli* (1 × 10^8^ CFU in 50 µL saline) were injected into the left and right rear thigh muscle of mice, respectively, 1 h before avibactam or ebselen (10 mg kg^−1^), as well as saline (as control), was administered via tail vein. Probe **BIN‐3** (1 µmol kg^−1^) was administered via the tail vein after 1 h. The whole‐body fluorescence images were collected using the IVIS Lumina XRMS Series III imaging system with excitation and emission wavelengths of 660 and 710 nm, respectively, at 5 h post‐administration of the probe. Each experiment was conducted in three mice.

### In Vivo Assessment of Antibacterial Efficacy

To investigate the therapeutic efficacy of antibiotics in living mice, TEM‐1‐encoded *E. coli* (1 × 10^8^ CFU in 50 µL saline) was injected into the rear right thigh muscle. The treatment of infection with antibiotics followed previously reported protocols.^[^ ^25^, ^31^ ^]^ In brief, at 1 and 25 h post‐infection, ampicillin (400 mg kg^−1^ in 100 µL of saline) was administered intraperitoneally. Meropenem (5/10/50 mg kg^−1^ in 100 µL of saline) was administered intraperitoneally at 1, 9, 17, 25, 33, and 41 h post‐infection. **BIN‐3** (1 µmol kg^−1^) was injected via the tail vein at 49 h post‐infection and the whole‐body fluorescence images were collected at 5 h post‐administration of the probe as described above. These mice were euthanized by cervical dislocation. The right rear thighs were aseptically removed, homogenized, serially diluted, and plated on plate LB to count bacterial numbers after incubated 12 h at 37 °C. Each experiment was conducted in three mice.

### Statistical Analysis

The graphs were obtained by using Graph Pad Prism 7 software. Image J software was used to analyze fluorescent images and convert a 16‐bit grayscale image to pseudo color. The in vivo fluorescence images were acquired using an IVIS Lumina XRMS Series III imaging system. Quantitative data was expressed as mean ± standard deviation (SD). A *T*‐test was used to compare whether there was a difference between the two groups, one‐way ANOVA test was used to evaluate the difference among three or more groups. ns was defined as not significant, *, **, and*** represent *P* < 0.05, *P* < 0.01, *P* < 0.001, respectively.

## Conflict of Interest

The authors declare no conflict of interest.

## Author Contributions

F.C. and Y.L. contributed equally to this work.

## Supporting information



Supporting Information

## Data Availability

The data that support the findings of this study are available in the supplementary material of this article.
