# Supplementary material for: Highly Sensitive In Vivo Imaging of Bacterial Infections with a Hydrophilicity‐Switching, Self‐Immobilizing, Near‐Infrared Fluorogenic β‐Lactamase Probe Enriched within Bacteria
Source: Adv Sci (Weinh). 2024 Dec 12;12(5):2408559. doi: 10.1002/advs.202408559 (PMC11791975; doi:10.1002/advs.202408559)

ADVANCED SCIENCE
Open Access

Supporting Information

for *Adv. Sci.*, DOI 10.1002/advs.202408559

Highly Sensitive In Vivo Imaging of Bacterial Infections with a Hydrophilicity-Switching, Self-Immobilizing, Near-Infrared Fluorogenic β-Lactamase Probe Enriched within Bacteria

*Fangfang Chen, Yuyao Li, Yan Peng, Yifan Zhu, Gao He, Zhengwei Zhang and Hexin Xie**

Supplementary Information

Highly Sensitive In Vivo Imaging of Bacterial Infections With a Hydrophilicity-Switching, Self-Immobilizing, Near-Infrared Fluorogenic β-Lactamase Probe Enriched within Bacteria

Fangfang Chen,#,[a] Yuyao Li,#,[a] Yan Peng,[a] Yifan Zhu,[a] Gao He,[a] Zhengwei Zhang,[b] and Hexin Xie*,[a]

[a] State Key Laboratory of Bioreactor Engineering, Shanghai Key Laboratory of New Drug Design, Frontiers Science Center for Materiobiology and Dynamic Chemistry, Shanghai Frontier Science Research Base of Optogenetic Techniques for Cell Metabolism, School of Pharmacy, East China University of Science and Technology, Shanghai 200237 China. E-mail: xiehexin@ecust.edu.cn

[b] Department of Nuclear Medicine & PET Center, Huashan Hospital, Fudan University, Shanghai 200235 China

# These authors contributed equally

Contents

Scheme S1 ... 4
Scheme S2 ... 4
Scheme S3 ... 4
Figure S1 ... 5
Figure S2 ... 5
Figure S3 ... 6
Figure S4 ... 6
Figure S5 ... 7
Figure S6 ... 7
Figure S7 ... 8
Figure S8 ... 8
Table S1 ... 9
Table S2 ... 9
Table S3. ... 9
Figure S9 ... 10
Figure S10 ... 11
Figure S11 ... 11
Figure S12 ... 12
Figure S13 ... 12
Figure S14 ... 13
Figure S15 ... 14
Figure S16 ... 15
Figure S17 ... 16
Figure S18 ... 17
Figure S19 ... 17
Figure S20 ... 18
Figure S21 ... 18
Figure S22 ... 19
Figure S23 ... 20
Figure S24 ... 21
Figure S25 ... 22
Figure S26 ... 22
Figure S27 ... 23
Figure S28 ... 24
Figure S29 ... 25
Figure S30 ... 26
General Information ... 27
Synthesis and Characterization ... 28
Enzyme-Based Investigations ... 35
  General Procedure for Enzymatic Assays ... 35
  Specificity Test of **BIN-3** ... 35
  In-gel Fluorescence Imaging of Protein upon Incubation with **BIN-3** ... 35

Assay for IC_{50} of β-Lactamase Inhibitor 36
Bacteria-Related Experiments 36
Construction of the β-Lactamase-Expressing Plasmids and the Transformation to *E. coli* 36
Minimum Inhibitory Concentration (MIC) Assay 37
Bacteria Viability Assay 37
Determination of Concentration of HD Dye in Bacteria Lysates and Incubation Medium 37
Bacteria Inhibition Assay 38
Checkboard Broth Microdilution Assay 38
Cell-Related Experiments 38
Cell Culture Conditions 38
Cell Viability Assays 38
Hemolysis Assays 39
Animal-Related Experiments 39
In Vivo Cytotoxicity of Probe 39
The Biological Metabolism of Probe 39
References 41
^{1}H and ^{13}C NMR Spectra 42
HRMS Spectra 50
HPLC Traces 53

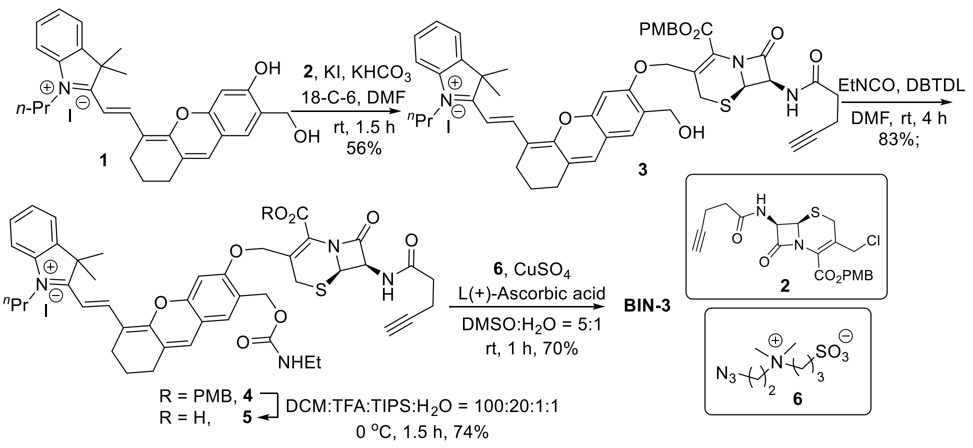

Scheme S1. Synthesis of β-lactamase-activatable NIR probe **BIN-3**. DBTDL: dibutyltin dilaurate

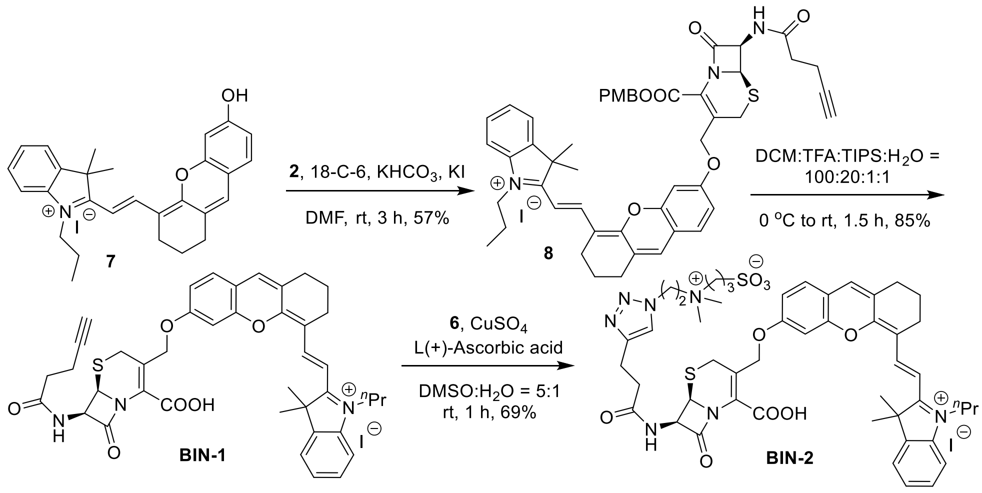

Scheme S2. Synthesis of β-lactamase-activatable NIR probes **BIN-1** and **BIN-2**.

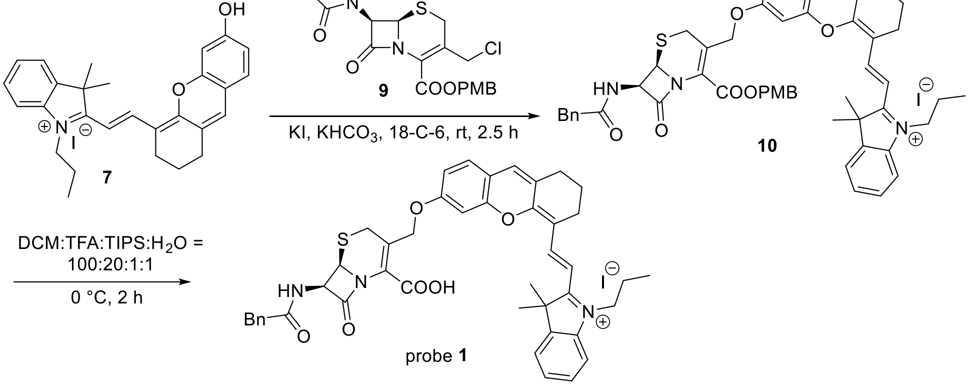

Scheme S3. Synthesis of β-lactamase-activatable NIR probe **1**.

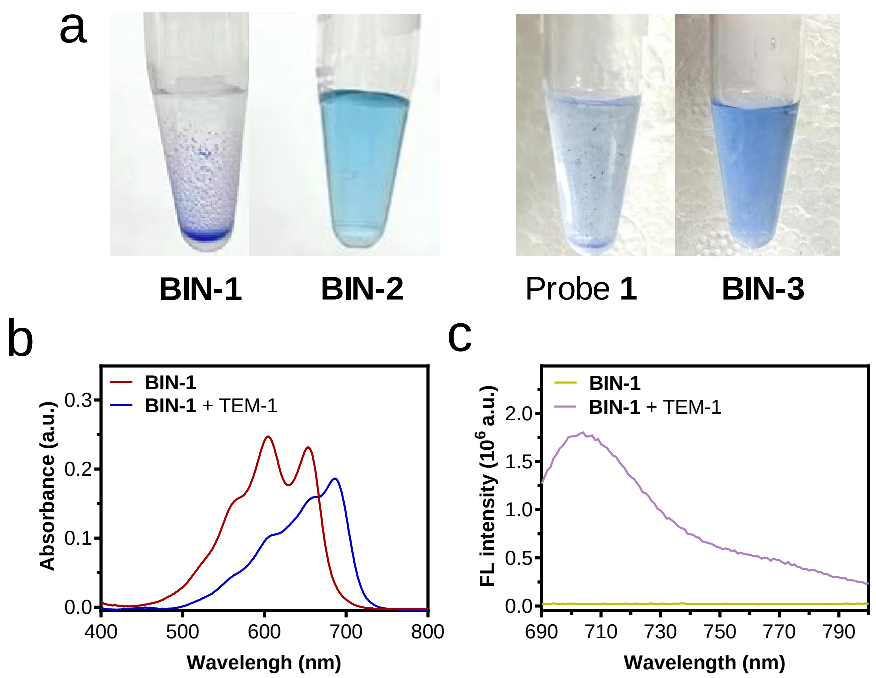

Figure S1. *In vitro* characterization of β-lactamase probe **BIN-1**. a) Photographs of **BIN-1**, **BIN-2**, probe **1** and **BIN-3** (5 μM) in PBS (pH 7.4). b) UV-Vis absorption spectra of **BIN-1** (5 μM) in PBS (pH 7.4) with 10% DMSO before and after incubation with TEM-1 (100 nM) at 37 °C for 15 min. c) Fluorescence spectra of **BIN-1** (5 μM) in PBS (pH 7.4) with 10% DMSO before and after incubation with TEM-1 (100 nM) at 37 °C for 15 min. $\lambda_{ex/em}$ = 687/714 nm.

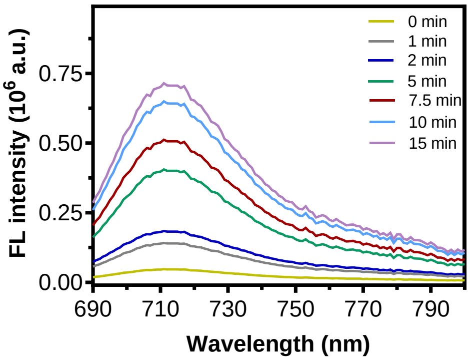

Figure S2. Fluorescence spectra of **BIN-3** (5 μM) in PBS (pH 7.4) after incubation with TEM-1 (100 nM) at 37 °C for 0 to 15 min. $\lambda_{ex/em}$ = 687/714 nm.

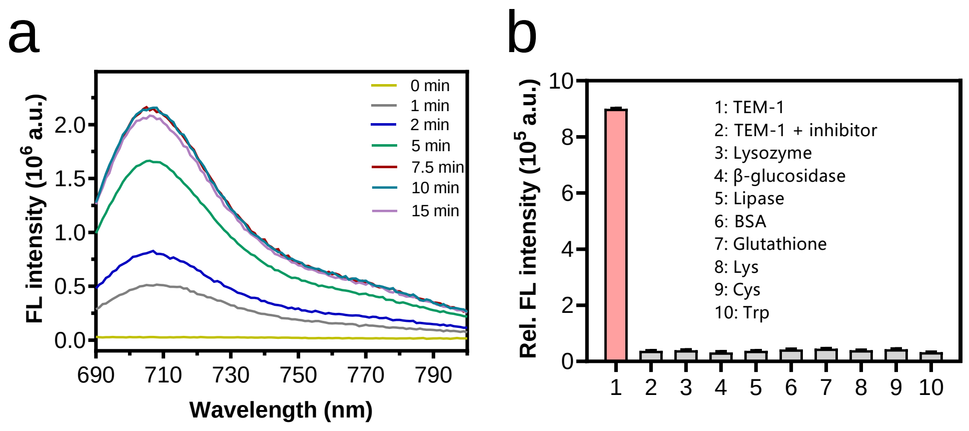

Figure S3. *In vitro* characterization of β-lactamase probe **BIN-2**. a) Fluorescence spectra of **BIN-2** (5 μM) in PBS (pH 7.4) after incubation with TEM-1 (100 nM) at 37 °C for 0 to 15 min. b) Fluorescence response of **BIN-2** (10 μM) to various analytes in PBS. 1: TEM-1 (10 nM); 2: TEM-1 (10 nM) + inhibitor (Avibactam, 100 μM) 3: Lysozyme (2 U/mL); 4: β-glucosidase (5 μM); 5: Lipase (2 U/mL); 6: BSA (10 μM); 7: Glutathione (2 μM); 8: Lys (1 mM); 9: Cys (1 mM); 10: Trp (1 mM). $\lambda_{ex/em}$ = 687/714 nm. Error bars mean ± SD (n = 3 technical replicates).

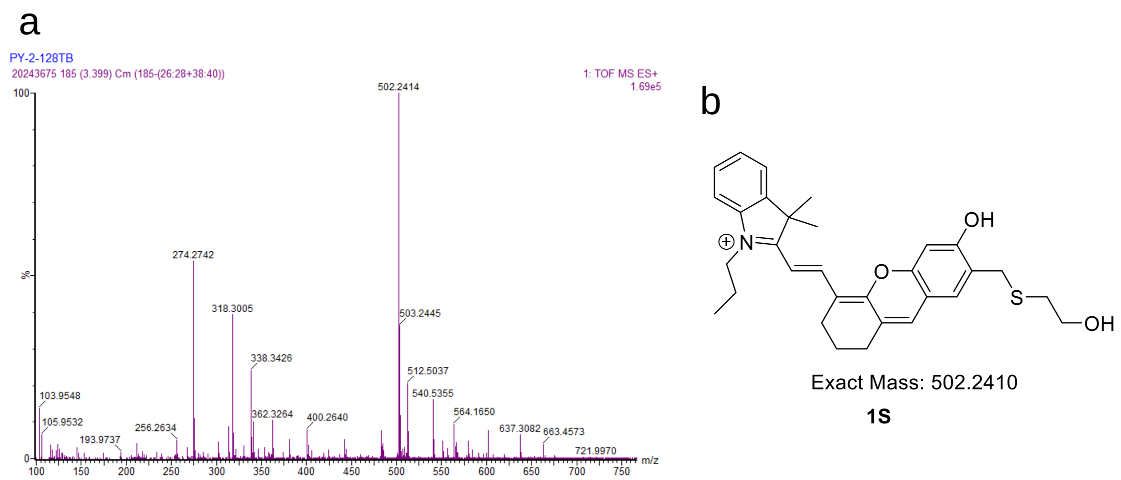

Figure S4. Analysis of **BIN-3** upon incubation with TEM-1 and β-ME. a) HR-MS (ESI) spectrum of the main component detected from **BIN-3** upon incubation with TEM-1 and β-ME (the peak with a retention time of 26.5 min at Fig. 2e, yellow line). b) Chemical structure of compound **1S**.

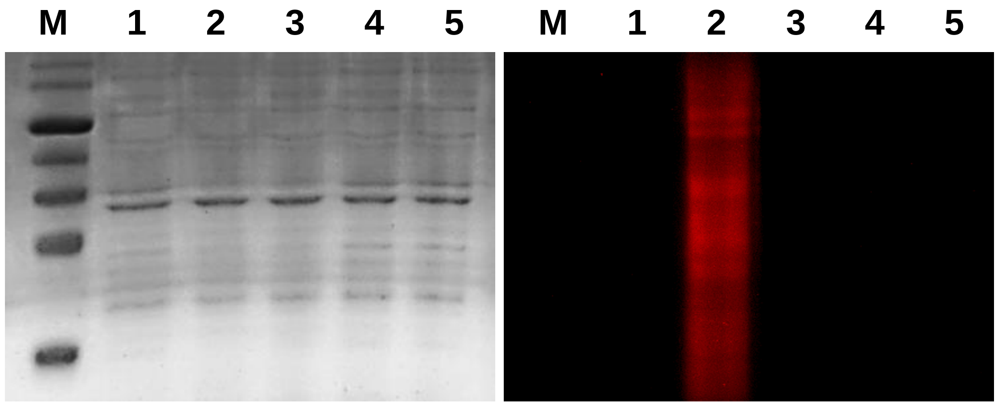

Figure S5. Coomassie blue staining (left) and in-gel fluorescence imaging (right) of bacterial lysate of upon incubation with probes. Lysate of bla-expressing *E. cloacae* or bla-negative *E. coli* was incubated with indicated probe in PBS in the presence or the absence of bla inhibitor at 37 °C for 2 h before SDS-PAGE gel analysis. M: protein marker; 1: lysate of bla-positive *E. cloacae*; 2: lysate of bla-positive *E. cloacae* + **BIN-3**; 3: lysate of bla-positive *E. cloacae* + **BIN-3** + inhibitor; 4: lysate of bla-positive *E. cloacae* + **BIN-2**; 5: lysate of bla-negative *E. coli* + **BIN-3**; $\lambda_{ex/em}$ = 685/720 nm; inhibitor = avibactam.

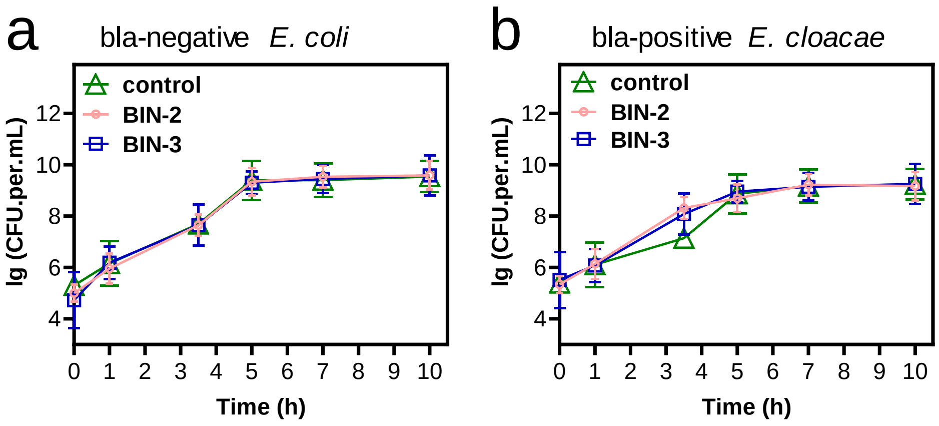

Figure S6. Test of bacterial growth in the presence of **BIN-3** or **BIN-2**. Bla-negative *E. coli* ATCC 25922 (a) and bla-positive *E. cloacae* ATCC BAA-1143 (b) were incubated in LB at 37 °C in the presence of **BIN-3** or **BIN-2** (50 μM) for 10 h. At 1, 3.5, 5, 7 and 10 h, an aliquot of culture medium was taken to count the viable bacterial number by spotting on LB agar plates. Error bars mean ± SD (n = 3 technical replicates).

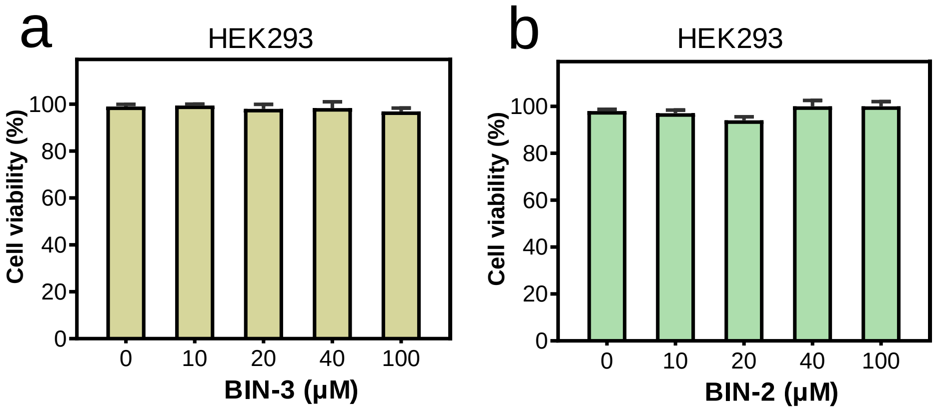

Figure S7. Cytotoxicity of **BIN-3** (a) or **BIN-2** (b) to HEK293 cells. HEK293 cells were incubated with **BIN-3** or **BIN-2** at 0, 10, 20, 40, 100 μM for 24 h and the cell viability was determined by CCK-8 assay. Error bars mean ± SD (*n* = 3 technical replicates).

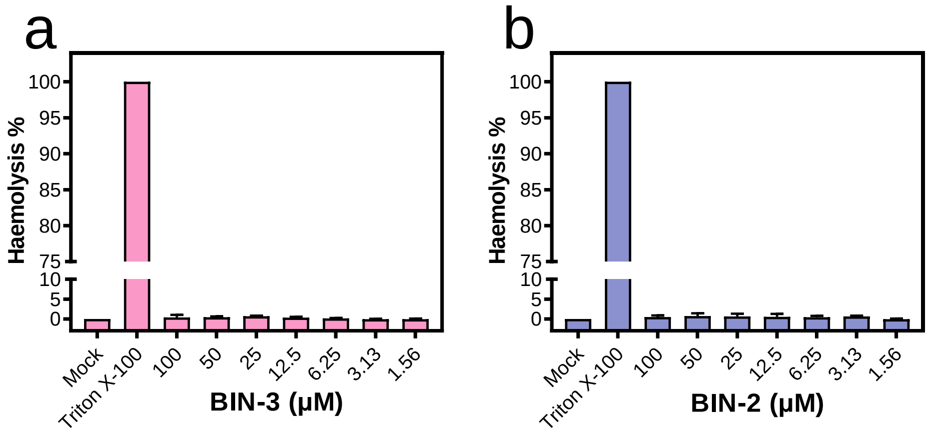

Figure S8. Hemolytic toxicity of **BIN-3** (a) or **BIN-2** (b). The hemolysis assay was performed by incubating the probes with mouse red blood cells (RBCs) and measuring the OD_{540} of the resulting supernatant. Error bars mean ± SD (*n* = 3 technical replicates).

Table S1. Plasmids used for mCherry and β-lactamase production

**Plasmids**	**characteristics**	**sources**
pBBR1MCS2-Tac-mCherry	pBBR1 vector carrying mCherry	In house
pBBR1MCS2-Tac-TEM-1	pBBR1 vector carrying $bla_{\mathrm{TEM\text{-}1}}$	In house
pUC19-pre-NDM-1	pUC19 vector carrying $bla_{\mathrm{NDM\text{-}1}}$	In house
pBBR1MCS2	plasmid for cloning expression	In house
pUC19	plasmid for cloning expression	In house

Table S2. Primers used in this study

**Primer**	**Sequences**
pUC19-NDM-1-F	5′-GCATGCCTGCAGGTCGACTCACCTCATGTTTGAATTCGCC-3′
pUC19-NDM-1-R	5′-GCTCGGTACCCGGGGATCCTCTCTGTCACATCGAAATCGC-3′
pBBR1-TEM-1-F	5′-TCACACAGGAAACAGTATTCATGAGTATTCAACATTTTCG-3′
pBBR1-TEM-1-R	5′-GTGGCAGCAGCCTAGGTTAATTACCAATGCTTAATCAGTG-3′
pBBR1-F	5′-TTAACCTAGGCTGCTGCCAC-3′
pBBR1-R	5′-GAATACTGTTTCCTGTGTGA-3′

Table S3. MICs (μg/mL) of β-lactam antibiotics against β-lactamase-producing bacteria DH5α-pBBR1-TEM-1 and DH5α-pUC19-NDM-1.

	E. coli (ATCC 25922)	*E. coli* DH5α	*E. coli* DH5α-pBBR1-TEM-1	*E. coli* DH5α-pUC19-NDM-1
Ampicillin	4	2	> 128	> 128
Oxacillin	2	0.5	4	> 128
Cefazolin	1	0.25	32	> 128
Meropenem	< 0.031	< 0.031	< 0.031	32

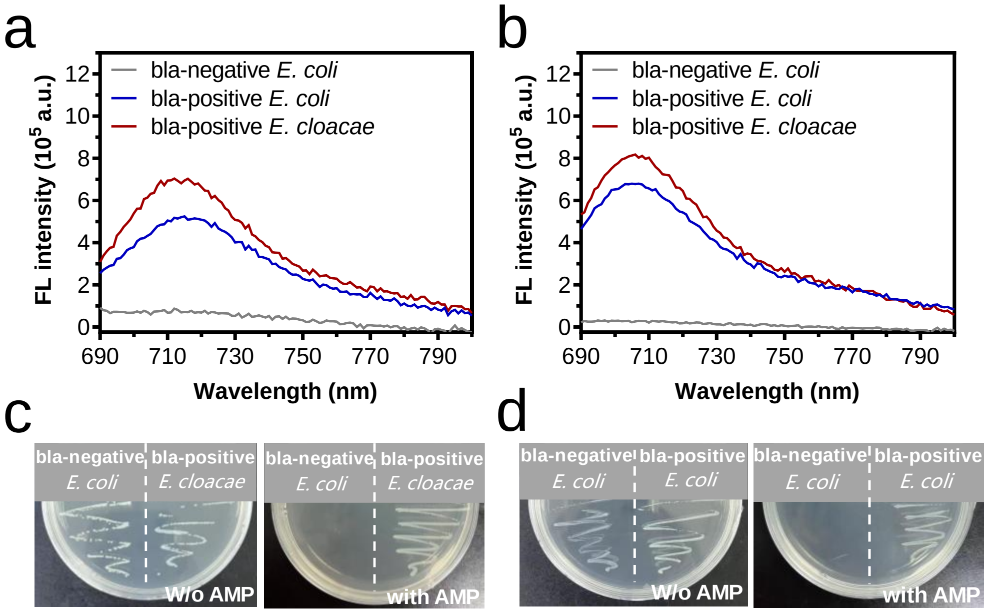

Figure S9. Fluorescence spectra of probe in the presence of bacteria. **BIN-3** (a) or **BIN-2** (b) (5 μM) in PBS (pH 7.4) upon incubation with bla-negative *E. coli* (gray), bla-positive *E. coli* (DH5α-TEM-1, blue) or bla-positive *E. cloacae* (ATCC BAA-1143, red) at 37 °C for 1 h. c) d) Images of resistant and susceptible bacteria in LB plates without (left) or with (right) 100 μg/mL of AMP (Ampicillin).

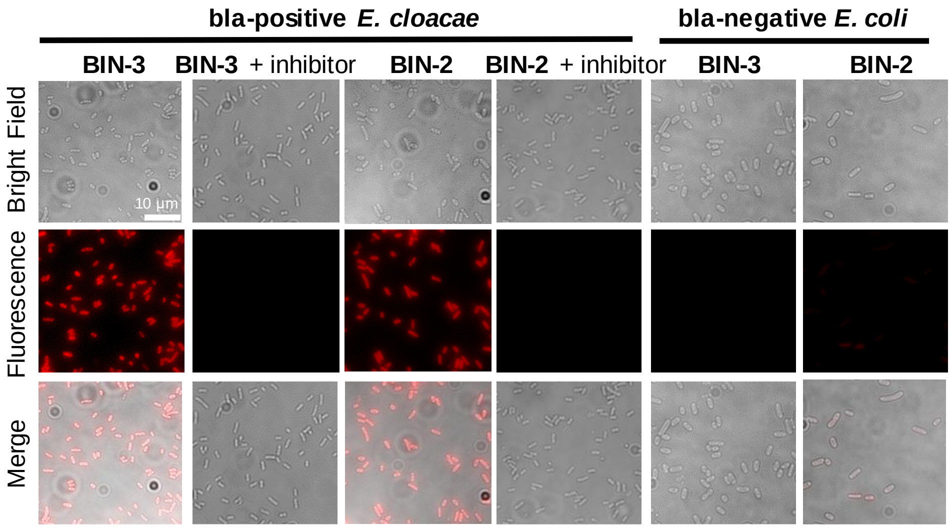

Figure S10. Fluorescence microscope images of bla-expressing *E. cloacae* and with **BIN-3** or **BIN-2**. *E. cloacae* (ATCC BAA-1143) or bla-negative *E. coli* (ATCC 25922) was incubated with **BIN-3** or **BIN-2** (50 µM) in the absence or presence of β-lactamase inhibitor, avibactam (100 µM) for 2 h before wash-free imaging.

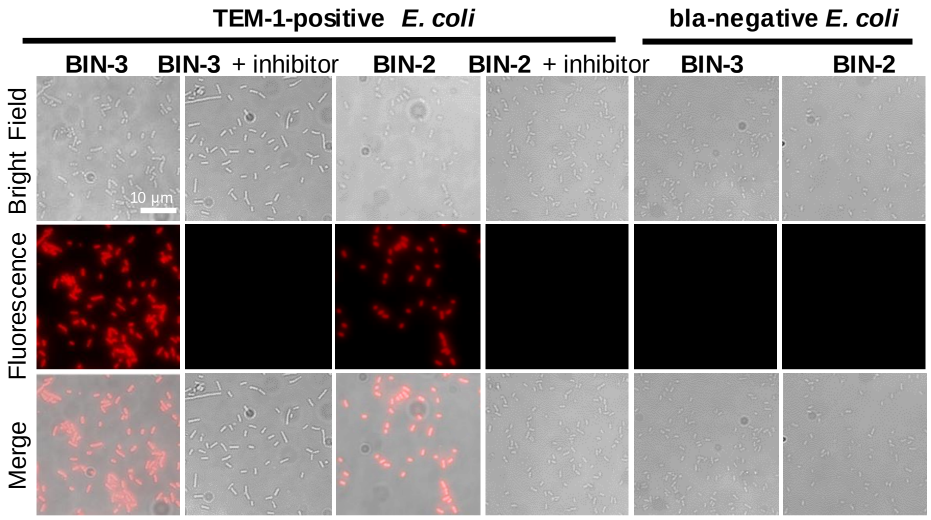

Figure S11. Fluorescence microscope images of TEM-1-expressing *E. coli* using **BIN-3** or **BIN-2**. Bla-encoded *E. coli* (DH5α-TEM-1) or bla-negative *E. coli* (DH5α) was incubated with **BIN-3** or **BIN-2** (50 µM) in the absence or presence of inhibitor (Avibactam, 100 µM) for 2 h before wash-free imaging.

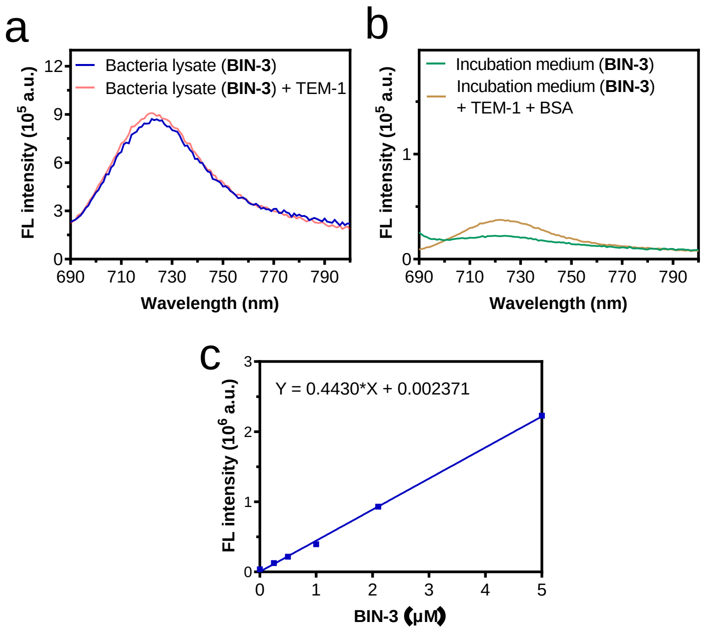

Figure S12. Fluorescence spectra of bla-positive *E. cloacae* (ATCC BAA-1143) lysate (a) and incubation medium (b) after incubation with **BIN-3**. c) Linear fitting curve of fluorescence intensity with the concentration of **BIN-3** after hydrolysis by TEM-1. Error bars mean ± SD (n = 3 technical replicates).

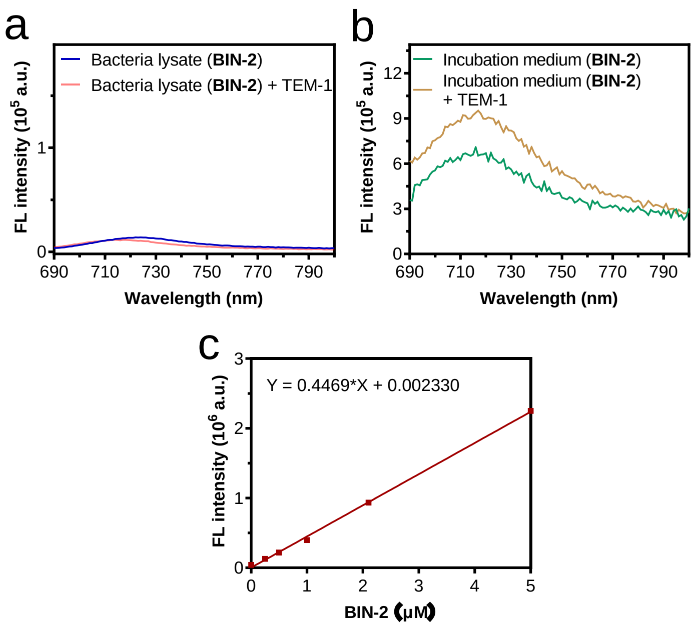

Figure S13. Fluorescence spectra of bla-positive *E. cloacae* (ATCC BAA-1143) lysate (a) and incubation medium (b) after incubation with **BIN-2**. c) Linear fitting curve of fluorescence intensity with the concentration of **BIN-2** after hydrolysis by TEM-1. Error bars mean ± SD (n = 3 technical replicates).

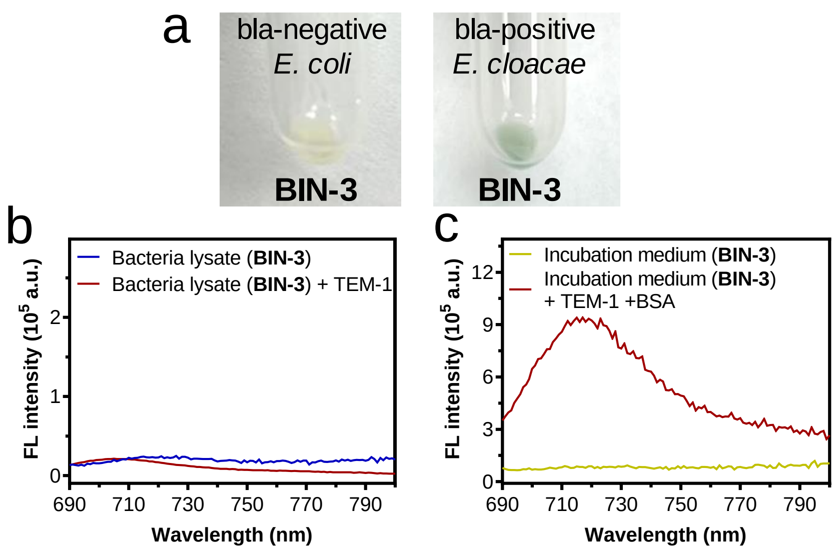

Figure S14. a) Photographs of pellets of bla-negative *E. coli* (ATCC 25922) and bla-positive *E. cloacae* (ATCC BAA-1143) upon incubation with **BIN-3**. b) c) Fluorescence spectra of bla-negative *E. coli* lysate (b) and incubation medium (c) after incubation of **BIN-3**.

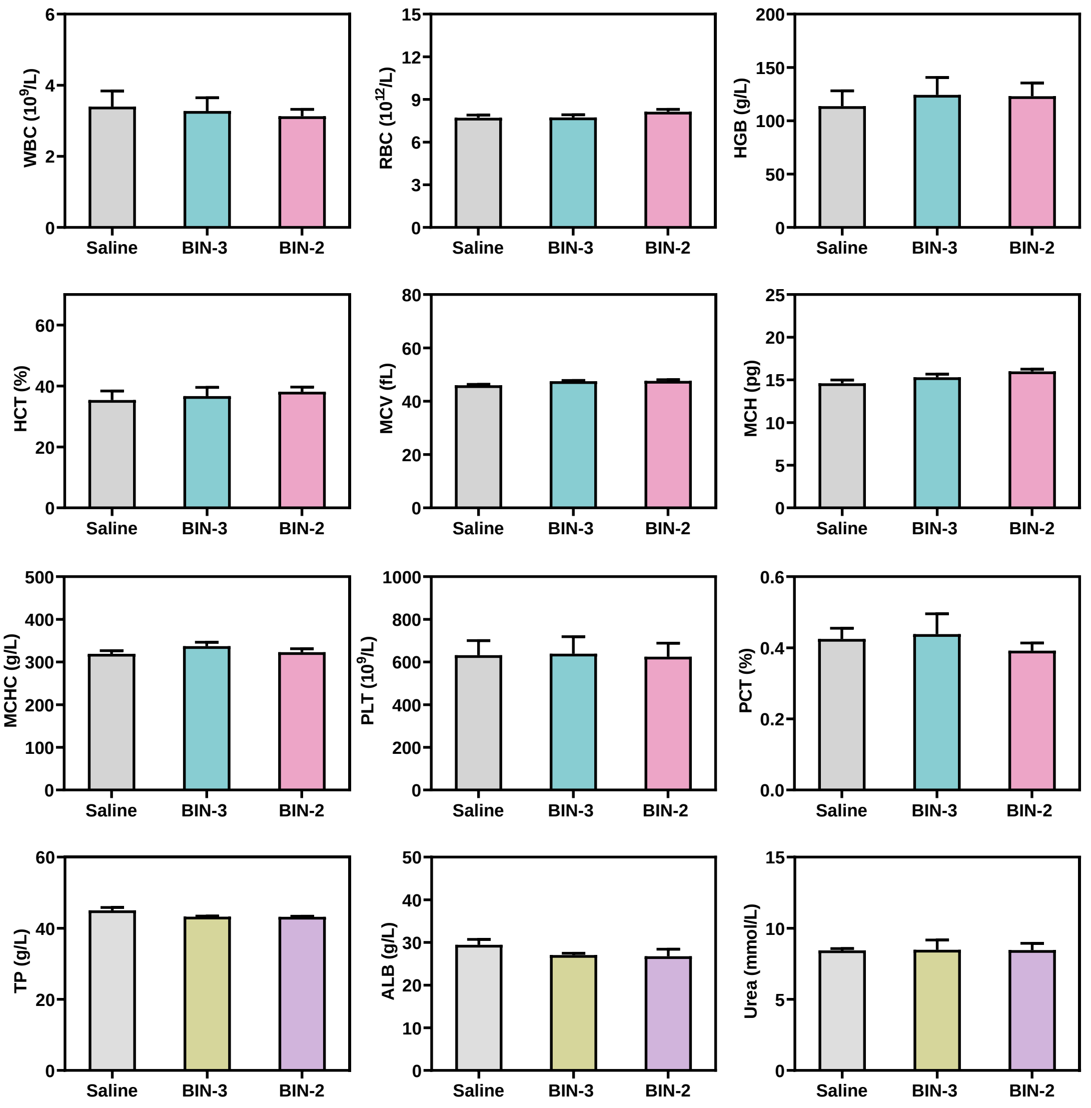

Figure S15. Blood biochemical indexes of mice upon treatment of probe **BIN-3** (1 μmol/kg) or **BIN-2** (1 μmol/kg). WBC: white blood cells; RBC: red blood cells; HGB: hemoglobin; HCT: hematocrit; MCV: mean corpuscular volume; MCH: mean corpuscular hemoglobin; MCHC: mean corpuscular hemoglobin concentration; PLT: platelet count; PCT: Procalcitonin; TP: total protein; ALB: albumin. Error bars mean ± SD (n = 3 technical replicates).

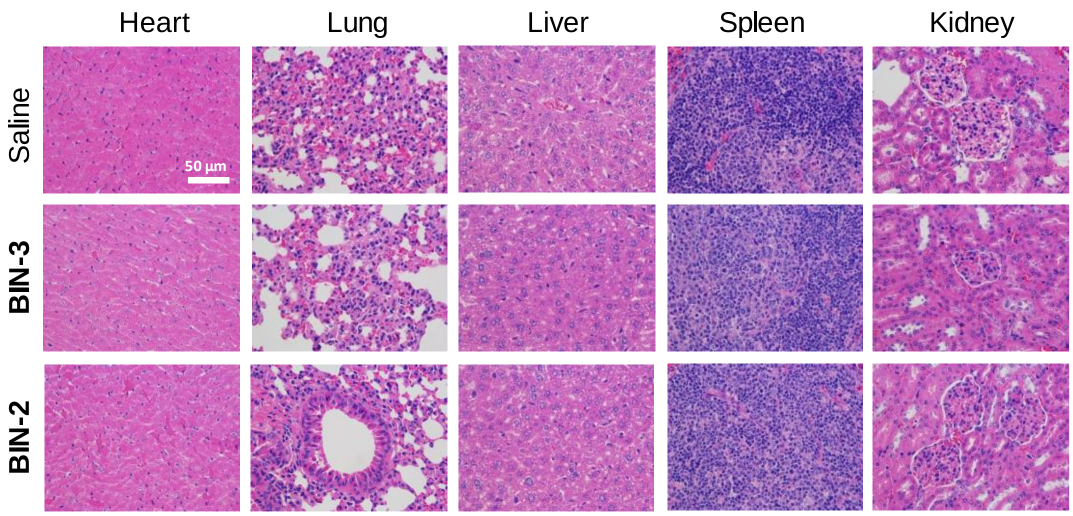

Figure S16. Representative hematoxylin-eosin staining (H&E staining) images of the organs resected from the normal mice after *i.v.* injection of **BIN-3** (1 μmol/kg), **BIN-2** (1 μmol/kg) or saline for 24 h. Scale bar = 50 μm.

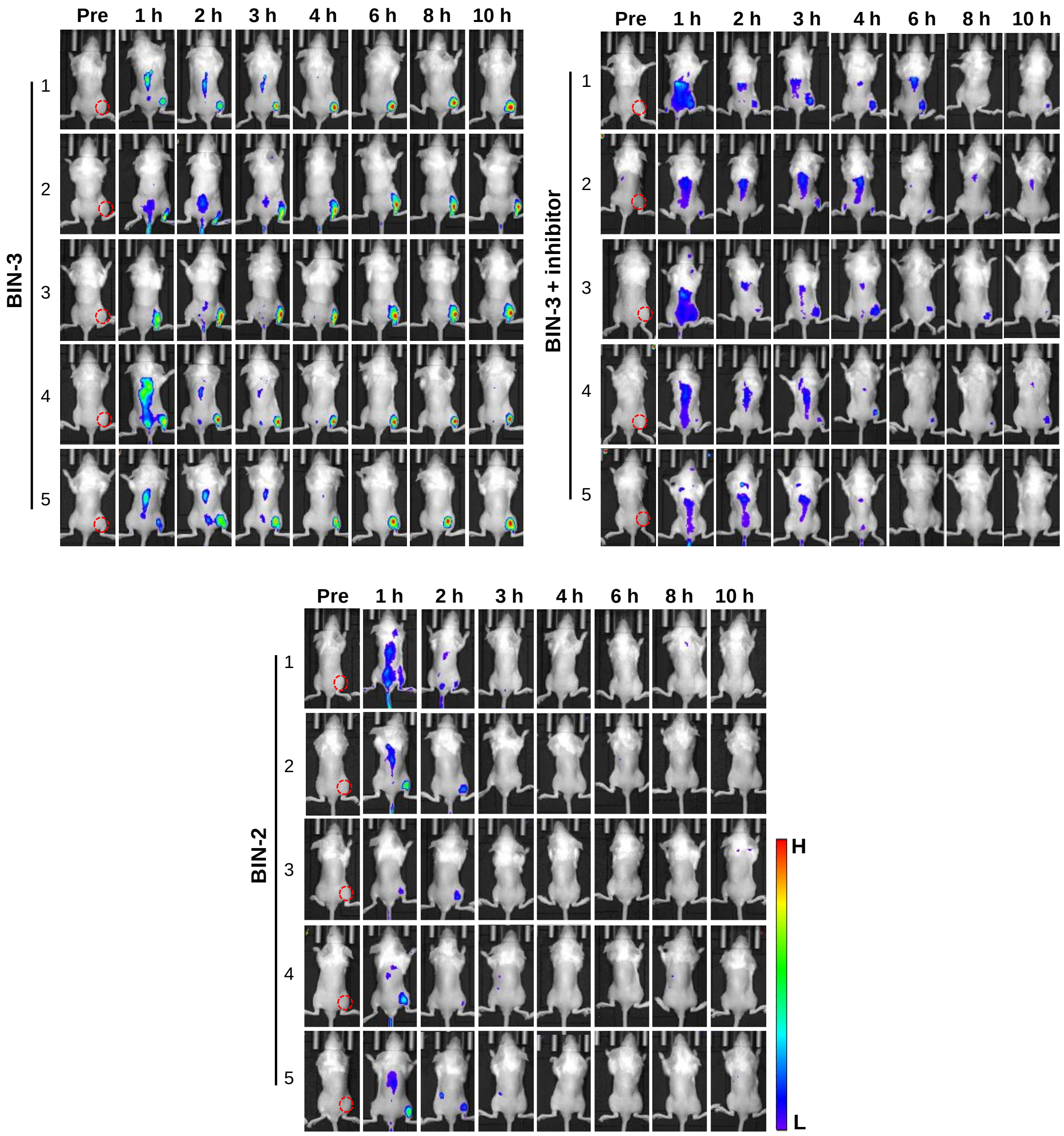

Figure S17. *In vivo* imaging of infection by bla-positive *E. coli* using **BIN-3** or **BIN-2** (1 μmol/kg). For the imaging with inhibitor, bacteria were pretreated with SBL inhibitor avibactam before being injected to mouse. Whole-body fluorescence images of mouse with infection by bla-positive *E. coli* at the muscle of right rear thigh after *i.v.* administration of probes. Red circles point the infected location in mice. $\lambda_{ex/em}$ = 660/710 nm.

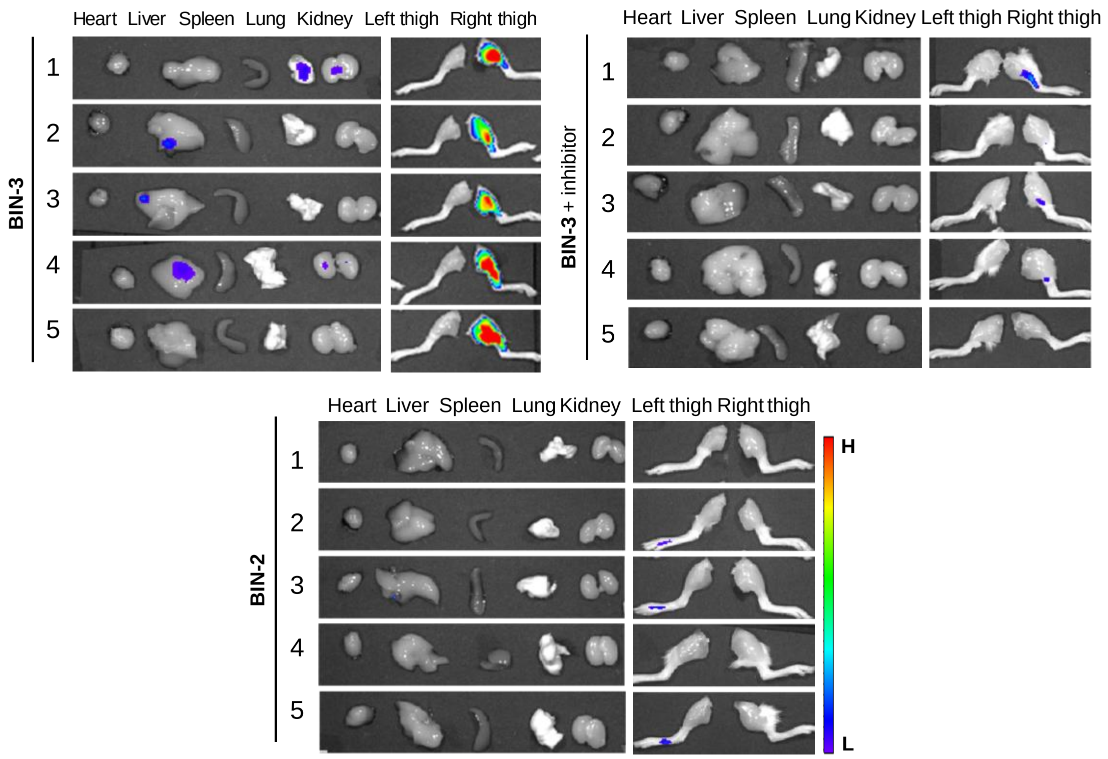

Figure S18. Fluorescence images of thighs and main organs from mice at 10 h after *i.v.* injection of **BIN-3/BIN-2**. For the imaging with inhibitor, bacteria were pretreated with avibactam before being injected to mouse.

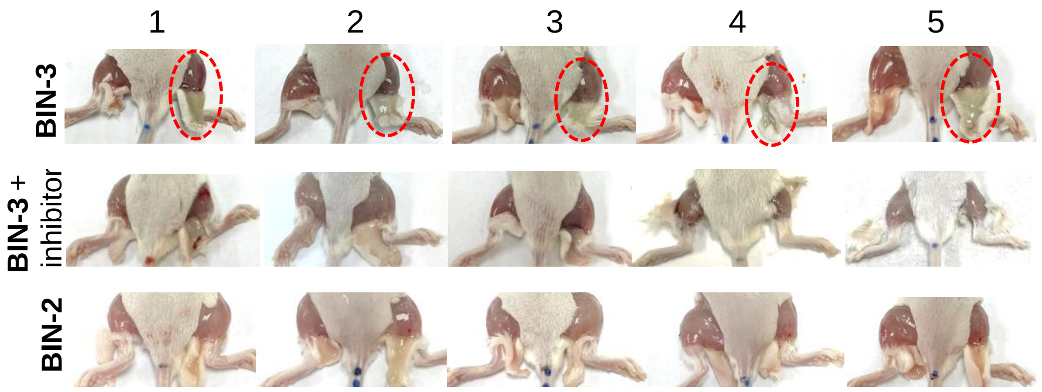

Figure S19. Photograph of thighs from mice after removing skins at 10 h post-administration of **BIN-3** or **BIN-2**. For the imaging with inhibitor, bacteria were pretreated with avibactam before being injected to mouse.

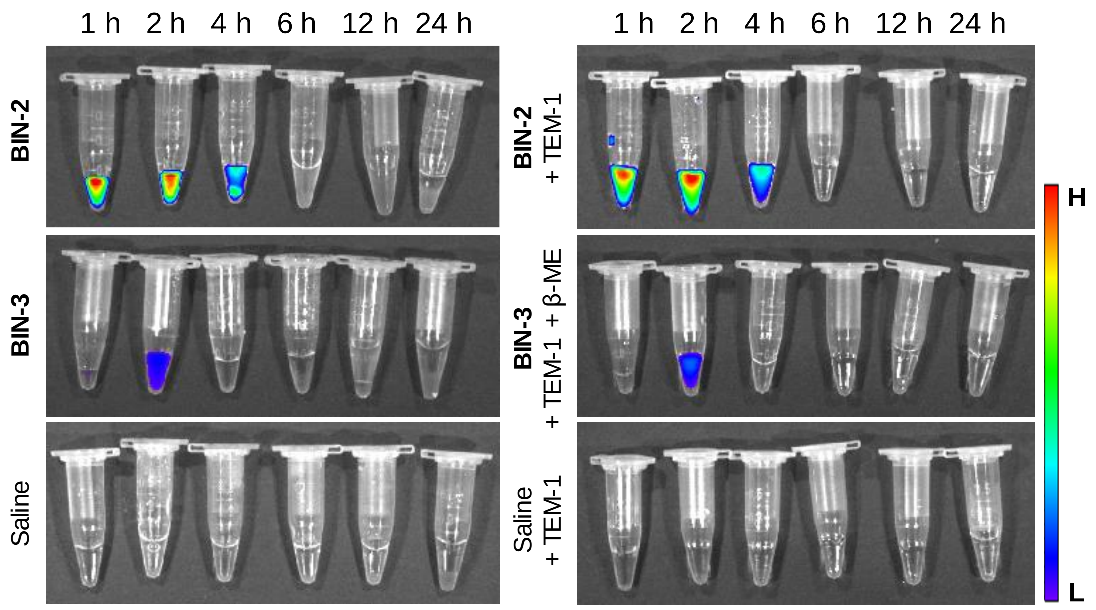

Figure S20. Fluorescence images of urine before (left) and after (right) incubation with TEM-1 (100 nM) or β-mercaptoethanol (10 mM) at 37 ºC for 30 min from mice with myositis infection at different times after *i.v.* injection of **BIN-3**, **BIN-2** or saline.

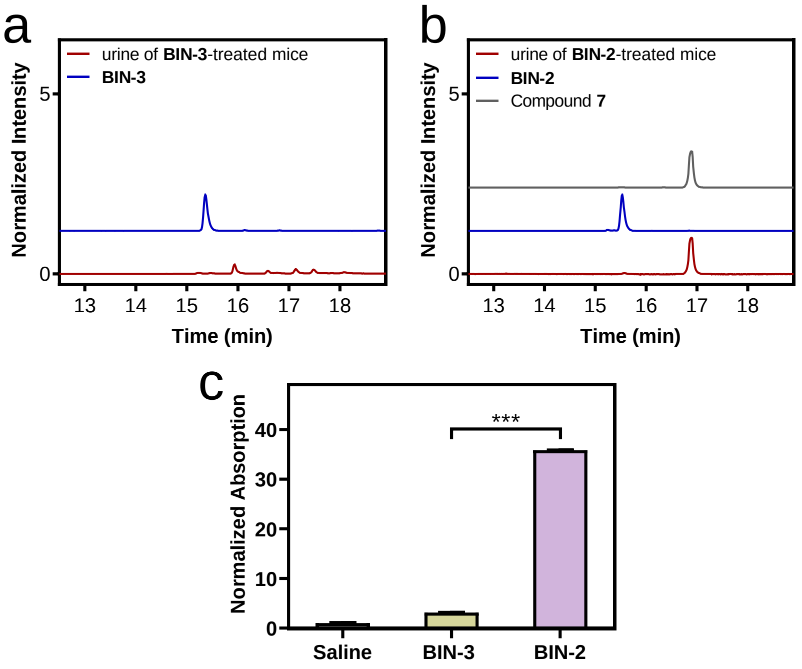

Figure S21. a) b) Representative HPLC traces of urine from **BIN-3**- or **BIN-2**-treated mice at 2 h post *i.v.* injection. c) Absorption (600 nm) of urine from probe-injected mice at 2 h post *i.v.* injection. Error bars mean ± SD (ns: not significant; * $P < 0.05$; ** $P < 0.01$; *** $P < 0.001$, n = 3 technical replicates).

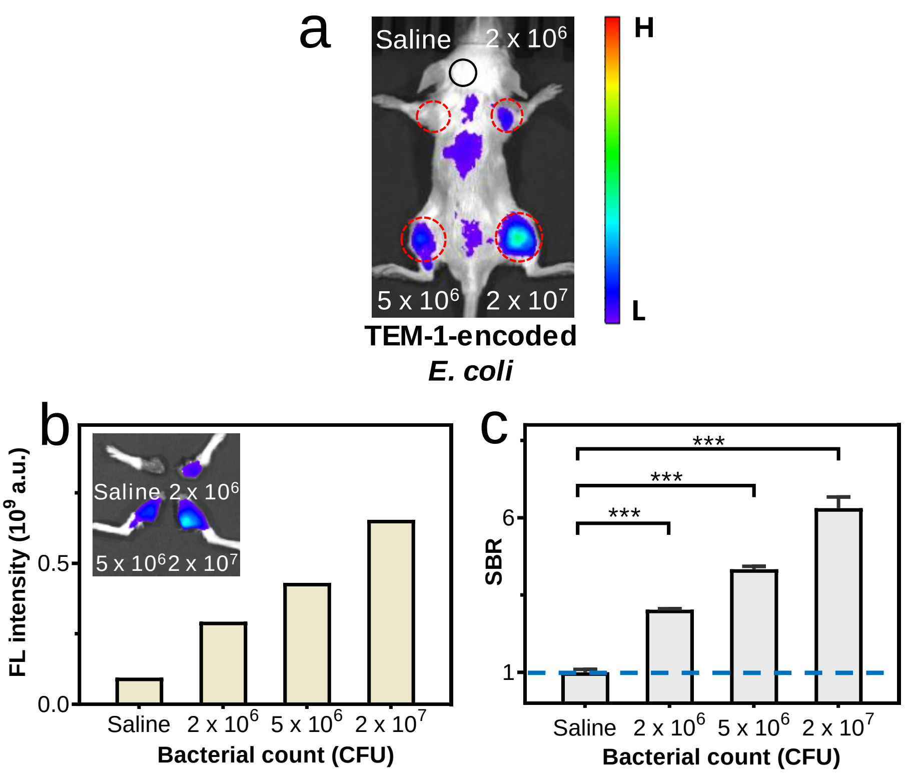

Figure S22. a) Whole-body images of mice with infection at muscle of thighs by various number of bla-encoded *E. coli* using **BIN-3**. b) Quantified fluorescence intensity of infected thighs in (a). Fluorescence images of thighs are shown as insets. c) Calculated SBR of site of infection. The black circle in each mouse indicates the position chosen as background for the calculation of SBR. $\lambda_{ex/em}$ = 660/710 nm. Error bars mean ± SD (ns: not significant; * $P < 0.05$; ** $P < 0.01$; *** $P < 0.001$, n = 3 technical replicates).

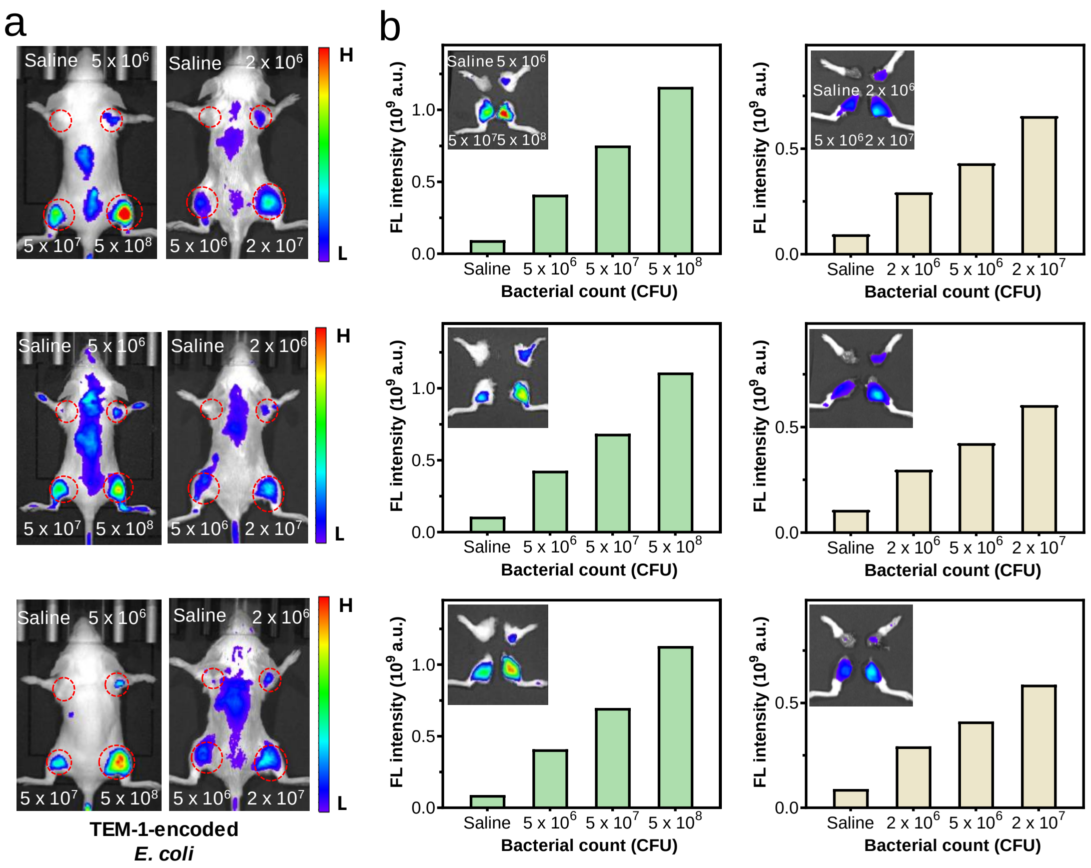

Figure S23. a) Whole-body images of mice with infection at muscle of thighs by various number of bla-encoded *E. coli* using **BIN-3**. b) Quantified fluorescence intensity of infected thighs in (a). Fluorescence images of thighs are shown as insets. $\lambda_{ex/em}$ = 660/710 nm.

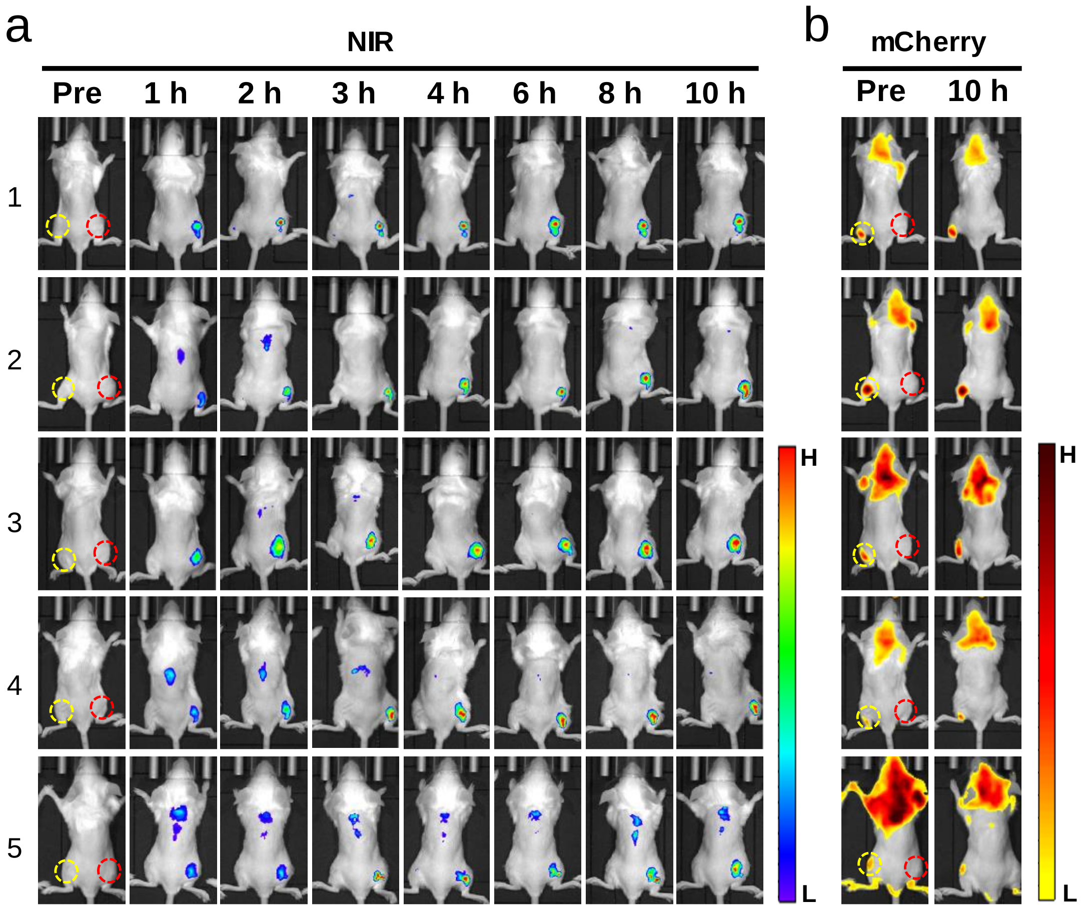

Figure S24. Whole-body images of mice with infections in the muscle by mCherry-encoded and bla-negative *E. coli* (left thigh) and TEM-1 bla-encoded *E. coli* (right thigh) at 10 h post-administration of **BIN-3**. mCherry channel: $\lambda_{ex/em}$ = 580/620 nm; NIR channel $\lambda_{ex/em}$ = 660/710 nm.

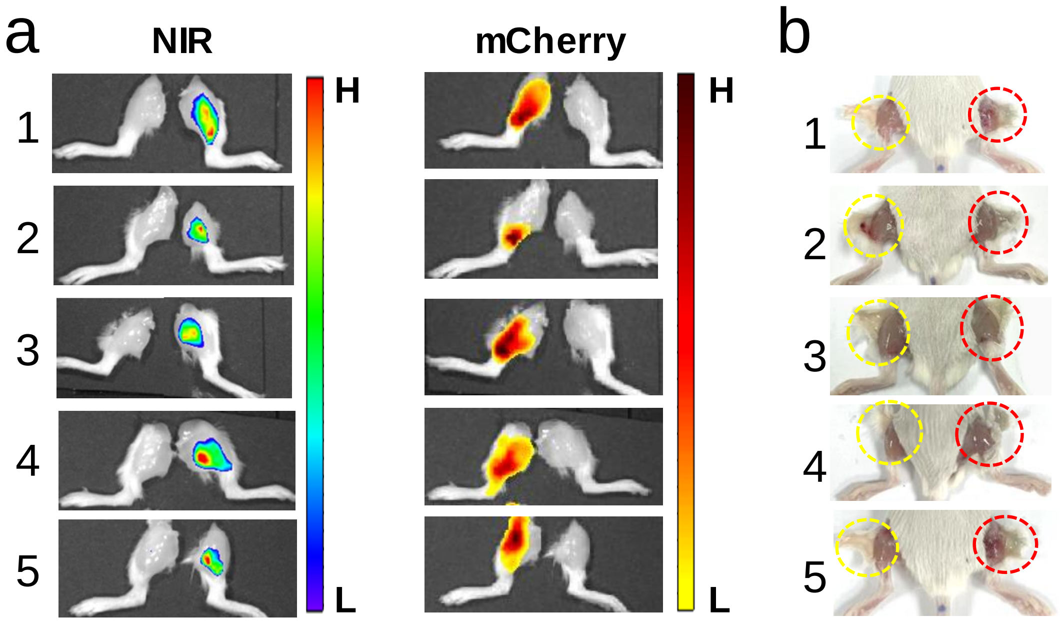

Figure S25. Fluorescence images and photographs of infected rear thighs. a) Fluorescence images of rear thighs using the mCherry and the NIR channel at 10 h post-administration. b) Photographs of infected thighs at 10 h post-injection.

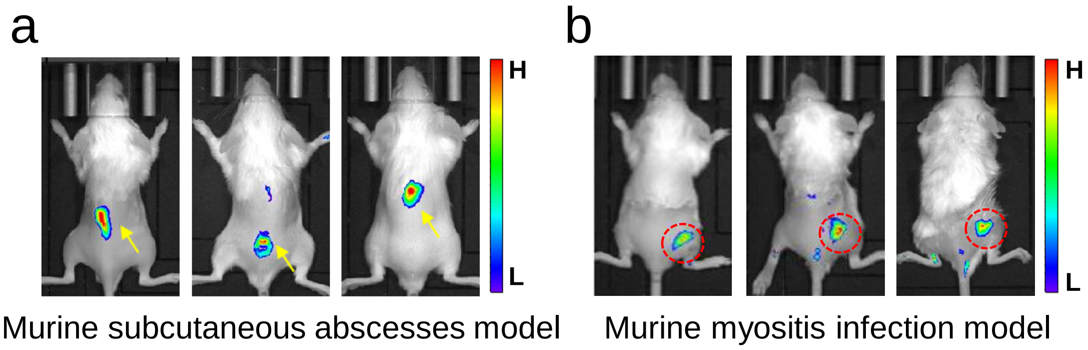

Figure S26. Whole body fluorescence images of mice 48 h after infection by bla-positive *E. coli* using **BIN-3**. Fluorescence images of mice with subcutaneous abscesses under the dorsum underneath the thin skeletal muscle (a) and myositis infection in the muscle of the right rear thigh (b). $\lambda_{ex/em}$ = 660/710 nm.

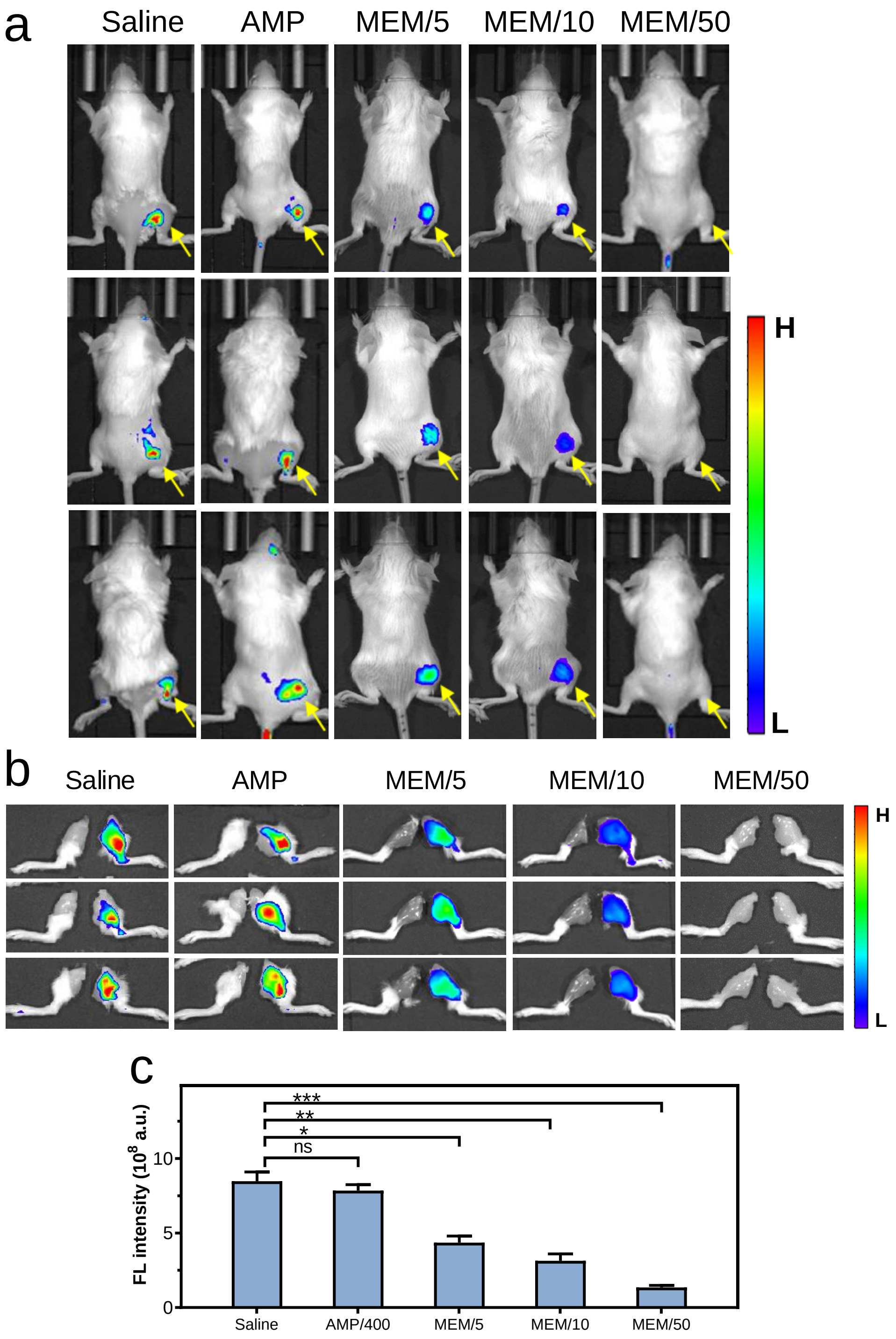

Figure S27. a) Fluorescence image of mice with infection by TEM-1 encoded *E. coli* after intraperitoneally administration of indicated antibiotics. b) Fluorescence images of rear thighs. c) Quantified fluorescence intensity of infected thighs at 5 h post-administration. $\lambda_{ex/em}$ = 660/710 nm. Error bars mean ± SD (ns: not significant; * $P < 0.05$; ** $P < 0.01$; *** $P < 0.001$, n = 3 technical replicates).

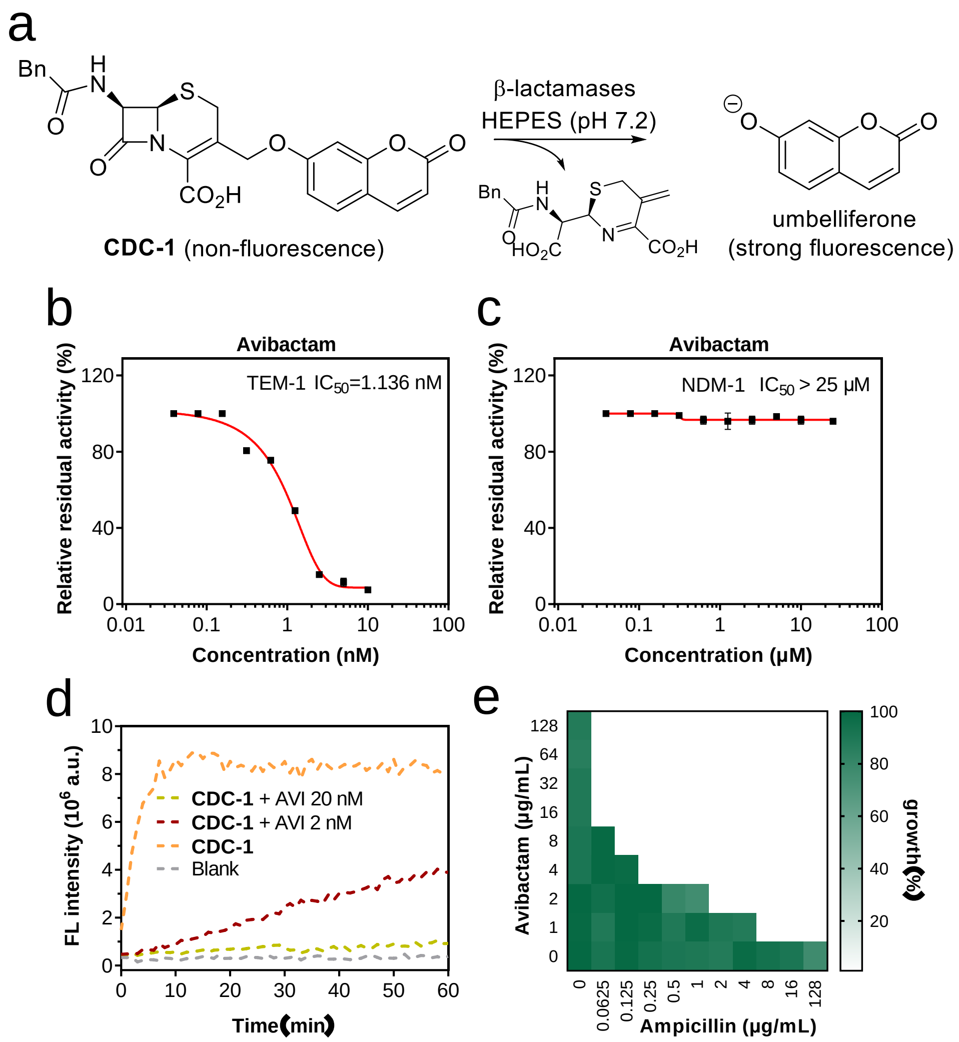

Figure S28. *In vitro* inhibitory activity of TEM-1 bla inhibitor, avibactam (AVI). a) Hydrolysis of **CDC-1** by β-lactamases produces fluorescent umbelliferone; b) Dose-response of avibactam against TEM-1. c) Dose-response of avibactam against NDM-1. d) Time-course fluorescence intensity of **CDC-1** (10 μM) in PBS in the presence of avibactam and TEM-1-encoded *E. coli* (1 x 10^8 CFU). $\lambda_{ex/em}$ = 365/460 nm. e) Heat map obtained from checkerboard analyses of TEM-1-producing *E. coli* (*E. coli* DH5α-pBBR1-TEM-1, MIC for ampicillin > 128 μg/mL). Error bars mean ± SD (n = 3 technical replicates).

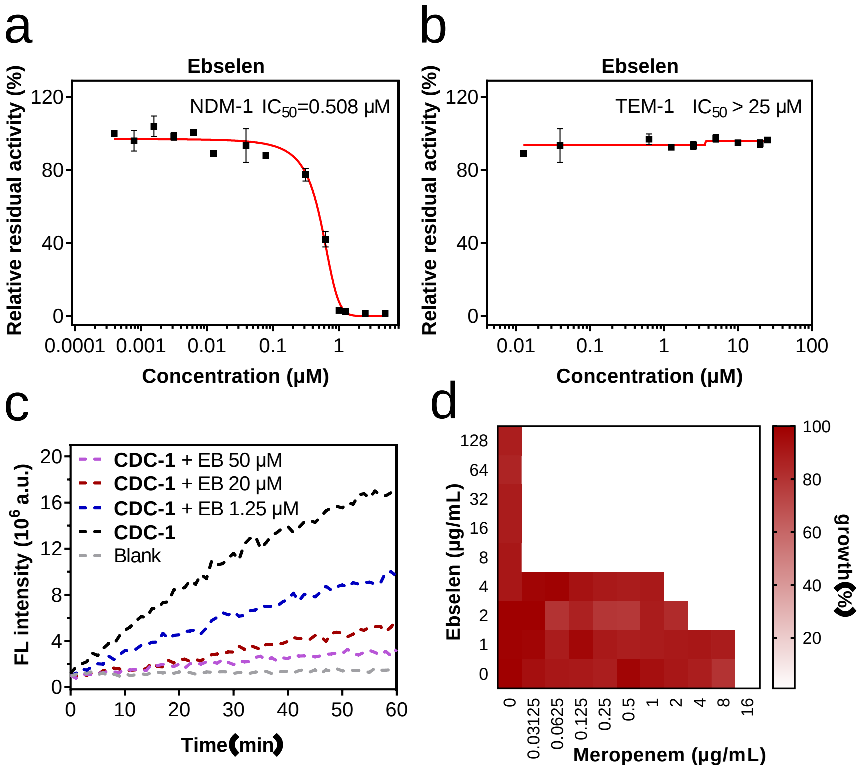

Figure S29. *In vitro* inhibitory activity of MBL inhibitor ebselen (EB). a) Dose-response of ebselen against NDM-1. b) Dose-response of avibactam against TEM-1. c) Time-course fluorescence intensity of **CDC-1** (10 μM) in PBS in the presence of avibactam and NDM-1-encoded *E. coli* (1 x 10^8 CFU). $\lambda_{ex/em}$ = 365/460 nm. d) Heat map obtained from checkerboard analyses of NDM-1-producing *E. coli* (*E. coli* DH5α-pUC19-NDM-1, MIC for meropenem = 16 μg/mL). Error bars mean ± SD (n = 3 technical replicates).

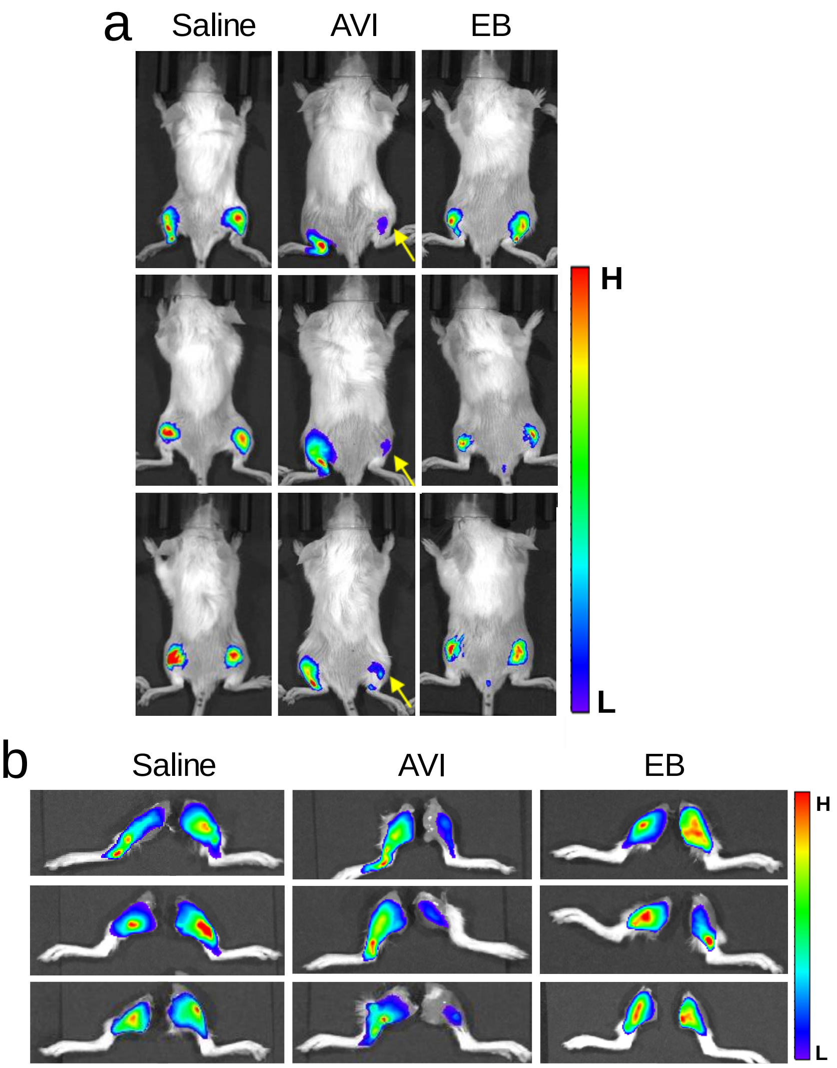

Figure S30. a) Fluorescence images of mice with the left rear thigh infected by NDM-1-encoded *E. coli* and the right rear thigh infected by TEM-1-encoded *E. coli* after *i.v.* administration of indicated inhibitors (AVI, avibactam, a SBL inhibitor; EB, ebselen, a MBL inhibitor). b) Fluorescence images of infected thighs after *i.v.* administration of indicated inhibitors. $\lambda_{ex/em}$ = 660/710 nm.

General Information

Unless otherwise noted, all reagents were obtained commercially and used without further purification. The ^{1}H and ^{13}C NMR spectra were taken on Bruker nuclear magnetic resonance spectrometer (400 MHz for ^{1}H; 100 or 150 MHz for ^{13}C NMR). Chemical shifts were reported in parts per million (ppm, δ) downfield from tetramethylsilane (δ = 0.00 ppm). Proton coupling patterns are described as singlet (s), doublet (d), triplet (t), quartet (q), and multiplet (m). High-resolution mass spectra (HRMS) were recorded on a Bruker micro-TOF-QII time of flight mass spectrometer with electrospray ionization. HPLC was performed on a Shimadzu HPLC System equipped with a LC-20AT gradient pump and an inline diode array UV-Vis detector. A reversed-phase C18 (Inertsil ODS-SP, 5 μm, 4.6 x 250 mm or phenomenex, 5 μm, 21.2 x 250 mm) column was used with a MeCN/H_2O gradient mobile phase containing 0.1% trifluoroacetic acid at a flow of 1 or 12 mL/min for the analysis or purification.

The plasmid pBBR1MCS2-Tac-mCherry was purchased from Shanghai Hewu Biotechnology Co., LTD, plasmid pUC19 from Takara Bio. The β-lactamases TEM-1 and NDM-1 were purified and obtained as previously described [1]. *E. coli* DH5α was purchased from Takara Bio and BSA (bovine serum albumin) from Aladdin, and BI FBS from Bioind. Lysozyme, β-glucosidase, lipase, trypsin, Glutathione were obtained from Biosharp, amino acids (Lys, Cys and Trp) from Adamas. Bacteria, including *E. coli* (ATCC 25922), *K. pneumoniae* (ATCC BAA 2146), *E. coli* (ATCC 35218) and *E. cloacae* (ATCC BAA 1143) were obtained from Microbiologics. HEK293 cells were obtained from Cell Bank of the Chinese Academy of Sciences (Shanghai, China). Bla-positive *E. coli* DH5α-TEM-1 and *E. coli* DH5α-NDM-1 refer to *E. coli* transformed by plasmid pBBR1-Tac-TEM-1 and pUC19-pre-NDM-1, respectively.

Absorbance spectrum was recorded on a UV1800 Series UV-Vis spectrophotometer (Shimadzu, Japan). Fluorescence spectrum was recorded on a wavelength- calibrated FluoroMax-3 fluorometer (Horiba Jobin Yvon, France). In-gel fluorescence scanning was taken using a Gel Documentation and Typhoon TRIO Variable Mode Imager System (GE Healthcare, USA). Absorbance for CCK-8 assay was determined in a microplate reader (Molecular Devices, SpectraMax *i*3). Cell images were taken using fluorescence microscope (Leica, Germany) and confocal microscope (Leica, Germany). Image processing was made on image J software (National Institutes of Health, USA). *In vivo* images were obtained by IVIS Lumina XRMS Series III *in vivo* Imaging System (PerkinElmer, Inc. USA).

Synthesis and Characterization

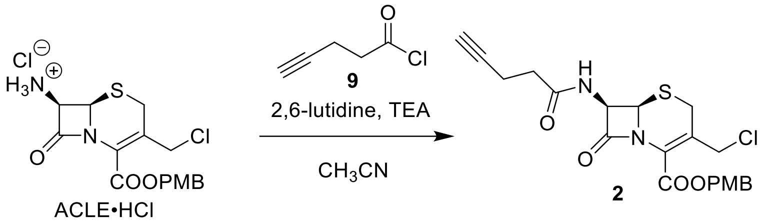

4-methoxybenzyl (6R,7R)-3-(chloromethyl)-8-oxo-7-(pent-4-ynamido)-5-thia-1-azabicyclo[4.2.0]oct-2-ene-2-carboxylate (2)

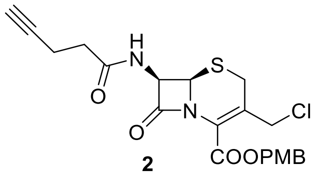

To a solution of 4-pentynoic acid (0.6534 g, 6.661 mmol) in DCM (anhydrous, 1.0 mL) at 0 °C were added DMF (anhydrous, 50 μL), oxalyl chloride (715 μL, 8.450 mmol) and the resulting mixture were stirred at 0 °C for 1.5 h. Volatile solvent and excess reagents were then removed under vacuum to afford pent-4-ynoyl chloride **9** as crude product, which was used in the next step without further purification.

To a mixture of ACLE·HCl [2] (1.7480 g, 4.313 mmol), 2,6-lutidine (975 μL, 8.644 mmol), and TEA (510 μL, 3.669 mmol) in CH_3CN (anhydrous, 10 mL) at 0 °C was added a solution of **9** (prepared above) in CH_3CN (anhydrous, 5 mL) and the resulting mixture were stirred at rt for 12 h. After removal of the solvent under reduced pressure, DCM (100 mL) was added and the solution was washed with water (60 mL x 3). The organic layer was dried over Na_2SO_4 and concentrated. Purification by flash chromatography on a silica gel column afforded the title compound as a white solid (1.0475 g, 54%). ^{1}H NMR (400 MHz, $CDCl_3$) δ 7.34 (d, *J* = 8.8 Hz, 2H), 6.90 (d, *J* = 8.8 Hz, 2H), 6.38 (d, *J* = 8.8 Hz, 1H), 5.87 (dd, *J* = 9.2, 4.8 Hz, 1H), 5.31 – 5.19 (m, 2H), 4.97 (d, *J* = 4.8 Hz, 1H), 4.54 (d, *J* = 11.6 Hz, 1H), 4.44 (d, *J* = 11.6 Hz, 1H), 3.81 (s, 3H), 3.66 (d, *J* = 18.0 Hz, 1H), 3.49 (d, *J* = 18.0 Hz, 1H), 2.62 – 2.41 (m, 4H), 2.05 (t, *J* = 2.4 Hz, 1H). ^{13}C NMR (151 MHz, $CDCl_3$) δ 171.13, 164.74, 161.11, 160.00, 130.74, 126.59, 126.15, 125.65, 114.03, 82.31, 70.01, 68.30, 59.23, 57.61, 55.29, 43.26, 34.97, 27.22, 14.60.

2-((E)-2-(6-(((6R,7R)-2-(((4-methoxybenzyl)oxy)carbonyl)-8-oxo-7-(pent-4-yn-amido)-5-thia-1-azabicyclo[4.2.0]oct-2-en-3-yl)methoxy)-2,3-dihydro-1H-xanthen -4-yl)vinyl)-3,3-dimethyl-1-propyl-3H-indol-1-ium iodide (8)

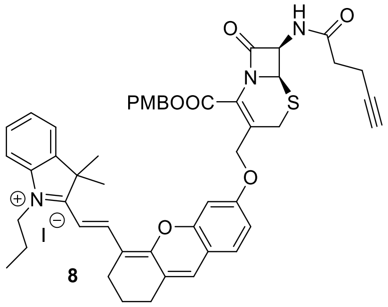

8

To a solution of **2** (197.2 mg, 0.439 mmol) in DMF (anhydrous, 1.0 mL) was added potassium iodide (134.1 mg, 0.878 mmol) and the reaction mixture were stirred at rt for 0.5 h before $KHCO_3$ (31.0 mg, 0.310 mmol), 18-crown-6 (20.0 mg, 0.074 mmol), **7** [3] (62.4 mg, 0.116 mmol) were added. Upon being stirred at rt for 3 h, DCM (20 mL) and water (20 mL) were added to the reaction mixture. The organic layer was separated and the aqueous layer was extracted with DCM (30 mL x 3). The combined organic layers were washed with brine (30 mL x 1) and dried over Na_2SO_4. Purification by flash chromatography on a silica gel column afforded the title compound as a blue solid (62.2 mg, 57%). ^{1}H NMR (600 MHz, CD_3OD) δ 8.75 (d, *J* = 15.0 Hz, 1H), 7.60 – 7.49 (m, 3H), 7.47 – 7.40 (m, 2H), 7.38 (s, 1H), 7.29 (d, *J* = 9.0 Hz, 2H), 7.10 (d, *J* = 1.8 Hz, 1H), 6.90 (dd, *J* = 9.0, 2.4 Hz, 1H), 6.79 (d, *J* = 8.4 Hz, 2H), 6.54 (d, *J* = 15.0 Hz, 1H), 5.78 (d, *J* = 4.8 Hz, 1H), 5.30 (d, *J* = 12.0 Hz, 1H), 5.23 (d, *J* = 12.0 Hz, 1H), 5.15 – 5.09 (m, 2H), 4.92 (d, *J* = 12.6 Hz, 1H), 4.34 (t, *J* = 7.8 Hz, 2H), 3.76 – 3.60 (m, 5H), 2.85 – 2.68 (m, 4H), 2.52 – 2.42 (m, 4H), 2.25 (s, 1H), 2.01 – 1.91 (m, 4H), 1.89 – 1.78 (m, 6H), 1.08 (t, *J* = 7.2 Hz, 3H). ^{13}C NMR (151 MHz, CD_3OD) δ 178.12, 173.07, 165.07, 162.03, 161.53, 161.47, 160.02, 154.34, 145.71, 142.17, 141.61, 133.41, 130.13, 128.83, 128.68, 127.52, 127.09, 126.88, 126.14, 125.52, 122.38, 116.23, 114.26, 114.05, 113.53, 112.64, 103.61, 100.96, 81.88, 69.06, 67.61, 66.89, 59.41, 57.67, 54.34, 50.69, 46.17, 34.04, 28.71, 27.27, 27.01, 25.39, 23.67, 20.91, 20.24, 14.05, 10.19.

2-((E)-2-(6-(((6R,7R)-2-carboxy-8-oxo-7-(pent-4-ynamido)-5-thia-1-azabicyclo [4.2.0]oct-2-en-3-yl)methoxy)-2,3-dihydro-1H-xanthen-4-yl)vinyl)-3,3-dimethyl-1-propyl-3H-indol-1-ium iodide (BIN-1)

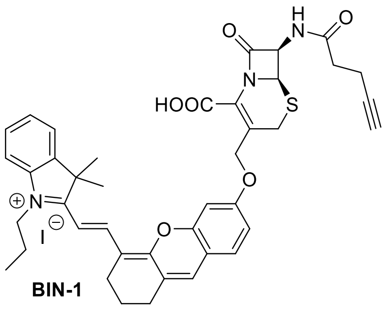

A solution of **8** (25.2 mg, 0.027 mmol) in a mixture of DCM/TFA/TIPS/H_2O (100:20:1:1, 2.4 mL) at 0 ºC was stirred at 0 ºC for 1.5 h (monitored by HPLC). DCM (10 mL) was added and the solution was washed with $NaHCO_3$ (aq. 10 mL x 1) and water (10 mL x 1) subsequently. The organic layer was dried over Na_2SO_4 and concentrated. RP-HPLC purification on a C18 column afforded the title compound (18.6 mg, 85%). ^{1}H NMR (600 MHz, *d*6-DMSO) δ 8.91 (d, *J* = 8.4 Hz, 1H), 8.61 (d, *J* = 15.0 Hz, 1H), 7.75 (d, *J* = 7.2 Hz, 1H), 7.72 (d, *J* = 7.8 Hz, 1H), 7.59 – 7.52 (m, 2H), 7.52 – 7.45 (m, 2H), 7.29 (d, *J* = 1.8 Hz, 1H), 7.03 (dd, *J* = 8.4, 1.8 Hz, 1H), 6.60 (d, *J* = 15.0 Hz, 1H), 5.74 (dd, *J* = 8.4, 4.8 Hz, 1H), 5.20 – 5.11 (m, 2H), 4.97 (d, *J* = 12.0 Hz, 1H), 4.41 (t, *J* = 7.2 Hz, 2H), 3.71 (d, *J* = 18.6 Hz, 1H), 3.60 (d, *J* = 18.6 Hz, 1H), 2.77 (s, 1H), 2.75 – 2.71 (m, 2H), 2.71 – 2.65 (m, 2H), 2.45 – 2.32 (m, 4H), 1.88 – 1.81 (m, 4H), 1.80 – 1.73 (m, 6H), 0.99 (t, *J* = 7.2 Hz, 3H). HRMS (ESI) m/z calcd for $C_{41}H_{42}N_3O_6S$ (M-I)$^+$ 704.2789, found 704.2798.

3-((2-(4-(3-(((6R,7R)-2-carboxy-3-(((4-((E)-2-(3,3-dimethyl-1-propyl-3H-indol-1-ium-2-yl)vinyl)-2,3-dihydro-1H-xanthen-6-yl)oxy)methyl)-8-oxo-5-thia-1-aza bicyclo[4.2.0]oct-2-en-7-yl)amino)-3-oxopropyl)-1H-1,2,3-triazol-1-yl)ethyl) dimethylammonio)propane-1-sulfonate iodide (BIN-2)

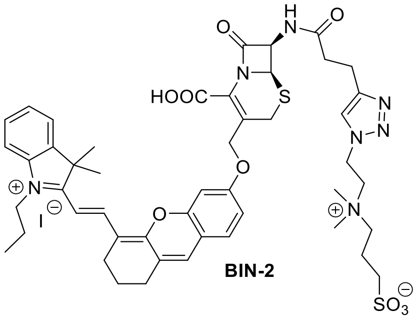

A mixture of **BIN-1** (9.1 mg, 0.011mmol), **6** [4] (11.3 mg, 0.048 mmol), $CuSO_4$ (11.6 mg, 0.073 mmol), *L*-(+)-Ascorbic acid (13.2 mg, 0.075 mmol) in DMSO (0.5 mL) and H_2O (0.1 mL) were stirred at rt for 1 h. The title compound was obtained as blue solid (8.0 mg, 69%) after purification by preparative RP-HPLC on a C18 column. ^{1}H NMR (600 MHz, *d*6-DMSO) δ 8.92 (d, *J* = 8.4 Hz, 1H), 8.61 (d, *J* = 15.0 Hz, 1H), 7.96

(d, J = 6.6 Hz, 1H), 7.75 (d, J = 7.2 Hz, 1H), 7.72 (d, J = 8.4 Hz, 1H), 7.58 – 7.53 (m, 2H), 7.51 – 7.45 (m, 2H), 7.29 (d, J = 2.4 Hz, 1H), 7.03 (dd, J = 8.4, 1.8 Hz, 1H), 6.60 (d, J = 15.0 Hz, 1H), 5.73 (dd, J = 7.8, 4.8 Hz, 1H), 5.19 – 5.11 (m, 2H), 4.96 (d, J = 12.0 Hz, 1H), 4.90 (t, J = 6.6 Hz, 2H), 4.41 (t, J = 7.2 Hz, 2H), 3.85 (t, J = 7.2 Hz, 2H), 3.72 (d, J = 18.6 Hz, 1H), 3.61 (d, J = 18.0 Hz, 1H), 3.54 – 3.51 (m, 2H), 3.06 (s, 6H), 2.90 – 2.87 (m, 2H), 2.75 – 2.72 (m, 2H), 2.71 – 2.66 (m, 2H), 2.58 – 2.54 (m, 2H), 2.47 (t, J = 6.6 Hz, 2H), 2.04 – 1.96 (m, 2H), 1.88 – 1.80 (m, 4H), 1.80 – 1.74 (m, 6H), 0.99 (t, J = 7.2 Hz, 3H). HRMS (ESI) m/z calcd for $C_{48}H_{58}N_7O_9S_2$ $(M-I)^+$ 940.3732, found 940.3738.

2-((E)-2-(7-(hydroxymethyl)-6-(((6R,7R)-2-(((4-methoxybenzyl)oxy)carbonyl)-8-oxo-7-(pent-4-ynamido)-5-thia-1-azabicyclo[4.2.0]oct-2-en-3-yl)methoxy)-2,3-dihydro-1H-xanthen-4-yl)vinyl)-3,3-dimethyl-1-propyl-3H-indol-1-ium iodide (3)

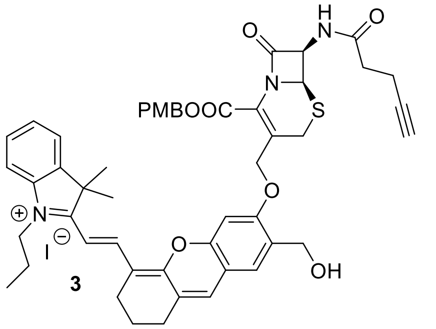

To a solution of **2** (238.7 mg, 0.532 mmol) in DMF (anhydrous, 1.2 mL) was added potassium iodide (151.9 mg, 0.915 mmol) and the reaction mixture was then stirred at rt for 0.5 h before $KHCO_3$ (38.6 mg, 0.386 mmol), 18-crown-6 (24.7 mg, 0.093 mmol), **1**[5] (103.1 mg, 0.181 mmol) were added. Upon being stirred at rt for 1.5 h, DCM (20 mL) and water (20 mL) were added to the reaction mixture. The organic layer was separated and the aqueous layer was extracted with DCM (30 mL x 3). The combined organic layers were washed with brine (30 mL x 1) and dried over Na_2SO_4. Purification by flash chromatography on a silica gel column afforded the title compound as a blue solid (99.4 mg, 56%). ^{1}H NMR (600 MHz, CD_3OD) δ 8.75 (d, J = 15.0 Hz, 1H), 7.56 (s, 1H), 7.55 – 7.48 (m, 3H), 7.45 (s, 1H), 7.40 (t, J = 7.2 Hz, 1H), 7.30 (d, J = 8.4 Hz, 2H), 7.23 (s, 1H), 6.79 (d, J = 8.4 Hz, 2H), 6.52 (d, J = 14.4 Hz, 1H), 5.79 (d, J = 4.8 Hz, 1H), 5.36 (d, J = 12.0 Hz, 1H), 5.28 – 5.21 (m, 2H), 5.15 (d, J = 4.8 Hz, 1H), 4.98 (d, J = 12.6 Hz, 1H), 4.68 – 4.60 (m, 2H), 4.33 (t, J = 7.2 Hz, 2H), 3.76 – 3.63 (m, 5H), 2.86 – 2.69 (m, 4H), 2.51 – 2.41 (m, 4H), 2.24 (s, 1H), 2.01 – 1.91 (m, 4H), 1.89 – 1.80 (m, 6H), 1.08 (t, J = 7.2 Hz, 3H). ^{13}C NMR (151 MHz, CD_3OD) δ 177.80, 173.06, 165.13, 162.19, 161.79, 160.03, 158.67, 153.88, 145.41, 142.09, 141.67, 134.02,

130.00, 129.14, 128.78, 127.60, 126.90, 126.80, 126.45, 126.05, 125.39, 122.33, 115.76, 114.23, 113.54, 112.47, 103.18, 99.16, 81.84, 69.02, 67.62, 66.95, 59.42, 58.30, 57.66, 54.32, 50.58, 46.06, 34.03, 28.72, 27.46, 27.07, 25.10, 23.73, 20.85, 20.24, 14.02, 10.19. HRMS (ESI) m/z calcd for $C_{50}H_{52}N_3O_8S$ $(M-I)^+$ 854.3470, found 854.3472.

2-((E)-2-(7-(((ethylcarbamoyl)oxy)methyl)-6-(((6R,7R)-2-(((4-methoxybenzyl)oxy) carbonyl)-8-oxo-7-(pent-4-ynamido)-5-thia-1-azabicyclo[4.2.0]oct-2-en-3-yl) methoxy)-2,3-dihydro-1H-xanthen-4-yl)vinyl)-3,3-dimethyl-1-propyl-3H-indol-1-ium iodide (4)

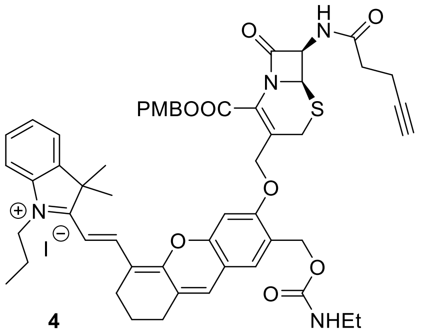

4

Under N_2 atmosphere, to a solution of compound **9** (70.9 mg, 0.072 mmol) in DMF (anhydrous, 0.7 mL) were added isocyanatoethane (58 μL, 0.723 mmol) and DBTDL (18 μL, 0.029 mmol) and the resulting mixture were stirred at rt for 4 h. After dilution with DCM (10 mL), the solution was washed with water (10 mL x 3). The organic layer was dried over Na_2SO_4 and concentrated. The residue was purified by chromatography on silica gel column to afford title compound as blue solid (63.5 mg, 83%). ^{1}H NMR (600 MHz, CD_3OD) δ 8.74 (d, *J* = 14.4 Hz, 1H), 7.56 (d, *J* = 8.4 Hz, 1H), 7.54 – 7.47 (m, 3H), 7.42 (t, *J* = 7.2 Hz, 1H), 7.38 (s, 1H), 7.30 (d, *J* = 8.4 Hz, 2H), 7.20 (s, 1H), 6.78 (d, *J* = 8.4 Hz, 2H), 6.55 (d, *J* = 15.0 Hz, 1H), 5.80 (d, *J* = 4.8 Hz, 1H), 5.36 (d, *J* = 12.0 Hz, 1H), 5.28 – 5.20 (m, 2H), 5.17 – 5.06 (m, 3H), 4.96 (d, *J* = 12.6 Hz, 1H), 4.34 (t, *J* = 7.2 Hz, 2H), 3.77 – 3.62 (m, 5H), 3.15 (q, *J* = 7.2 Hz, 2H), 2.85 – 2.71 (m, 4H), 2.50 – 2.41 (m, 4H), 2.24 (s, 1H), 2.01 – 1.92 (m, 4H), 1.90 – 1.79 (m, 6H), 1.12 (t, *J* = 7.2 Hz, 3H), 1.08 (t, *J* = 7.8 Hz, 3H). ^{13}C NMR (151 MHz, CD_3OD) δ 178.13, 173.07, 165.14, 162.14, 161.37, 160.02, 158.95, 157.10, 154.23, 145.58, 142.19, 141.62, 133.25, 130.02, 128.83, 127.88, 127.84, 127.10, 126.80, 125.86, 125.51, 124.42, 122.36, 115.65, 114.28, 113.53, 112.65, 103.69, 99.42, 81.85, 69.03, 67.63, 67.08, 60.71, 59.41, 57.65, 54.33, 50.71, 46.17, 35.30, 34.04, 28.75, 27.38, 27.01, 25.21, 23.71, 20.91, 20.20, 14.04, 13.98, 10.19. HRMS (ESI) m/z calcd for $C_{53}H_{57}N_4O_9S$ $(M-I)^+$ 925.3841, found 925.3841.

2-((E)-2-(6-(((6R,7R)-2-carboxy-8-oxo-7-(pent-4-ynamido)-5-thia-1-azabicyclo [4.2.0]oct-2-en-3-yl)methoxy)-7-(((ethylcarbamoyl)oxy)methyl)-2,3-dihydro-1H-xanthen-4-yl)vinyl)-3,3-dimethyl-1-propyl-3H-indol-1-ium iodide (5)

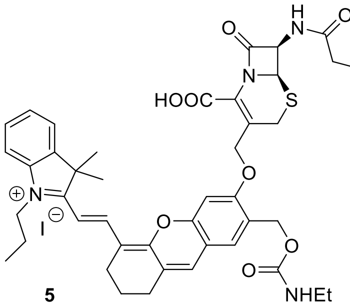

5

To a solution of DCM: TFA: TIPS: H_2O = 100:20:1:1 (2.4 mL) at 0 °C was added compound **4** (15.2 mg, 0.014 mmol) and the mixture was stirred at 0 °C for 1.5 h (monitored with HPLC). DCM (10 mL) was added and the solution was washed with 1M $NaHCO_3$ (aq. 7 mL x 1) and water (10 mL x 1) subsequently. The organic layer was dried over Na_2SO_4 and concentrated. RP-HPLC purification on a C18 column afforded the title compound (10.0 mg, 74%). ^{1}H NMR (600 MHz, CD_3OD) δ 8.78 (d, *J* = 15.0 Hz, 1H), 7.69 (d, *J* = 7.2 Hz, 1H), 7.59 (s, 1H), 7.58 – 7.49 (m, 3H), 7.46 (t, *J* = 7.8 Hz, 1H), 7.36 (s, 1H), 6.54 (d, *J* = 15.0 Hz, 1H), 5.75 (d, *J* = 4.8 Hz, 1H), 5.58 (d, *J* = 13.2 Hz, 1H), 5.22 – 5.04 (m, 4H), 4.34 (t, *J* = 7.2 Hz, 2H), 3.67 (d, *J* = 18.6 Hz, 1H), 3.61 (d, *J* = 18.0 Hz, 1H), 3.15 (q, *J* = 7.2 Hz, 2H), 2.82 – 2.76 (m, 2H), 2.74 – 2.69 (m, 2H), 2.46 – 2.39 (m, 4H), 2.22 (s, 1H), 1.98 – 1.92 (m, 4H), 1.88 – 1.82 (m, 6H), 1.12 (t, *J* = 7.2 Hz, 3H), 1.07 (t, *J* = 7.2 Hz, 3H). HRMS (ESI) m/z calcd for $C_{45}H_{49}N_4O_8S$ (M-I)$^+$ 805.3266, found 805.3267.

3-((2-(4-(3-(((6R,7R)-2-carboxy-3-(((4-((E)-2-(3,3-dimethyl-1-propyl-3H-indol-1-ium-2-yl) vinyl)-7-(((ethylcarbamoyl)oxy)methyl)-2,3-dihydro-1H-xanthen-6-yl)oxy)methyl)-8-oxo-5-thia-1-azabicyclo[4.2.0]oct-2-en-7-yl)amino)-3-oxopropyl) -1H-1,2,3-triazol-1-yl)ethyl)dimethylammonio)propane-1-sulfonate iodide (BIN-3)

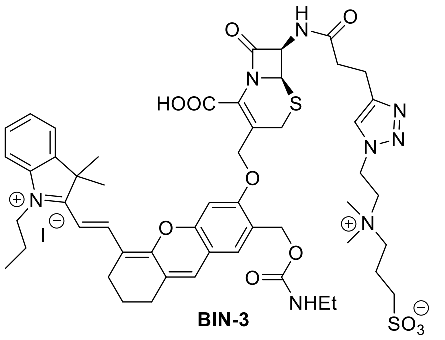

BIN-3

A mixture of **5** (7.1 mg, 0.008 mmol), **6** (13.2 mg, 0.056 mmol), $CuSO_4$ (15.1 mg,

0.095 mmol), *L*-(+)-Ascorbic acid (14.9 mg, 0.085 mmol) in DMSO (0.5 mL) and H_2O (0.1 mL) were stirred at rt for 1 h. The title compound was obtained as a blue solid (6.2 mg, 70%) after purification by preparative RP-HPLC on a C18 column. The purity of title compound was confirmed by HPLC analysis. HRMS (ESI) m/z calcd for $C_{52}H_{65}N_8O_{11}S_2$ $(M-I)^+$ 1041.4209, found 1041.4219.

2-((E)-2-(6-(((6R,7R)-2-(((4-methoxybenzyl)oxy)carbonyl)-8-oxo-7-(2-phenyl-acetamido)-5-thia-1-azabicyclo[4.2.0]oct-2-en-3-yl)methoxy)-2,3-dihydro-1H-xanthen-4-yl)vinyl)-3,3-dimethyl-1-propyl-3H-indol-1-ium (10)

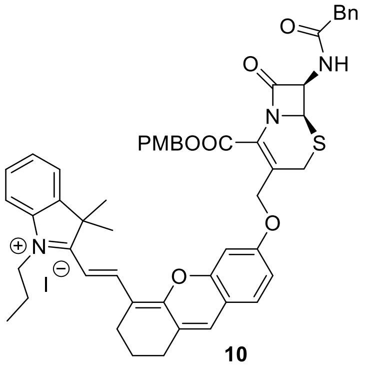

10

The title compound was prepared as compound **8**. The ^{1}H NMR spectrum is consistent with previously report[6] but without the formation of the Δ_3 isomer. ^{1}H NMR (400 MHz, DMSO-d_6) δ = 9.14 (d, *J* = 8.3, 1H), 8.59 (d, *J* = 14.9, 1H), 7.71 (d, *J* = 8.0, 1H), 7.68 (d, *J* = 7.4, 1H), 7.56 – 7.50 (m, 3H), 7.45 (t, *J* = 7.5, 1H), 7.31 – 7.24 (m, 10H), 7.09 (d, *J* = 2.4, 1H), 6.95 (dd, *J* = 8.6, 2.4, 1H), 6.81 (d, *J* = 8.6, 2H), 6.61 (d, *J* = 15.0, 1H), 5.75 (dd, *J* = 8.3, 4.9, 1H), 5.22 (s, 2H), 5.17 (d, *J* = 4.9, 1H), 4.97 (d, *J* = 12, 1H), 4.88 (d, *J* = 12, 1H), 4.40 (t, *J* = 7.2, 2H), 3.67 (s, 6H), 2.76 – 2.67 (m, 4H), 1.81 – 1.86 (m, 4H), 1.77 (d, *J* = 3.8, 6H), 0.99 (t, *J* = 7.4, 3H).

2-((E)-2-(6-(((6R,7R)-2-carboxy-8-oxo-7-(2-phenylacetamido)-5-thia-1-azabicyclo[4.2.0]oct-2-en-3-yl)methoxy)-2,3-dihydro-1H-xanthen-4-yl)vinyl)-3,3-dimethyl-1-propyl-3H-indol-1-ium

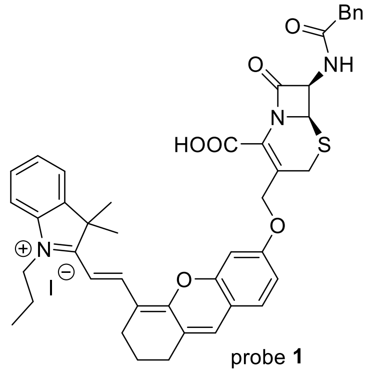

probe **1**

The title compound was prepared as probe **BIN-1**. The ^{1}H NMR spectrum is consistent with previously report[6] but contain no the Δ_3 isomer. ^{1}H NMR (400 MHz, DMSO-d_6) δ = 9.13 (d, J = 8.3, 1H), 8.60 (d, J = 14.9, 1H), 7.75 (d, J = 7.0, 1H), 7.72 (d, J = 8.0, 1H), 7.52 – 7.57 (m 2H), 7.51 – 7.45 (m, 2H), 7.32 – 7.20 (m, 6H), 7.03 (dd, J = 8.7, 2.4, 1H), 6.60 (d, J = 15.0, 1H), 5.58 (dd, J = 8.3, 4.8, 1H), 5.18 – 5.13 (m, 2H), 4.96 (d, J = 12.3, 1H), 4.40 (t, J = 7.2, 2H), 3.58 – 3.73 (m, 4H), 2.74 – 2.66 (m, 4H) 1.88 – 1.80 (m, 4H), 1.77 (d, J = 6.7, 6H), 0.98 (t, J = 7.4, 3H).

Enzyme-Based Investigations

General Procedure for Enzymatic Assays

A mixture of fluorescence probe (**BIN-3**/**BIN-2**, 5 μM) and β-lactamase TEM-1 (100 nM) was incubated at 37 °C for 15 min in PBS (pH 7.4). UV-Vis spectra were recorded on a UV1800 Series UV-Vis spectrophotometer and the fluorescence spectra were recorded on a wavelength-calibrated FluoroMax-3 fluorimeter. HPLC analysis of the mixture was performed on a C18 column with water and acetonitrile (containing 0.1% TFA) as eluent.

Specificity Test of BIN-3

To evaluate the selectivity of probe, **BIN-3**/**BIN-2** (5 μM) was incubated with a variety of analytes, including enzymes (TEM-1, lysozyme, β-glucosidase, lipase, trypsin and BSA) glutathione, and amino acids (Lys, Cys and Trp) at 37 °C for 30 min, respectively. The fluorescence intensity was then recorded on a wavelength-calibrated FluoroMax-3 fluorimeter. Each experiment was triplicated.

In-gel Fluorescence Imaging of Protein upon Incubation with BIN-3

BIN-3/**BIN-2** (10 μM) was incubated with TEM-1 (5 μM) in the presence or absence of BSA (10 μM) at 37 °C for 2 h. These samples were then analyzed by SDS-PAGE gel

and imaged with an Odyssey SA near-infrared two-color laser imaging system, followed by staining with Coomassie brilliant blue.

Assay for IC_{50} of β-Lactamase Inhibitor

The IC_{50} was determined following the reported protocol [7] with minor modifications. Briefly, a solution of enzyme (TEM-1: 5 nM; NDM-1: 2 nM) was incubated with inhibitor (avibactam or ebselen) in 50 mM HEPES buffer (pH 7.2) containing 0.01% triton in a 96-well plate for 10 minutes before the addition of fluorogenic substrate **CDC-1** (10 μM) and monitoring fluorescence intensity at 460 nm (excitation at 365 nm) continuously over a period of 30 minutes at 37 °C with a microplate reader. The initial velocity (V_0) was calculated using the linear portions of the curves. Each experiment was triplicated. The IC_{50} was obtained by fitting a dose-response curve using Graph Pad Prism 7 software.

Bacteria-Related Experiments

Construction of the β-Lactamase-Expressing Plasmids and the Transformation to *E. coli*

The TEM-1-expression plasmid, pBBR1MCS2-Tac-TEM-1, was constructed by simply replacing the mCherry gene of pBBR1MCS2-Tac-mCherry plasmid with *bla*$_{\text{TEM-1}}$ (see Table S2 for primers) following manufacture's protocol. The resulting PCR products were then treated with DpnI restriction enzyme to digest the template and the mCherry-free pBBR1 vector was obtained upon purification. PCRs were conducted with *E. coli* (ATCC-35218) as template DNA for TEM-1 (see Table S2 for primers). Homologous recombination of the purified PCR and the mCherry-free pBBR1 vector were conducted with Ezmax One-step Cloning kit (Tolo Biotech). The resulting pBBR1MCS2-Tac-TEM-1 plasmid was transformed into competent *E. coli* DH5α to obtain the desired TEM-1 expressing *E. coli*.

The NDM-1 expression plasmid, pUC19-pre-NDM-1, was constructed by inserting the full-length *bla*$_{\text{NDM-1}}$ into pUC19 XbaI (see Table S2 for primers) following manufacture's protocol. The linear vector pUC19 was obtained by digesting the vector pUC19 with XbaI. Subsequently, the template DNA was digested with DpnI restriction enzyme and the linear vector pUC19-XbaI was obtained upon purification. Meanwhile, the full-length *bla*$_{\text{NDM-1}}$ was obtained with a clinical isolate of *K. pneumoniae* ATCC BAA-2146 as template DNA (see Table S2 for primers). The purified PCR and the pUC19-XbaI vector underwent homologous recombination using the Ezmax One-step

Cloning kit (Tolo Biotech). The resulting pUC19-pre-NDM-1 plasmid was then transformed into competent *E. coli* DH5α to obtain the desired NDM-1 expressing *E. coli.*

Minimum Inhibitory Concentration (MIC) Assay

The MIC of antibiotic was determined by the microdilution method according to the Clinical and Laboratory Standards Institute (CLSI) guidelines.[8] Briefly, bacterial strains were streaked for single colonies on LB plates from frozen glycerol stocks stored at -80 ºC. Bacteria were selected from the plates and cultured in MH medium for 3 h at 37 ºC under shaking at 200 rpm. At this time, bacterial count is almost 3 x 10^8 CFU/mL, the cells were diluted in MHB to a cell density of 2 x 10^5 CFU/mL as the 2-fold working suspension. A 100 μL portion of inoculum was added to a treatment plate containing 2-fold serial dilutions of compound in MHB (100 μL/well) to lend a final total volume of 200 μL/well and a final inoculum density of ~1 x 10^5 CFU/mL. The completed assay plate was sealed with Parafilm and incubated at 37 ºC for 16-20 h. The MIC was read as the lowest treatment concentration where no bacterial growth occurred, as determined by OD_{600} measurements on a microplate 96-well reader.

Bacteria Viability Assay

E. coli (ATCC 25922) or *E. cloacae* (ATCC BAA 1143) (1 x 10^5 CFU/mL) were cultured in Luria-Bertani (LB) medium containing **BIN-3** or **BIN-2** (50 μM) at 37 °C with shaking at 200 rpm. At 0, 1, 3.5, 5, 7 and 10 h, an aliquot of culture medium was taken to count the viable bacterial cells by spotting on LB agar plates. Each experiment was triplicated.

Determination of Concentration of HD Dye in Bacteria Lysates and Incubation Medium

Bacteria *E. cloacae* (ATCC BAA 1143) or *E. coli* (ATCC 25922) in LB was inoculated into test tubes and incubated overnight at 37 °C with shaking at 200 rpm. Bacterial concentration was determined by the measurement of OD_{600} (OD_{600} = 1, 1 x 10^9 CFU/mL). Bacteria were collected by centrifugation and the pellet was re-suspended in PBS. Bacteria (1 x 10^{10} CFU) were treated with **BIN-3** or **BIN-2** (2.5 μM) in PBS (pH 7.4, 500 μL) at 37 °C for 2 h. After centrifugation, the incubation medium and the bacterial pellet were collected. The bacteria pellet were washed twice with PBS and re-suspended in PBS. Bacteria Lysis Buffer was added according to the manufacture's protocol (Sangon biotech, C500003) to afford the bacterial lysates. The fluorescence

spectra of the bacterial lysate and incubation medium before and after incubation with TEM-1 (100 nM) and BSA (10 μM, for **BIN-3** only) for 1 h were subsequently recorded using a fluorimeter.

To obtain a standard curve of fluorescence intensity versus concentration of HD dye, a range of concentrations of **BIN-3** or **BIN-2** (from 0 to 5 μM) were incubated with TEM-1 (100 nM) and BSA (10 μM, for **BIN-3** only) for 1 h, and the fluorescence intensity at 710 nm was recorded under excitation at 685 nm. The fluorescence intensity and the concentration of probe were plotted as a standard curve.

Bacteria Inhibition Assay

Briefly, bla-positive bacteria DH5a-TEM-1 or DH5a-NDM-1 (5 x 10^7 CFU) were incubated with inhibitors (avibactam or ebselen) in HEPES buffer (pH 7.2) in a 96-well plate for 10 minutes. The fluorescence intensity at 460 nm (excitation wavelength of 365 nm) was continuously monitored for 60 minutes at 37 °C using a microplate reader after adding a fluorescent substrate **CDC-1** (10 μM). Dose-response curves were fitted using GraphPad Prism 7.

Checkboard Broth Microdilution Assay

Briefly, 1 x 10^5 CFU/mL bacterial cells were inoculated into 100 μL MHB containing β-lactam and appropriately diluted inhibitor. The final concentration of DMSO was kept below 0.1%. The tested plates were then incubated at 37 ºC for 16 to 20 hours. The MIC was determined as the lowest concentration of antibiotic tested in the absence of visible bacterial growth. A microplate reader was used to read the optical density at 600 nm to generate heat maps. The heat map was obtained by using Graph Pad Prism 7 software.

Cell-Related Experiments

Cell Culture Conditions

HEK293 cells were cultured in high-glucose DMEM (Gibco) medium containing 10% fetal bovine serum (FBS) and 1% penicillin/streptomycin (PS) in 5% CO_2 humidified atmosphere (95%) at 37 °C.

Cell Viability Assays

The cytotoxicity of **BIN-3**/**BIN-2** to HEK293 cells was evaluated by Cell Counting Kit-8 (CCK-8) following manufacture’s protocol. In brief, cells (4 x 10^3 cells/well) in DMEM (supplemented with 10% FBS, 100 μL) were seeded in 96-well plate and

incubated at 37 °C overnight. Medium was then removed and cells were treated with **BIN-3** at a serial of concentrations (0, 10, 20, 40 and 100 μM) in DMEM (supplemented with 10% FBS, 100 μL) at 37 °C with 5% CO_2 and 95% humidity for 24 h. Add 10 μL of CCK-8 solution to each well and incubate at 37 °C with 5% CO_2 and 95% humidity for 1-4 h. Absorbance at 450 nm was measured with microplate reader and cell viability was calculated based on untreated wells. Each experiment was triplicated.

Hemolysis Assays

Fresh mouse red blood cells (RBCs) were washed three times with PBS (pH 7.4) and diluted to 5% (v/v) working suspension of RBCs. The probes were diluted with PBS. Then 50 μL of the above probes were added to an equal volume of RBC working suspension to give a final total volume of 200 μL/well and incubated at 37 °C for 1 h. RBCs in PBS only and RBCs treated with 0.1% Triton X-100 were used as blank and positive control, respectively. After centrifugation at 3000 rpm for 5 minutes, 100 μL of supernatant from each well was transferred to another 96-well plate. Finally, OD_{540} was measured to calculate the percentage of hemolysis. Each experiment was triplicated.

Animal-Related Experiments

In Vivo Cytotoxicity of Probe

The probe safety evaluation experiment was to determine blood routine testing and blood biochemical indexes and tissue staining to check the safety of the probes [9]. BALB/c female mice at 4-6 weeks old were obtained from Shanghai Laboratory Animal Research Center. Mice were housed at 25 °C with free access to food and water. The tissue staining experiment was to divide the mice into 3 groups, 3 in each group, saline (*i.v.* injection), **BIN-3** and **BIN-2** (1 μmol/kg, *i.v.* injection). 24 hours after injection, these mice were euthanized and dissected. The organs (heart, lung, liver, spleen and kidney) of the mice were sectioned and stained with H&E staining. The blood routine experiment is to divide the mice into 3 groups, 3 in each group, saline (*i.v.* injection), **BIN-3** and **BIN-2** (1 μmol/kg, *i.v.* injection). 24 hours post the injection, blood was taken for routine testing in each group.

The Biological Metabolism of Probe

The murine myositis infection model was constructed as previous report [10]. For the thigh myositis infection model, 0.1 mL of a bacterial suspension (5 x 10^8 CFU per thigh) was injected into the right rear thigh muscle of each mouse. Saline, **BIN-3** or **BIN-2** (1

μmol/kg) was administered through tail vein to mice after infection in the muscle of rear right thigh. Mouse urine was collected at different times and subjected to IVIS imaging with TEM-1 (100 nM) or β-ME (10 mM), and HPLC analysis with UV quantification at 600 nm was performed.

References

[1] (a) W. Mao, L. Xia, H. Xie, *Angew. Chem. Int. Ed.* **2017**, *56*, 4468-4472; (b) L. Hu, R. Liu, Z. Ma, T. Yu, Z. Li, Y. Zou, C. Yuan, F. Chen, H. Xie, *Chem. Commun.* **2021**, *57*, 13586-13589.

[2] L. Hu, H. Yang, T. Yu, F. Chen, R. Liu, S. Xue, S. Zhang, W. Mao, C. Ji, H. Wang, H. Xie, *Eur. J. Med. Chem.* **2022**, *232*, 114174.

[3] T. M. Ebaston, F. Nakonechny, E. Talalai, G. Gellerman, L. Patsenker, *Dyes Pigm.* **2021**, *184*, 108854.

[4] J. Huang, W. Xu, *J. Appl. Polym. Sci.* **2011**, *122*, 1251-1257.

[5] Y. Li, C. Zhang, Q. Wu, Y. Peng, Y. Ding, Z. Zhang, X. Xu, H. Xie, *Angew. Chem., Int. Ed.* **2024**, *63*, e202317773.

[6] L. H. Li, Z. Li, W. Shi, X. H. Li, H. M. Ma, *Anal. Chem.* **2014**, *86*, 6115-6120.

[7] (a) H. Xie, J. Mire, Y. Kong, M. Chang, H. A. Hassounah, C. N. Thornton, J. C. Sacchettini, J. D. Cirillo, J. Rao, *Nat. Chem.* **2012**, *4*, 802-809; (b) S. S. van Berkel, J. Brem, A. M. Rydzik, R. Salimraj, R. Cain, A. Verma, R. J. Owens, C. W. G. Fishwick, J. Spencer, C. J. Schofield, *J. Med. Chem.* **2013**, *56*, 6945-6953.

[8] Performance standards for antimicrobial susceptibility testing; 29th informational supplement. CLSI Document M100, 2019, Wayne, PA: CLSI.

[9] Z. Chen, L. Su, Y. Wu, J. Liu, R. Wu, Q. Li, C. Wang, L. Liu, J. Song, *Proc. Natl. Acad. Sci. U.S.A.* **2023**, *120*, e2205186120.

[10] X. Ning, S. Lee, Z. Wang, D. Kim, B. Stubblefield, E. Gilbert, N. Murthy, *Nat. Mater.* **2011**, *10*, 602-607.

^{1}H and ^{13}C NMR Spectra

^{1}H and ^{13}C NMR Spectra of 2

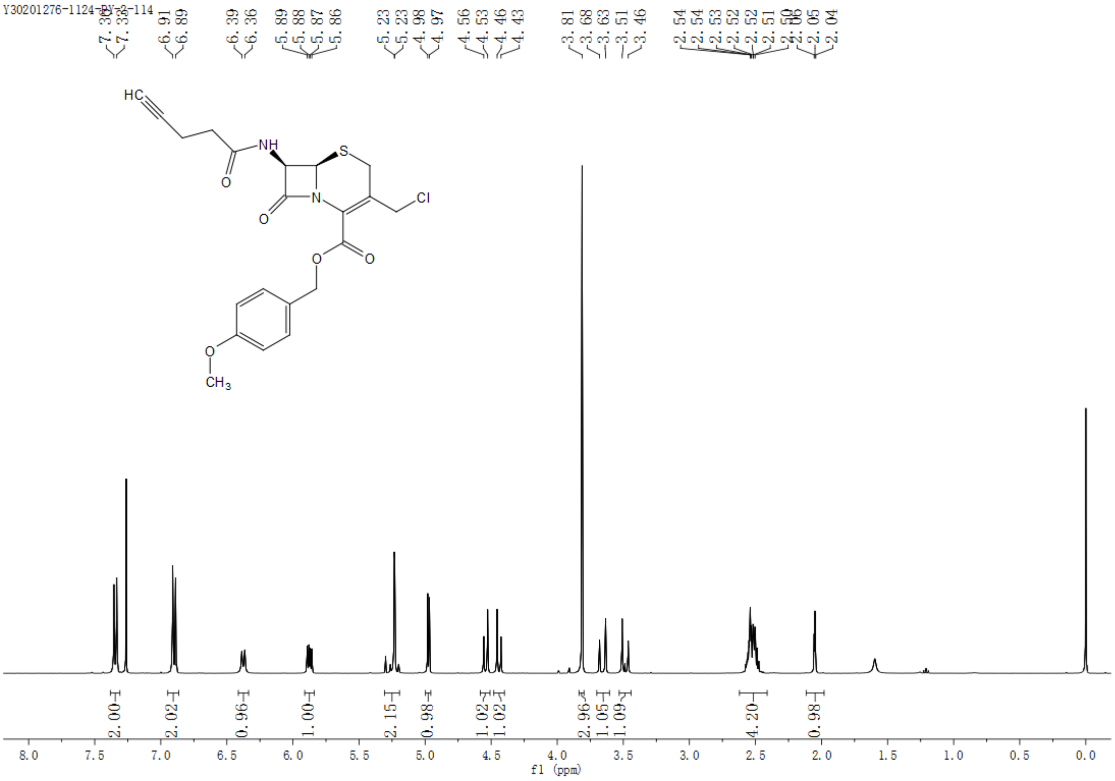

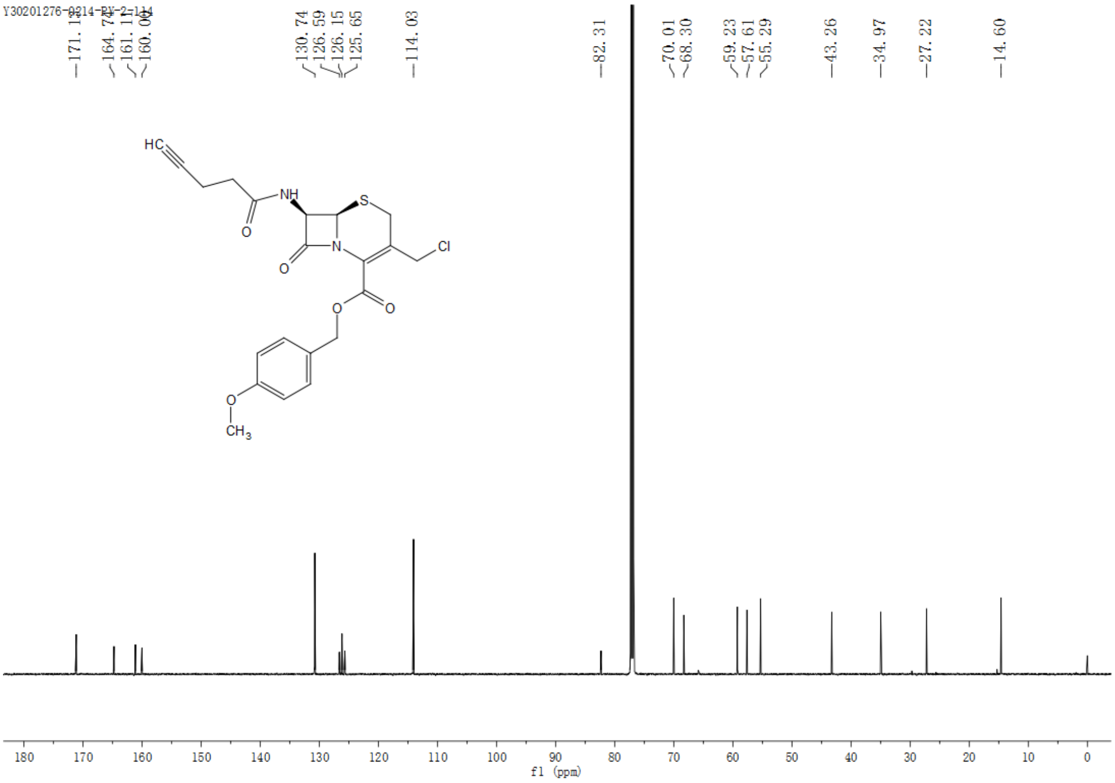

^{1}H and ^{13}C NMR Spectra of 8

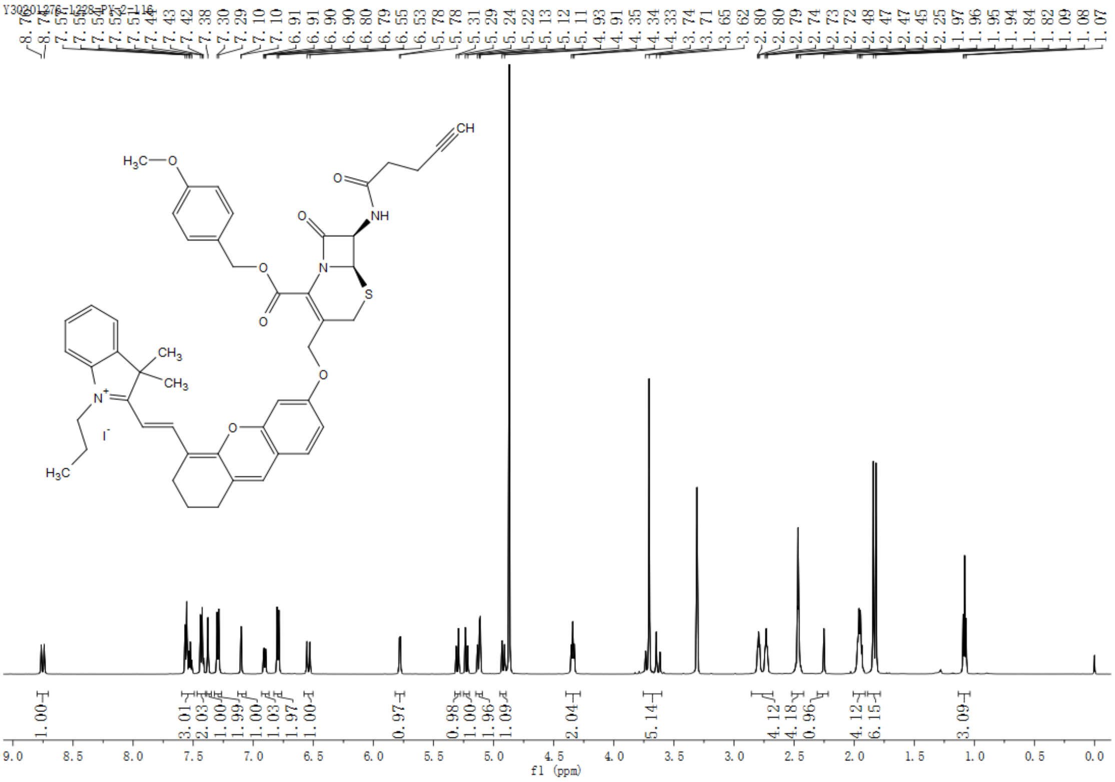

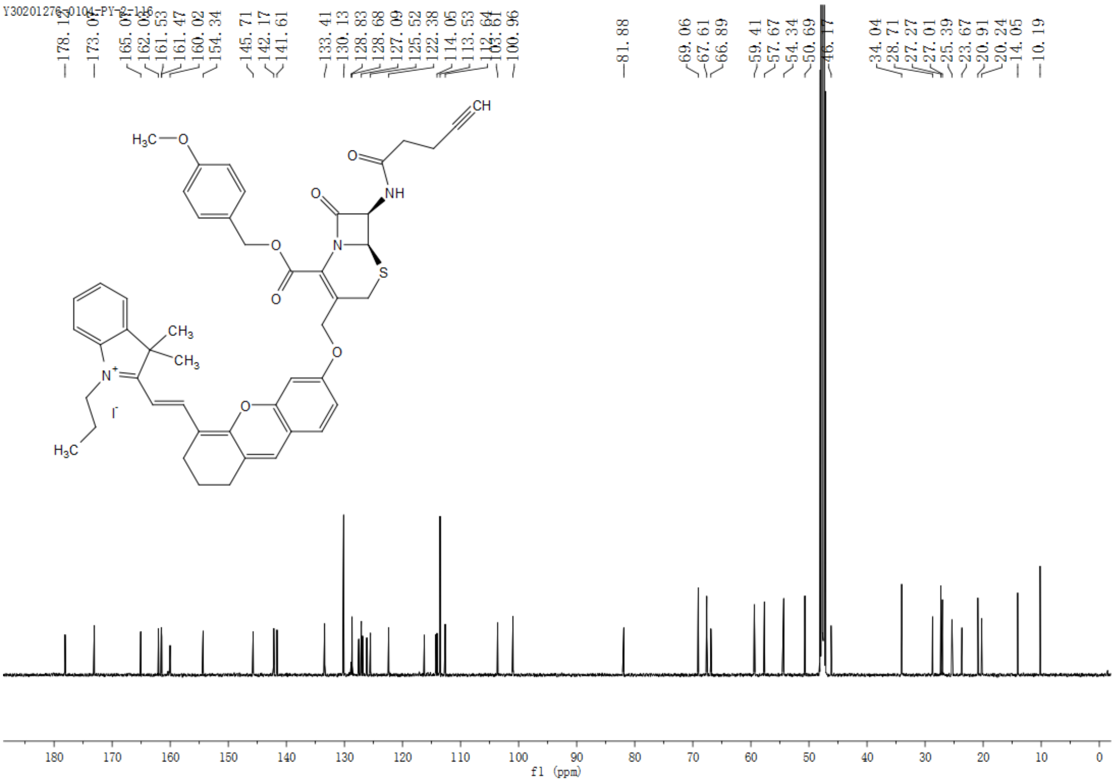

^{1}H and ^{13}C NMR Spectra of BIN-1

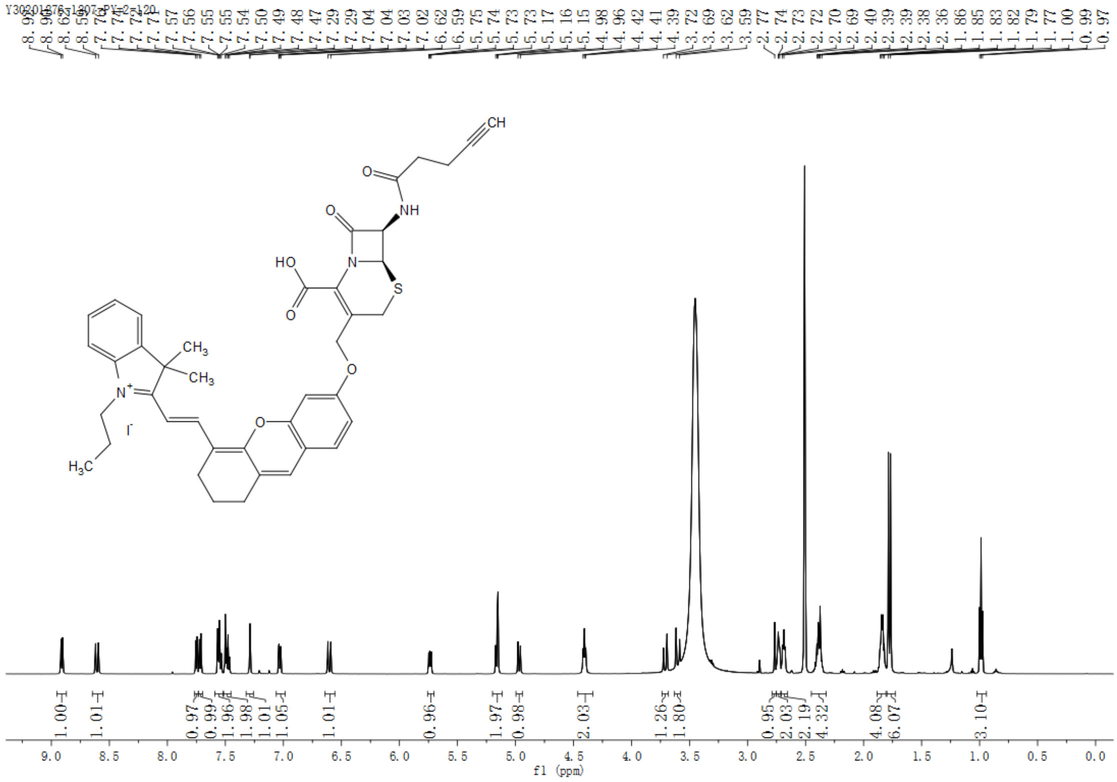

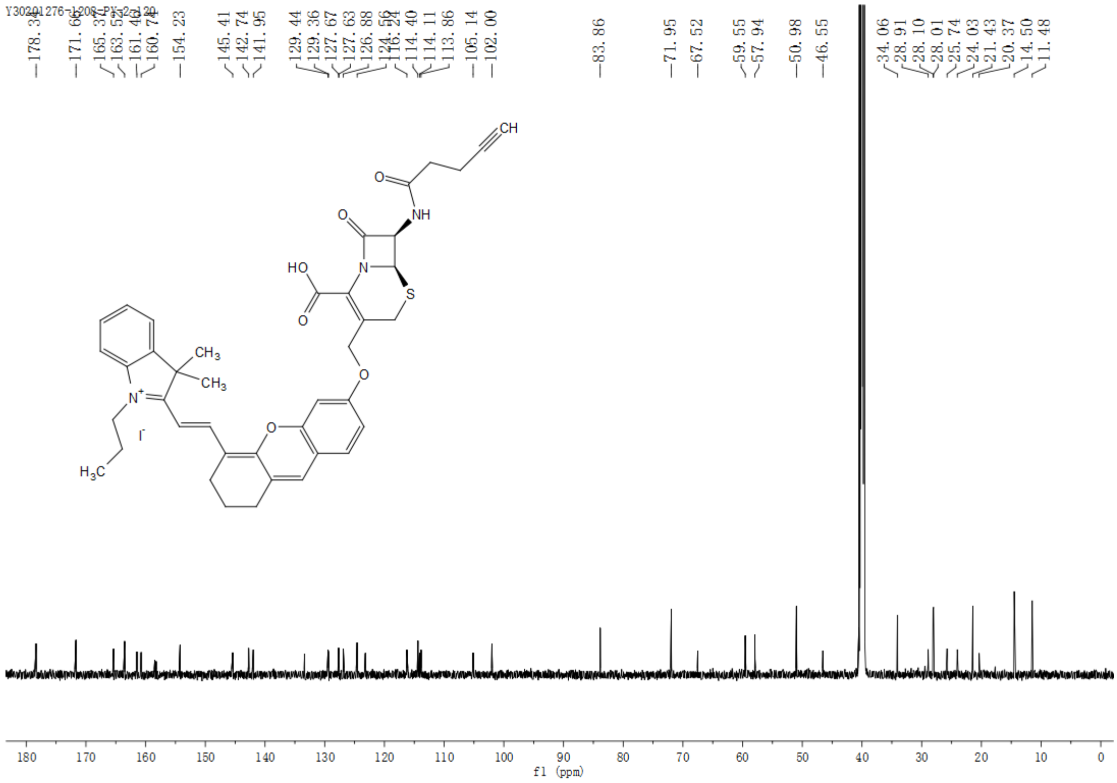

^{1}H NMR Spectra of BIN-2

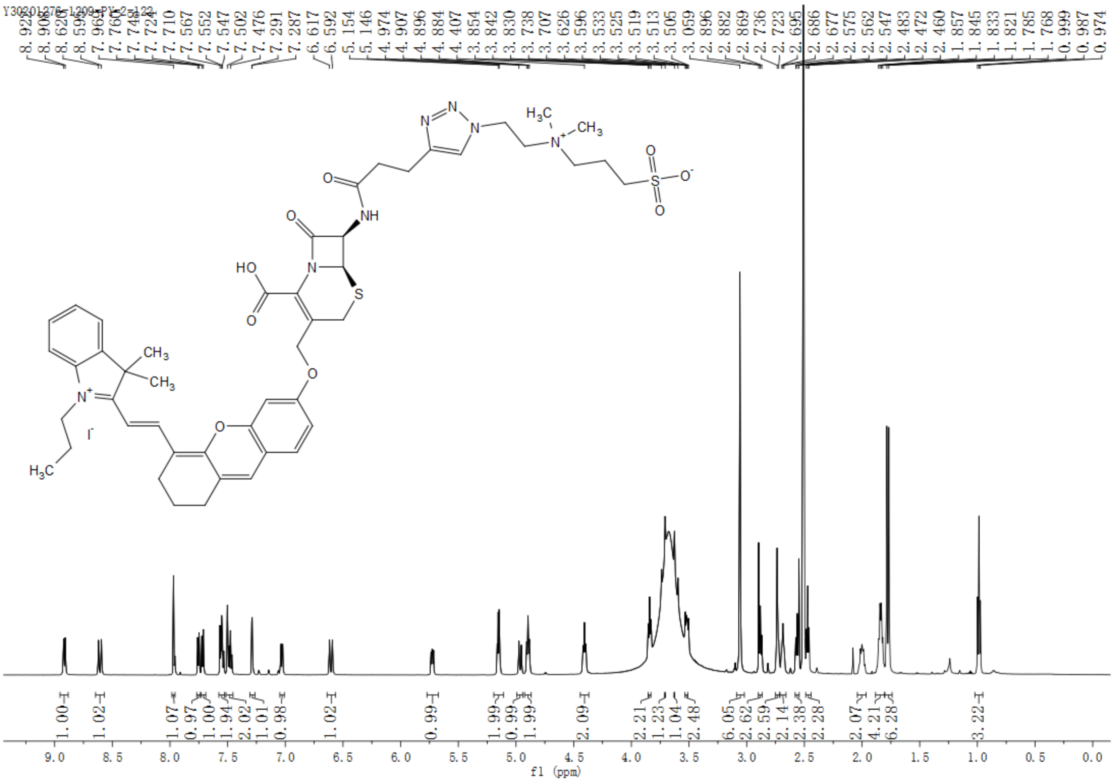

^{1}H and ^{13}C NMR Spectra of 3

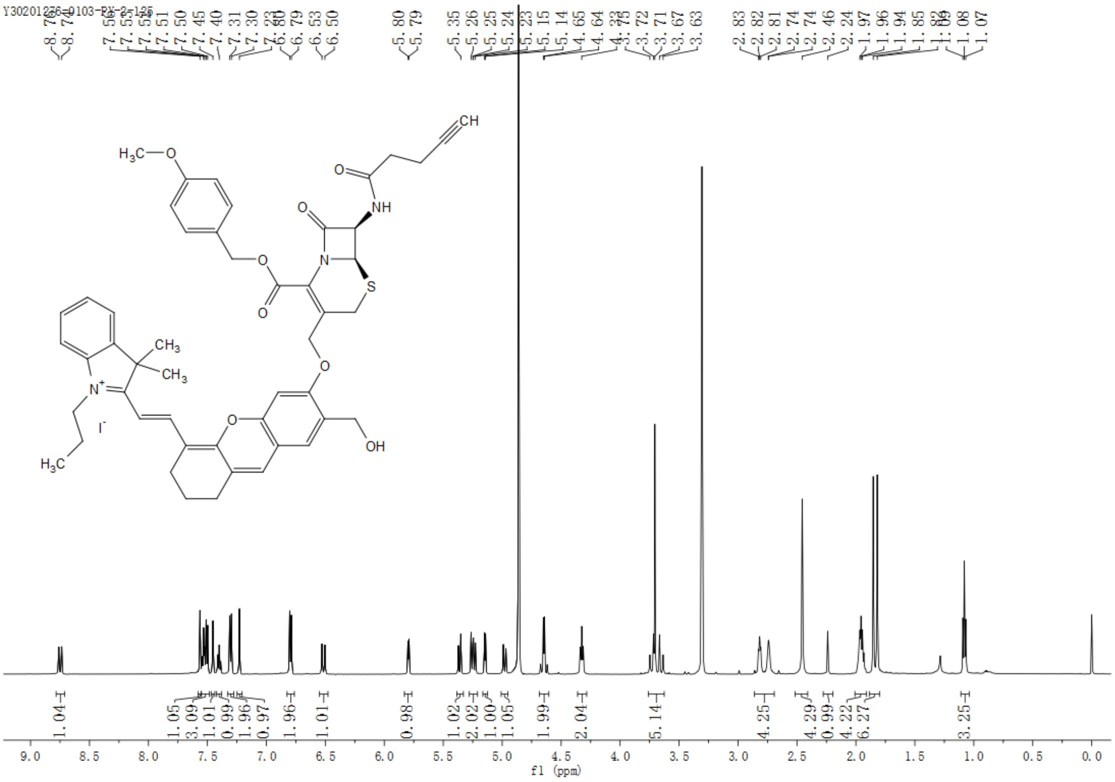

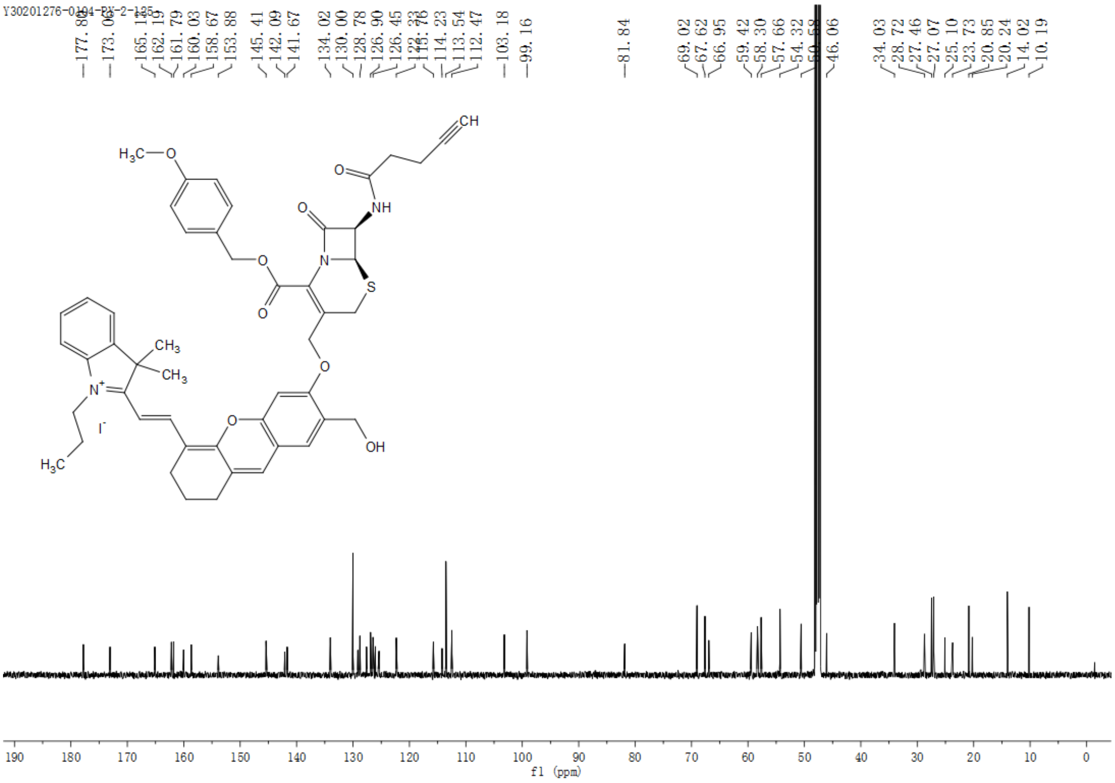

^{1}H and ^{13}C NMR Spectra of 4

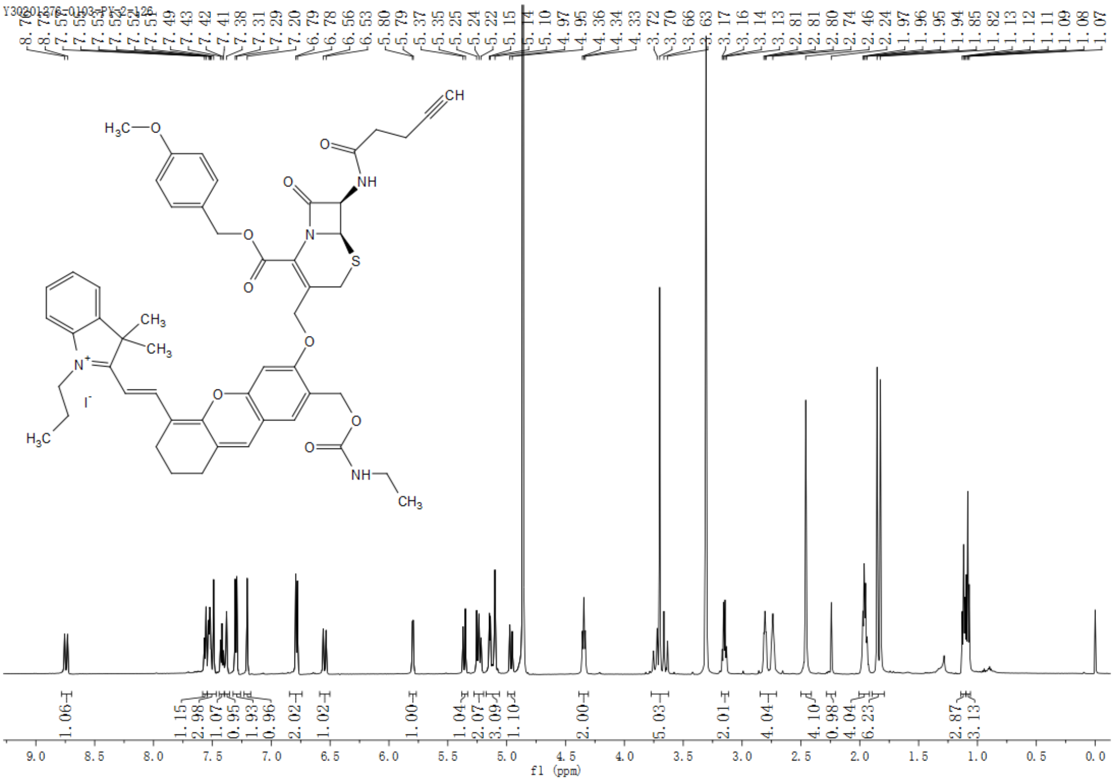

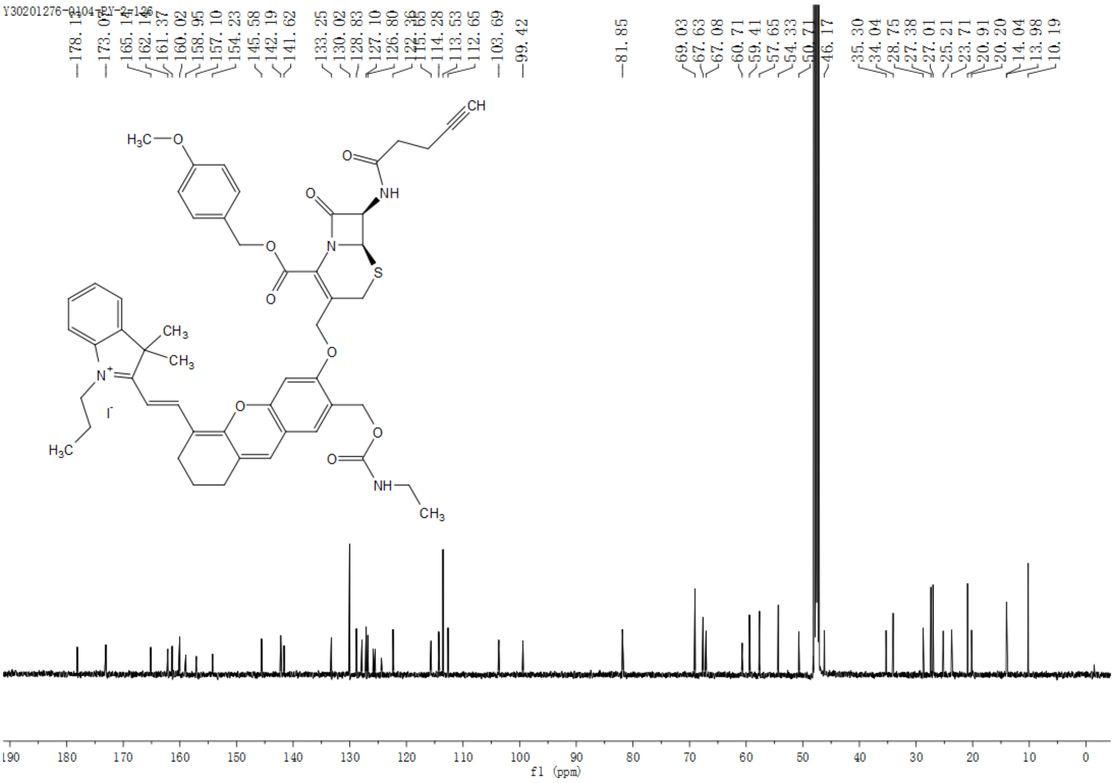

^{1}H NMR Spectra of 5

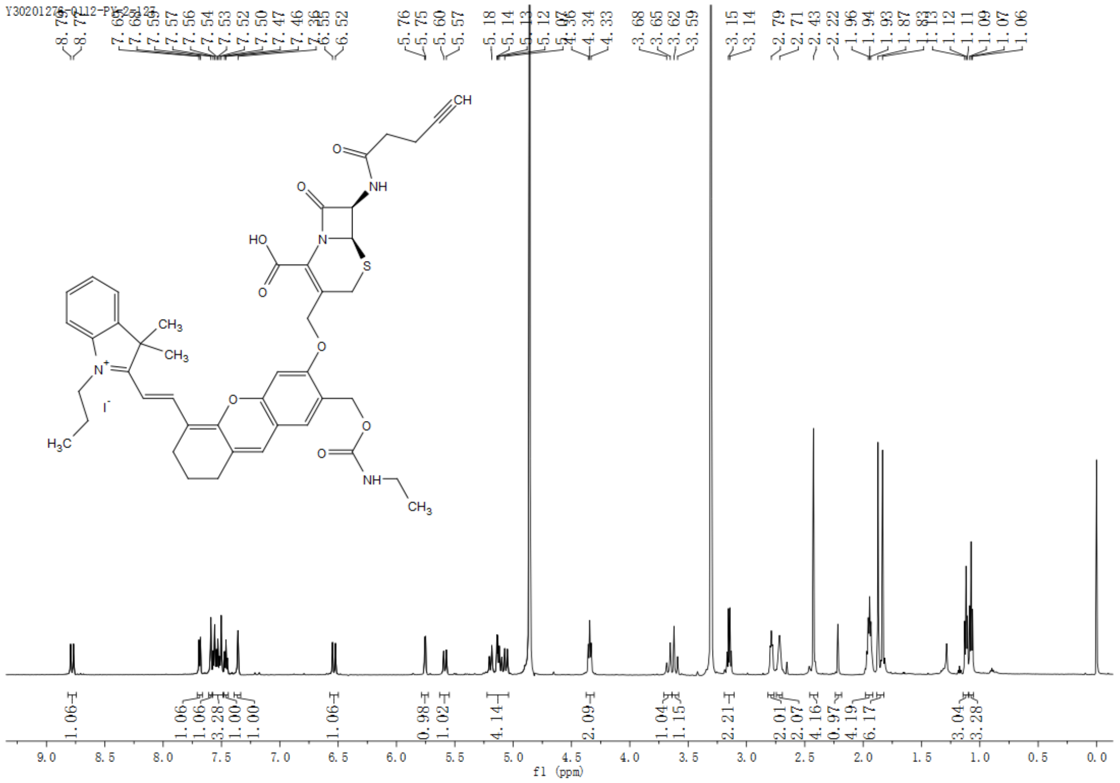

^{1}H NMR Spectra of 10

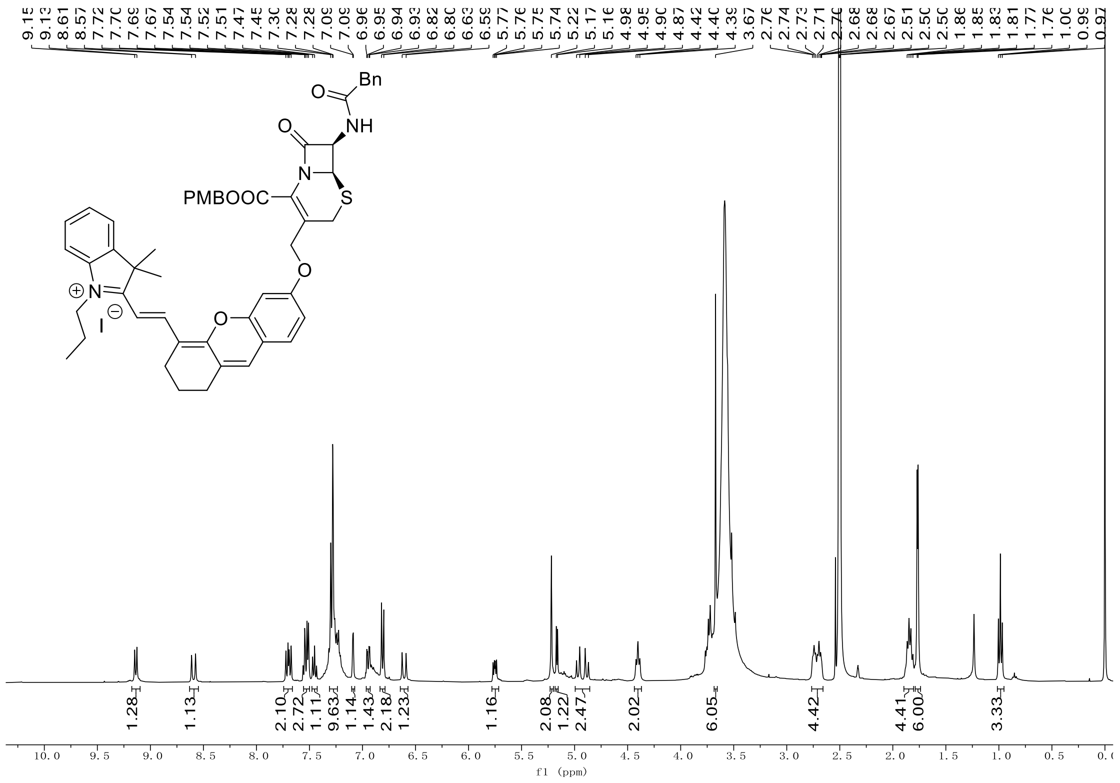

^{1}H NMR Spectra of probe 1

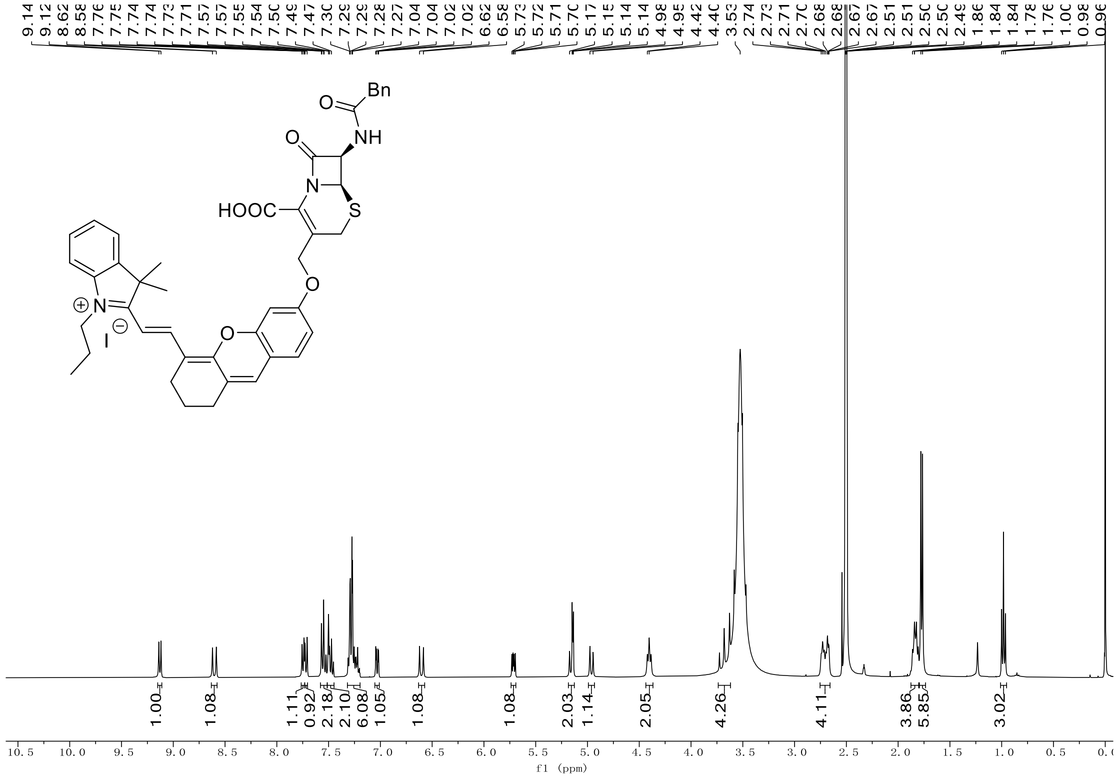

HRMS Spectra

HRMS (ESI) of BIN-1

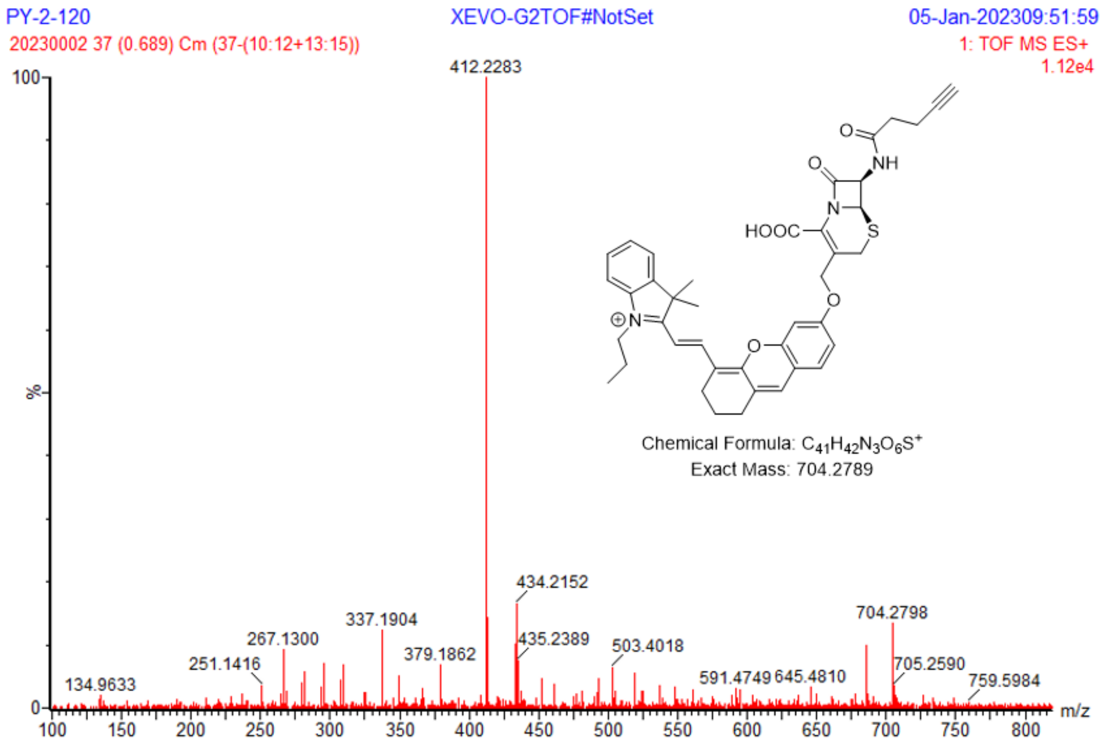

HRMS (ESI) of BIN-2

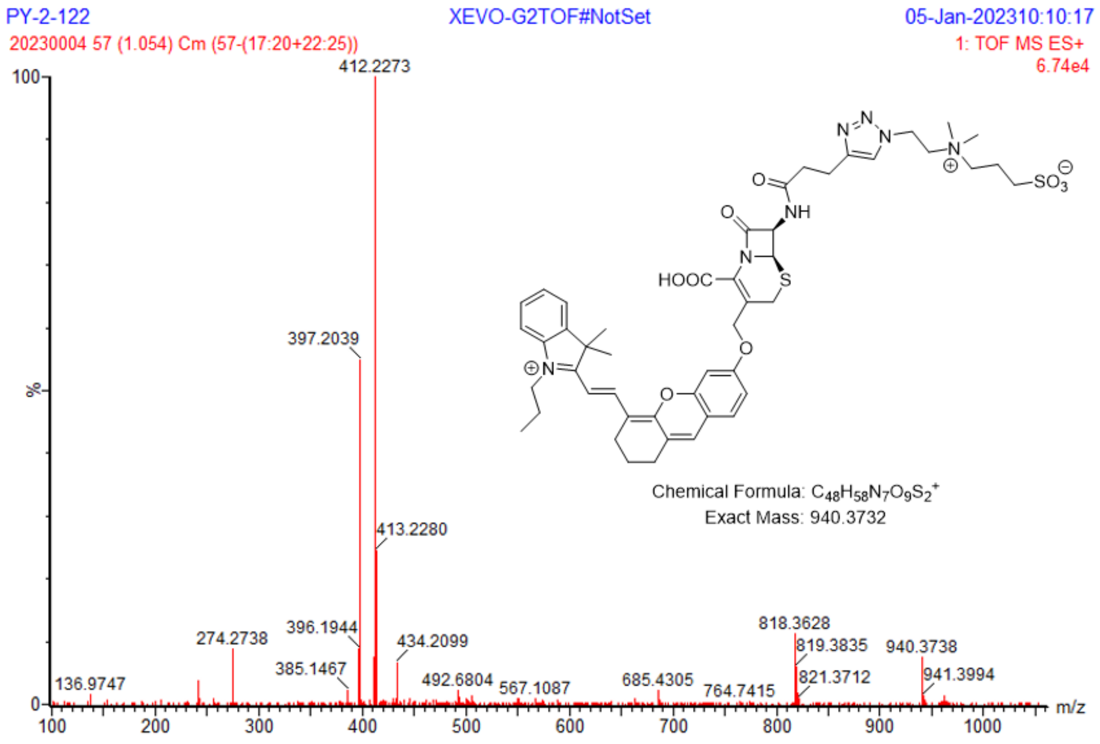

HRMS (ESI) of 3

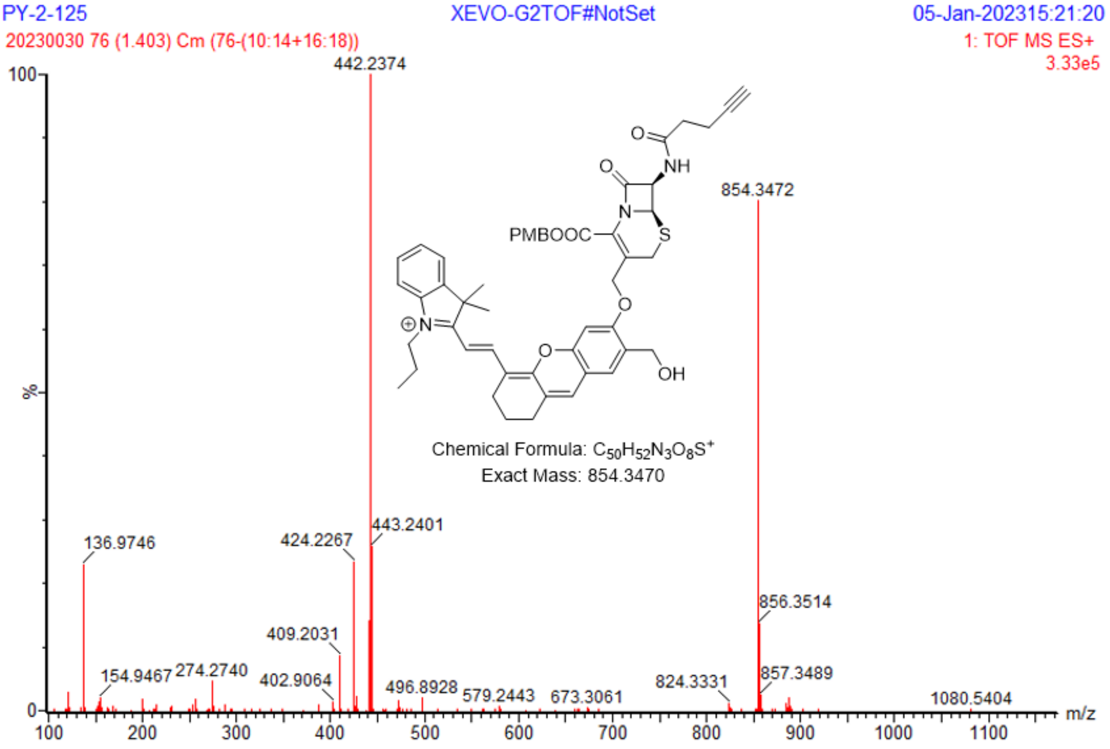

HRMS (ESI) of 4

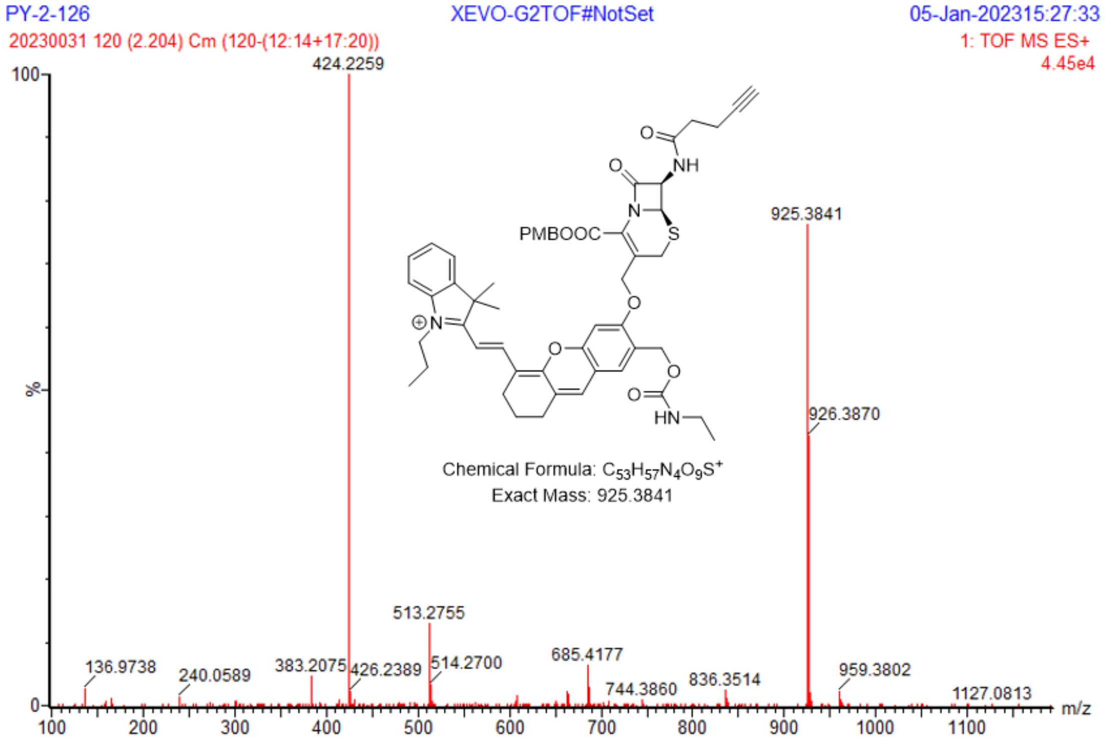

HRMS (ESI) of 5

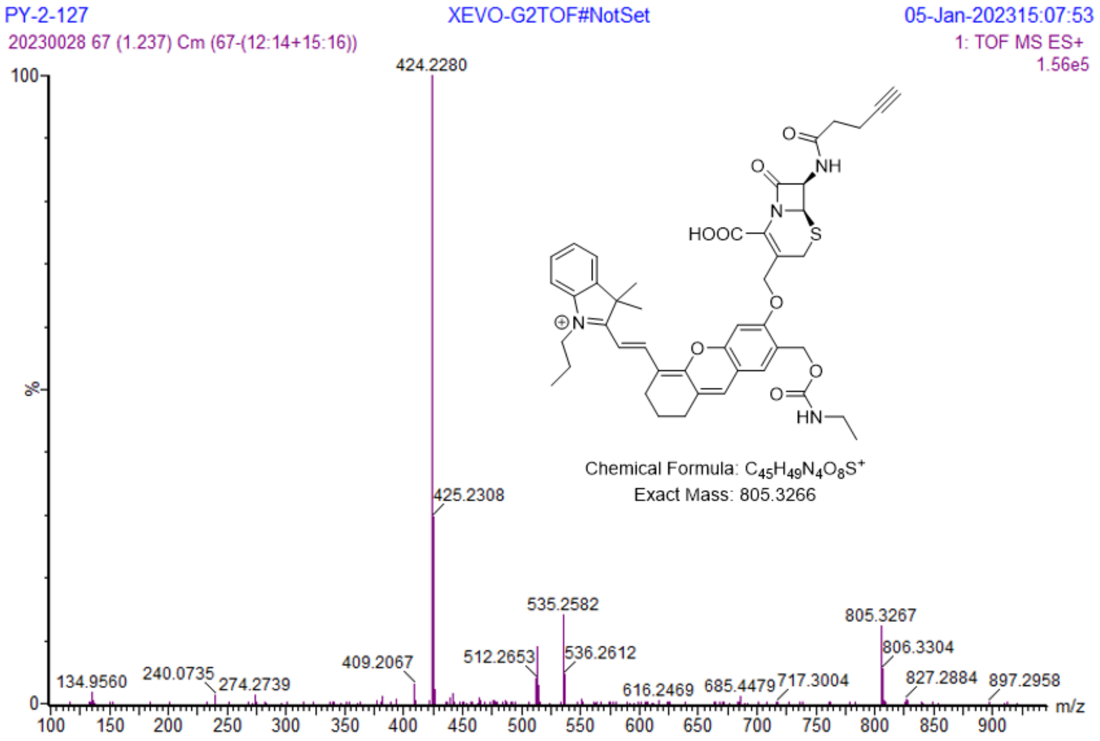

HRMS (ESI) of BIN-3

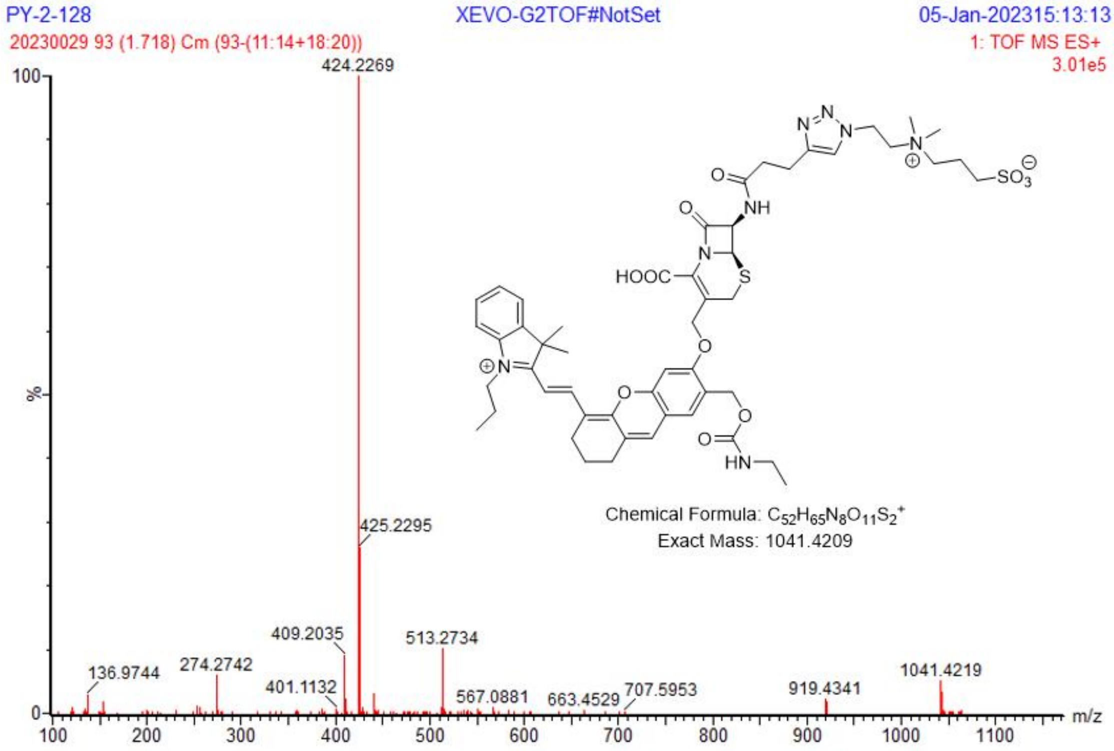

HPLC Traces

HPLC Trace of BIN-2

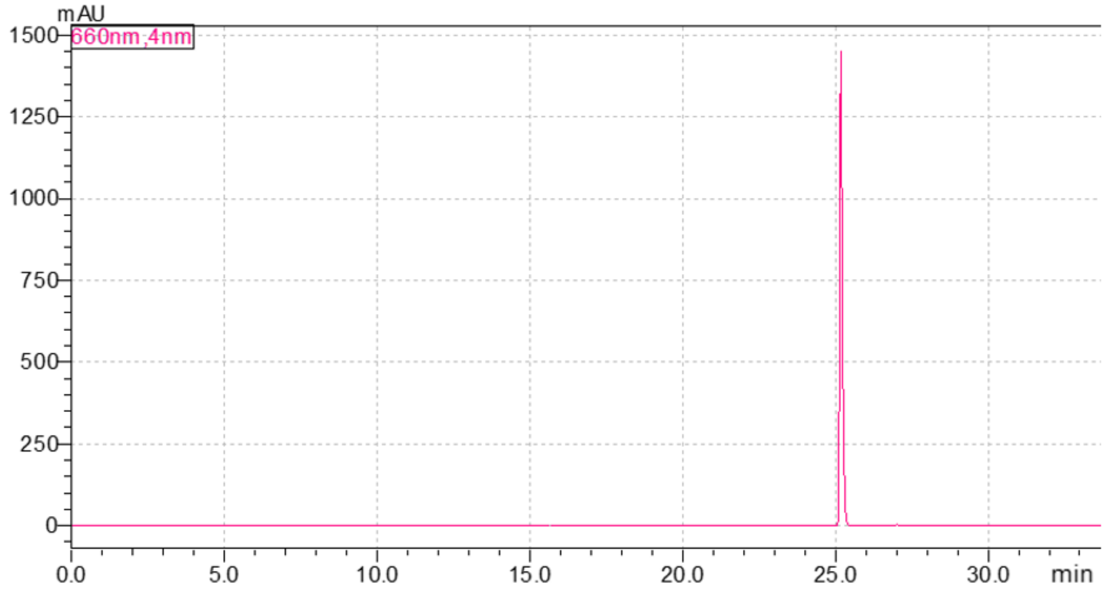

HPLC Trace of BIN-3

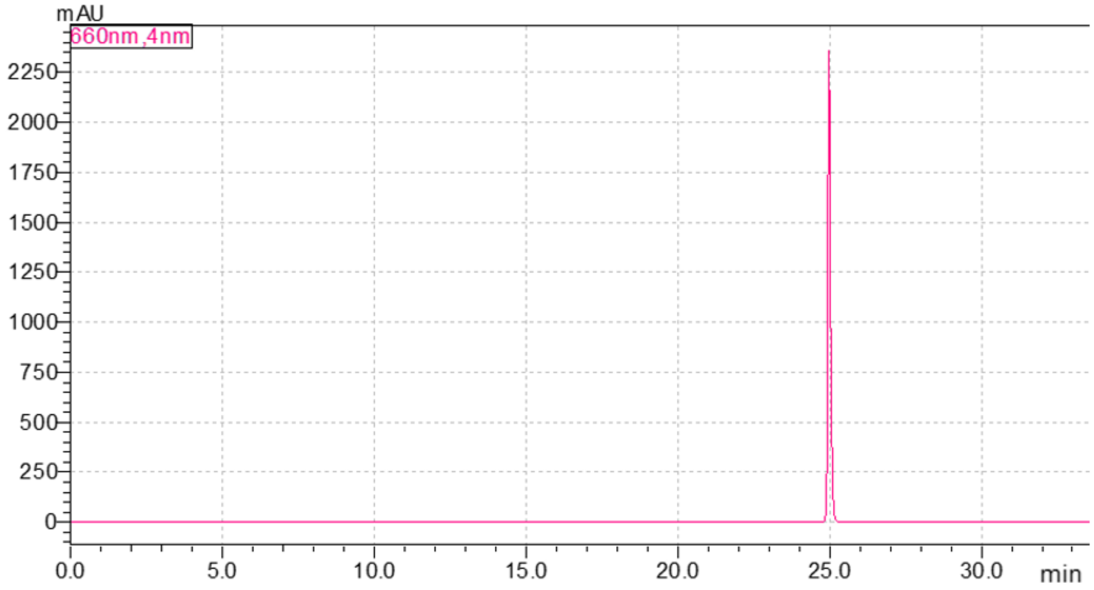

Supplement: Supplementary file 1 — Supporting Information [file ADVS-12-2408559-s001.pdf]
